# Two *Plasmodium vivax* hypnozoite-expressed RNA-binding proteins inhibit liver stage replication

**DOI:** 10.1038/s41467-026-73666-0

**Published:** 2026-05-30

**Authors:** Kim Chi Vo, Riëtte van Biljon, Gigliola Zanghi, Bryan Zavala, William Betz, Charlie Jennison, Ashley M. Vaughan, Heather J. Painter, Stefan H. I. Kappe

**Affiliations:** 1 Center for Global Infectious Disease Research, Seattle Children’s Research Institute, Seattle, WA USA; 2 Division of Bacterial, Parasitic, and Allergenic Products, Office of Vaccines Research and Review, Center for Biologics Evaluations and Research, Food and Drug Administration, Silver Spring, MD USA; 3 Department of Pediatrics, University of Washington, Seattle, WA USA; 4 Department of Global Health, University of Washington, Seattle, WA USA; 5 Present Address: Center for Vaccine Development and Global Health, University of Maryland School of Medicine, Baltimore, MD USA

**Keywords:** Mechanisms of disease, Infectious diseases

## Abstract

*Plasmodium vivax (Pv)* forms non-replicating liver stages called hypnozoites, which activate after primary infection, and cause relapses of symptomatic blood-stage malaria. We hypothesize that hypnozoites must actively suppress schizogony to maintain a quiescent state. Differential transcriptome prospecting identifies two hypnozoite-expressed transcripts encoding putative RNA-binding proteins. We assess the functional role of the two encoded proteins in *Plasmodium yoelii (Py)*, a rodent malaria parasite that naturally does not form hypnozoites. Strikingly, individual expression of each protein in *Py* liver stages blocks liver stage schizogony, with parasites remaining small and uninucleate. A screen of RNA sequences that interact with the putative RNA-binding domains of these proteins shows enrichment of distinct, highly specific motifs, indicating that they might block schizogony by binding RNAs containing these motifs. Our findings provide the unprecedented functional evidence for one potential molecular mechanism of hypnozoite formation. Based on their function we name these proteins IESI-1 and IESI-2 (Initiation of Exo-erythrocytic Schizogony Inhibited).

## Abstract (web summary)

Malaria scientists identify two candidate proteins that could maintain the *P*. *vivax* malaria parasite in a dormant hypnozoite form in the liver. These findings enhance our knowledge of malaria parasite persistence in the liver, an underexplored area important for developing new treatments.

## Introduction

Malaria remains a globally prevalent infectious disease caused by *Plasmodium* parasites, resulting in approximately 282 million clinical cases and 610,000 deaths across 80 endemic countries in 2024—an increase of 12 million cases compared with 2023 (World Malaria Report 2025)^1^. Among human malaria parasites, *P. falciparum* causes most malaria-related deaths, primarily in sub-Saharan Africa, whereas *P. vivax* (*Pv*), the most geographically widespread species, predominates in Latin America, Southeast Asia, and the Western Pacific^2^. Following mosquito transmission, *Plasmodium* sporozoites infect hepatocytes and undergo asexual replication (exo-erythrocytic or liver-stage schizogony), producing tens of thousands of merozoites, which are released into the bloodstream^3^. However, in contrast to *Pf*, a subset of *Pv* sporozoites form dormant liver stages known as hypnozoites, which can persist for weeks to years until they activate and initiate schizogony, leading to new blood stage infections (relapse) long after the primary episode^4,5^. In many endemic regions, relapses represent the predominant source of *Pv* blood-stage infections, complicating malaria control and elimination strategies^6–9^. Current relapse-prevention treatments using the drugs primaquine and tafenoquine, are contra-indicated during pregnancy as well as for individuals with glucose-6-phosphate dehydrogenase (G6PD) deficiency or CYP2D6 polymorphisms, limiting their utility^9–11^. Consequently, there is a crtitical need for novel pharmacological interventions targeting latent hypnozoites.

For years, understanding hypnozoite biology was constrained by the lack of laboratory models to study *Pv* liver stages^7,12,13^. Over the past two decades, significant advances have been made through improved infection systems, including liver-humanized mouse models^7,14^ and in vitro hepatocyte platforms, which both support *Pv* liver-stage infection and hypnozoite formation^15–17^. These models provided more insight into hypnozoite biology, including distinctive characteristics, frequency of occurrence, growth patterns, and activation processes^7,14,17^. Yet, the lack of facile *Pv* transgenesis has impeded understanding of the molecular mechanisms governing hypnozoite formation, persistence, and activation. Recently, omics-based approaches have been applied to study gene expression in *Pv*^17–19^ and *P. cynomolgi*^20–22^ (a relapsing parasite of nonhuman primates, which is also an emerging human infection). These studies have revealed transcriptome differences between replicating liver-stage schizonts and hypnozoites, highlighting enriched hypnozoite transcripts for transcriptional regulators^17,19,20^, DNA and RNA-binding proteins^17–19,22^, factors involved in translational control^20^, and pathways governing microbial dormancy, genome integrity and ATP homeostasis^21^. However, the variation in gene expression patterns across between different data sets complicates the identification of consistent, cross-species candidate genes that could regulate hypnozoite biology.

In this study, we compared *Pv*^17–19^ and *P. cynomolgi*^20–22^ liver stage transcriptomes to identify molecular regulators potentially involved in hypnozoite formation, focusing on genes consistently enriched in hypnozoites. To frame one possible molecular mechanism for liver stage dormancy, we hypothesized that hypnozoites must actively suppress the initiation of liver stage schizogony to maintain quiescence and thus prioritized transcripts encoding transcriptional or translational regulators. This analysis identified two putative RNA-binding proteins expressed in *Pv* hypnozoites, with the orthologs of these proteins also previously identified as potential candidates regulating hynozoite dormancy in *P. cynomolgi*^22^. To examine their function, we utilized trangenesis in *Plasmodium yoelii* (*Py*), a rodent malaria parasite that does not form hypnozoites. We found that both *Pv* proteins promoted a non-replicative state in *Py* liver stages by repressing liver-stage development and this is likely regulated by binding of transcripts via specific nucleotide motifs. Together, our findings show that specific RNA-binding proteins cause the active blockade of schizogony and thus are key candidate regulators of hypnozoite formation.

## Results

### Candidate gene identification and validation of differential expression in *P. vivax* liver stages

**Fig. 1:**
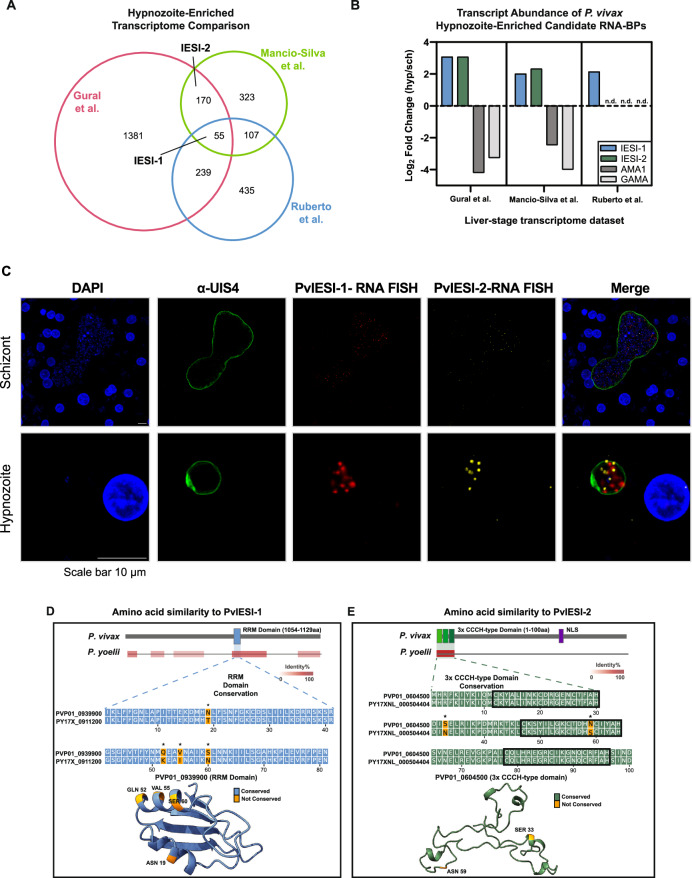
Identification and characterization of PvIESI-1 and PvIESI-2 as candidate regulators of hypnozoite development. **A** Comparison of published *P. vivax* hypnozoite transcriptomes. Differentially expressed transcripts from bulk RNA sequencing^17,18^ and single cell RNA-sequencing^19^ datasets were combined and cross-referenced to find overlapping genesets and the two candidate genes (IESI-1 and IESI-2) are highlighted. **B** The relative transcript abundance of candidate RNA-binding proteins (RNA-BPs) IESI-1 and IESI-2 enriched in hypnozoites compared to liver schizonts from three liver-stage transcriptomes^17–19^. Specific examples of transcripts increased in liver-stage schizogony (AMA1, GAMA) are shown for comparison. **C** RNA-FISH analysis of PvIESI-1 and PvIESI-2 in *P. vivax*-infected PHH cells on day 8 post-sporozoite infection, showing robust transcript abundance forPvIESI-1 (red) or PvIESI-2 (yellow) in hypnozoites but no detectable transcripts in liver stage schizonts. **D–E** Schematic of the protein structures of PvIESI-1 and PvIESI-2 with identified domains and nuclear localization signal (NLS) identified in IESI-2. Regions of conserved amino acid sequences between PvIESI and PyIESI proteins are indicated by red bars, where intensity conveys % identity for each region. Below the schematics, the alignment illustrates the conservation of the RRM and CCCH-type zinc finger domain sequences and structures in IESI proteins from *Pv* and *Py.* Non-conserved residues are indicated in orange and marked with an asterisk (*). Tertiary structures of the domains were created in AlphaFold2. Source data for this figure are provided in Source Data file 3.

Because hypnozoites are non-replicating and are thought to actively maintain their quiescence, we sought to identify molecular factors that induce and maintain this cellular state by analyzing hypnozoite-specific transcripts. We performed a comparative transcriptome analysis using three published *Pv* datasets^17–19^ (Fig. 1A, Fig. S1A) and three published *P. cynomolgi* datasets^20–22^, focusing on genes encoding RNA-binding proteins that are consistently expressed at higher levels in hypnozoites relative to replicating liver schizonts. This analysis revealed two candidate genes, PVP01_0939900 and PVP01_0604500 (Fig. 1A, B). The orthologs of these candidate genes were highly enriched in the bulk transcriptomes of hypnozoites when compared to liver schizonts, while PVP01_0939900 was also enriched in a single-cell transcriptomic study of *Pv* hypnozoites^19^ (Fig. 1B). The orthologs of PVP01_0939900 and PVP01_0604500 were enriched in *P. cynomolg*i bulk and single-cell hypnozoite transcriptomes^20,22^ (Fig. S1B). Based on phenotypic outcomes observed in our current study, we have termed these proteins as Initiation of Exo-erythrocytic Schizogony Inhibited (IESI) proteins: PvIESI-1 (PVP01_0939900) and PvIESI-2 (PVP01_0604500).

To confirm the expression patterns of PvIESI-1 and PvIESI-2 transcripts, we performed RNA fluorescence in situ hybridization (RNA-FISH) on day 8 of *Pv* liver stage development in primary human hepatocytes (Fig. 1C). Indirect immunofluorescence assays (IFA) with an UIS4 antibody were used to confirm the identity of liver stage parasites and visualize the parasitophorous vacuolar membrane (PVM) (Fig. 1C). Probes targeting PvIESI-1 and PvIESI-2 transcripts were then used and showed predominant signals in small hypnozoite forms, with minimal detection in large schizonts (Fig. 1C). These experimental findings demonstrate that PvIESI-1 and PvIESI-2 are transcribed specifically in hypnozoites, suggesting they might play a role in biological processes that are important to hypnozoite formation and persistence.

### PvIESI-1 and PvIESI-2 are predicted RNA-binding proteins

To explore the potential molecular roles of IESI-1 and IESI-2 in hypnozoite development, we analyzed their predicted protein structures. Both proteins are annotated as putative RNA-binding proteins. PvIESI-1 contains a single RRM motif (Fig. 1D) classified within the RRM_1 sub-family (PF00076), though its function remains largely uncharacterized^23^. PvIESI-2 belongs to the zinc- finger (ZnF) class of proteins, composed of three ZnF CCCH-type domains (Fig. 1E), and, like most *Plasmodium* CCCH-type proteins, its function is unpredicted due to the absence of orthologs in model species^23^.

Phylogenetic analysis showed that both IESI-1 and IESI-2 orthologs are conserved across the *Plasmodium* genus (Fig. S1C, D). Their full sequences are conserved among Asian malaria parasite species (Fig. S1C, D), though the *P. cynomolgi* IESI-1 ortholog is significantly shorter (1078 aa vs 1865 aa in *Pv*), sharing only the C-terminal region (Fig. S1C). For both proteins, the RNA-binding domains are highly conserved among divergent *Plasmodium* species (Fig. S1C, D), with the *Pv* and *Py* orthologs (PY17X_0911200 for IESI-1; PY17XNL_000504404 for IESI-2) sharing 95% and 98% identity in their RRM and CCCH domains, respectively. In fact, for IESI-2, the N-terminal zinc finger domains are the only regions of sequence conservation outside of the Asian *Plasmodium* parasite species, with sequence identity >70% for all *Plasmodium* orthologs (Fig. S1C, D). Comparatively, the full-length protein sequence alignments indicate substantial similarity between PvIESI-1 or PvIESI-2 and their corresponding orthologs in *P. cynomolgi*, at 85% and 74%, respectively, while sequence similarity with orthologs from other *Plasmodium* species range between 45% and 60% (Fig. S1C, D). The striking conservation of the RNA-binding domains, despite substantial divergence in the full-length protein sequences, suggests that RNA-binding is an essential function of these proteins.

### Transgene expression of PvIESI-1 and PvIESI-2 in *P. yoelii*

Functional genetic analysis of *Pv* genes is difficult due to the lack of a facile transgenesis system. Thus, to investigate PvIESI-1 and PvIESI-2 function in liver stages, we overexpressed the proteins by inserting an expression cassette into the dispensable PyP230p locus using CRISPR/Cas9-assisted homologous recombination. The coding sequences of PvIESI-1 and PvIESI-2 were placed under control of PyUIS4 5’ and 3’ UTRs to restrict their respective expression to pre-erythrocytic stages (^uis4^PvIESI-1 and ^uis4^PvIESI-2). For localization studies, each protein was fused at the C-terminus with an mNeon tag (^uis4^PvIESI-1^mNeon^ and ^uis4^PvIESI-2^mNeon^, Fig. S2A). Integration of these gene fusions into the PyP230p locus of clonal lines was confirmed by PCR and sequencing (Fig. S2B, C). To assess potential effects of tagging, additional constructs expressing untagged PvIESI-1 and 2 (Fig. S3A; ^uis4^PvIESI-1 and ^uis4^PvIESI-2) or mNeon alone (Fig. S3B; PyXNL^uis4-mNeon^) were generated. PCR screening with recombination-specific primers confirmed successful integration events into the PyP230p locus (Fig. S3C, D, E).

**Fig. 2:**
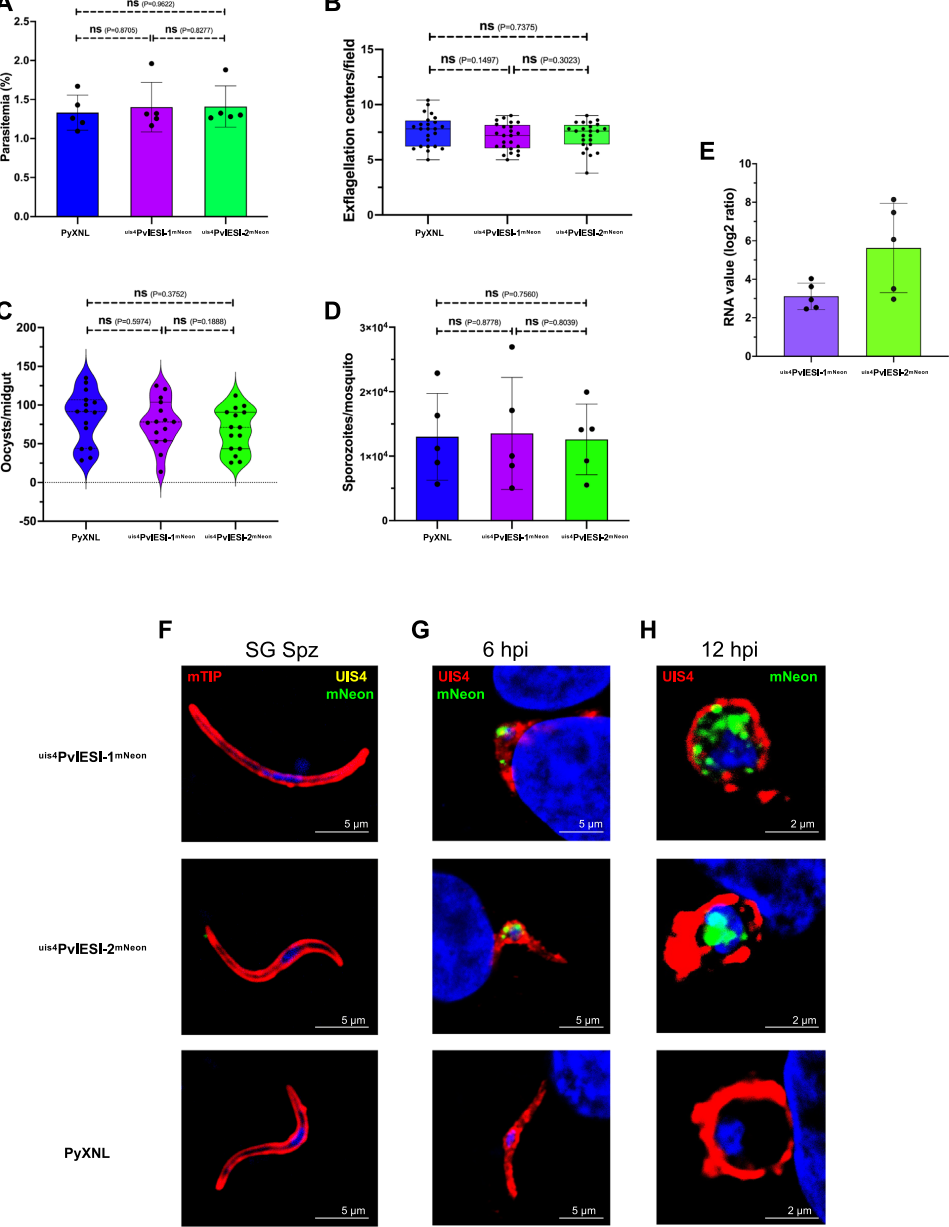
Overexpression of PvIESI-1 and PvIESI-2 does not impact development of blood stage or mosquito stage parasites. **A** Asexual blood stage parasitemia levels, **B** Male gamete emergence, **C** Mosquito infections, and **D** sporozoite counts from salivary glands showed no significant difference between lines expressing ^uis4^PvIESI-1^mNeon^ and ^uis4^PvIESI-2^mNeon^ and the wild-type PyXNL line. In **A–D**, data is presented as mean ± SD from five independent experiments (*n* = 5). Statistical analysis was carried out using two-way ANOVA with Tukey’s multiple comparison test. A *P* value > 0.05 was considered not significant (ns). In **A**) each data point represents the mean parasitemia per experiment (*n* = 5). In **B**, box plot shows the median (central line), while whiskers indicate the minimum and maximum values; individual points represent the mean of five exflagellation centers across five independent experiments (24 total data points). In **C**, violin plots display the full distribution of the data. The central line indicates the median; individual points represent the mean number of oocysts per five mosquitoes across five independent experiments (15 total data points). In **D**, each data point represents the mean number of sporozoites per mosquito from five independent experiments (*n* = 5). **E** RT-qPCR analysis of ^uis4^PvIESI-1^mNeon^ and ^uis4^PvIESI-2^mNeon^ transcript abundance in salivary gland (SG) sporozoites (Spz). Relative gene expression was calculated using the 2^−ΔΔCt^ method, with normalization to the control Py18S RNA gene. Data are presented as mean ± SD from five independent experiments (*n* = 5), each with two technical replicates. **F** Indirect immunofluorescence assay (IFA) of salivary gland sporozoites (14–15 days post-infection) showing mTIP (red; indicating inner membrane complex), mNeon (green; ^uis4^PvIESI-1^mNeon^, ^uis4^PvIESI-2^mNeon^), UIS4 (yellow), and DAPI (blue, nuclei). **G–H** IFA of ^uis4^PvIESI-1^mNeon^, ^uis4^PvIESI-2^mNeon^ and wild-type PyXNL liver stage-infected HepG2-CD81 cells at 6 hpi, and 12 hpi stained for mNeon (green) and UIS4 (red, PVM). Staining patterns for mTIP, UIS4, and mNeon were consistent across five independent experiments, from salivary gland sporozoites through liver-stage parasites at 12 hpi. Source data for this figure are provided in Source Data file 4.

To compare the life cycles of the *P. yoelii* transgenic ^uis4^PvIESI-1^mNeon^ and ^uis4^PvIESI-2^mNeon^ lines with the wildtype (WT) *P. yoelii* XNL parental line (PyXNL), infected red blood cells containing each line were intraperitoneally injected into SW mice. Parasitemia and exflagellation (microgamete emergence) were quantified on days 3–4 post infection (pi). Giemsa-stained blood smears (parasitemia 1%–2%; exflagellation across 24 random microscopic fields at 7–10 min post preparation of blood smears) indicated similar growth and gametocytogenesis rates between transgenic and WT parasites (Fig. 2A, B). The transmissibility of gametocytes to *Anopheles stephensi* mosquitoes was assessed by dissecting infected midguts 8 days post blood feeding, and 15 mosquitoes were dissected per strain for quantification of oocysts/midgut. ^uis4^PvIESI-1^mNeon^ and ^uis4^PvIESI-2^mNeon^ parasites did not show reduced oocyst numbers when compared to PyXNL (Fig. 2C). Similarly, sporozoite counts in mosquito salivary glands at days 14–15 post blood feeding showed no negative impact on sporogony and salivary gland colonization in transgenic lines (Fig. 2D).

Translation of UIS4 transcripts is repressed in sporozoites, enabling UIS4 protein synthesis only after transmission and host cell invasion^24–32^. We thus analyzed PvIESI-1 and PvIESI-2 expression in sporozoites of the ^uis4^PvIESI-1^mNeon^ and ^uis4^PvIESI-2^mNeon^ lines. We found that although the expression cassettes of both lines were transcriptionally active in salivary gland sporozoites (SG Spz) (Fig. 2E), protein was undetectable when sporozoites were probed with mNeon antibodies (Fig. 2F).

We next examined PvIESI-1 and PvIESI-2 protein expression in liver stages. Salivary gland sporozoites collected at days 14–15 post-blood feeding were used to infect HepG2-CD81 cells, and cultures were fixed at 6 and 12 hpi for staining with mNeon and UIS4 antibodies. IFA detected robust mNeon signal at 6 hpi, persisting at 12 hpi (Fig. 2G, H). while negative control assays with WT PyXNL validated specificity of the staining. These data suggests that post-transcriptional silencing of the ^uis4^PvIESI-1^mNeon^ and ^uis4^PvIESI-2^mNeon^ expression is mediated by the 5’ and/or 3’ UTR of UIS4, contrasting with *P. berghei*, where UTRs are not mediating this repression^33^. Early liver stage quantification showed no significant difference between transgenic lines and the WT PyXNL line (Figure S4A), indicating that the expression cassettes of transgenic lines did not impact sporozoite infection of host cells.

### Expression of PvIESI-1 and PvIESI-2 in *P. yoelii* perturbs liver stage to blood stage transition

We next analyzed the time to blood stage patency (prepatent period) by retro-orbital injection of 10,000 salivary gland sporozoites of each parasite line into Swiss Webster (SW) mice. Onset of blood stage parasitemia was monitored starting on day 3 post infection (Table 1). Blood stage parasites were consistently detected in all six mice infected with WT PyXNL or PyXNL^mNeon^(^uis4^mNeon) sporozoites by day 3 (Table 1), indicating that pre-erythrocytic expression of mNeon did not cause any defects in the parasite. In contrast, prepatency was prolonged to days 4–5 in mice infected with the ^uis4^PvIESI-1^mNeon^ and ^uis4^PvIESI-1 lines (Table 1), clearly indicating that expression of PvIESI-1 perturbed liver stage development. Expression of PvIESI-2 even more dramatically impaired liver-stage development. For the ^uis4^PvIESI-2^mNeon^ line, only two out of six mice developed blood-stage parasitemia, which was detectable on days 6–7 post-infection. Similarly, for the ^uis4^PvIESI-2 line, blood stage parasitemia was also observed in only two out of six mice (Table 1). These results show that PvIESI-1 and PvIESI-2 expression in *Py* liver stages severely impacts liver stage development and formation of infectious exo-erythrocytic merozoites.

**Table 1:** Overexpression of PvIESI-1 and PvIESI-2 perturbs the parasite transition from liver to blood stages

Mouse strain	Parasite strain	^a^Number of sporozoite	^b^Number of mice patent (days to patency)*
SW	PyXNL	10,000	6/6 (3)
SW	^uis4^mNeon	10,000	6/6 (3)
SW	^uis4^PvIESI-1^mNeon^	10,000	5/6 (4–5)
SW	^uis4^PvIESI-1	10,000	6/6 (4–5)
SW	^uis4^PvIESI-2^mNeon^	10,000	2/6 (6–7)
SW	^uis4^PvIESI-2	10,000	2/6 (6–7)

Sporozoite infection and blood stage transition of parasite lines expressing ^uis4^PvIESI-1^mNeon^ and ^uis4^PvIESI-2^mNeon^, lines ^uis4^PvIESI-1 and ^uis4^PvIESI-2 (both expressing proteins without mNeon) and the control ^uis4^mNeon line and wild-type PyXNL line in Swiss Webster (SW) mice. The table summarizes patency results: ^a^salivary gland sporozoites injected per mouse, and ^b^patent mice per total infected, with *days to patency in parentheses.

### Expression of PvIESI-1 and PvIESI-2 inhibit exo-erythrocytic schizogony in *P. yoelii*

**Fig. 3:**
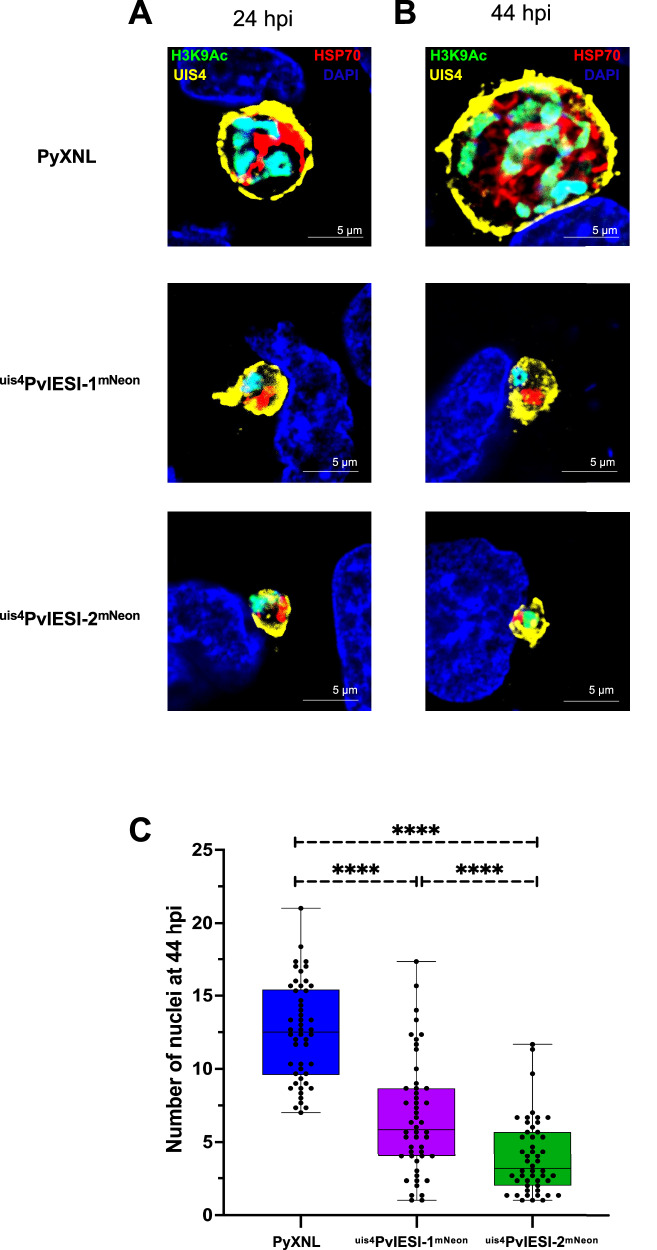
Overexpression of PvIESI-1 and PvIESI-2 exhibits the know hallmarks of hypnozoite and perturbs nuclear replication of liver stage parasites. **A, B** Indirect immunofluorescence assays (IFA) were performed on ^uis4^PvIESI-1^mNeon^ and ^uis4^PvIESI-2^mNeon^ overexpression lines in comparison with WT PyXNL parasites. Overexpression lines exhibited features consistent with a hypnozoite-like phenotype. Parasites were analyzed at 24 hpi **A** and 44 hpi **B** using the acetylated histone marker H3K9Ac (green), which indirectly marks nuclei, UIS4 (yellow) to visualize PVM development, and HSP70 (red) to visualize the mitochondria. Staining and visualization patterns were consistent across all markers in both overexpression and WT lines in three independent experiments. **C** the graph shows enumeration of nuclei at 44 hpi across parasite lines. Data are presented as mean ± SD from three independent experiments (*n* = 3). For each group, 150 *P. yoelii* liver-stage parasites (50 parasites for each independent experiment) were counted and assessed for nuclear number. Statistical analysis was carried out using two-way ANOVA with Tukey’s multiple comparison test. A *P* value of < 0.05 was considered statistically significant, ****P < 0.0001. Box plots show the median (central line), while whiskers indicate the minimum and maximum values. Individual data points represent the mean nuclear number per three parasites, aggregated across three independent experiments (50 total data points). Source data for this plot are provided in Source Data file 4.

Hypnozoites are notably distinguished from replicating liver stage schizonts by their small size^8^, minimal DNA replication, and underdeveloped organelles^7,17^. To assess the liver stage phenotypes of *Py* when PvIESI-1 or PvIESI-2 are expressed, IFAs were performed on ^uis4^PvIESI-1^mNeon^ and ^uis4^PvIESI-2^mNeon^ lines and compared to WT PyXNL line at 24 hpi (Fig. 3A) and 44 hpi (Fig. 3B) in HepG2-CD81 cells. IFAs of liver stages showed strong circumferential UIS4 staining of at the parasitophorous vacuole membrane (PVM) (Fig. 3A, B). Strikingly, transgenic parasites remained small at both time points, whereas WT parasites exhibited robust growth. An antibody specific for histone acetylation (H3K9Ac) was used to indirectly assess DNA replication. Remarkably, the small ^uis4^PvIESI-1^mNeon^ and ^uis4^PvIESI-2^mNeon^ liver stage parasites consistently displayed a single acetylated histone-positive structure, clearly indicating minimal genome replication and segregation at 24 hpi (Fig. 3A) and 44 hpi (Fig. 3B), while WT parasites progressively accumulated multiple H3K9Ac-postive structures (Fig. 3A, B). Co-staining of DNA with DAPI and H3K9Ac revealed co-localization and allowed quantification of DNA centers at 44 hpi across 150 liver-stage parasites for each strain (WT average = 12.5, ^uis4^PvIESI-1^mNeon^ = 6.7 and ^uis4^PvIESI-2^mNeon^ = 4) (Fig. 3C), further demonstrating that PvIESI-1 and PvIESI-2 expression suppresses DNA replication and liver stage growth.

Because *Plasmodium* parasites possess a single mitochondrion that undergoes extensive branching during schizogony, we examined mitochondrial morphology in transgenic liver stages using a mitochondrial HSP70 antibody. As liver stage schizogony advanced, the WT PyXNL line exhibited progressive growth and mitochondrial branching. Conversely, only a single mitochondrial structure was consistently observed in a subset of parasites in the ^uis4^PvIESI-1^mNeon^ and ^uis4^PvIESI-2 ^mNeon^ lines (Fig. 3A, B), strongly suggesting a significant suppression of organelle expansion and development.

**Fig. 4:**
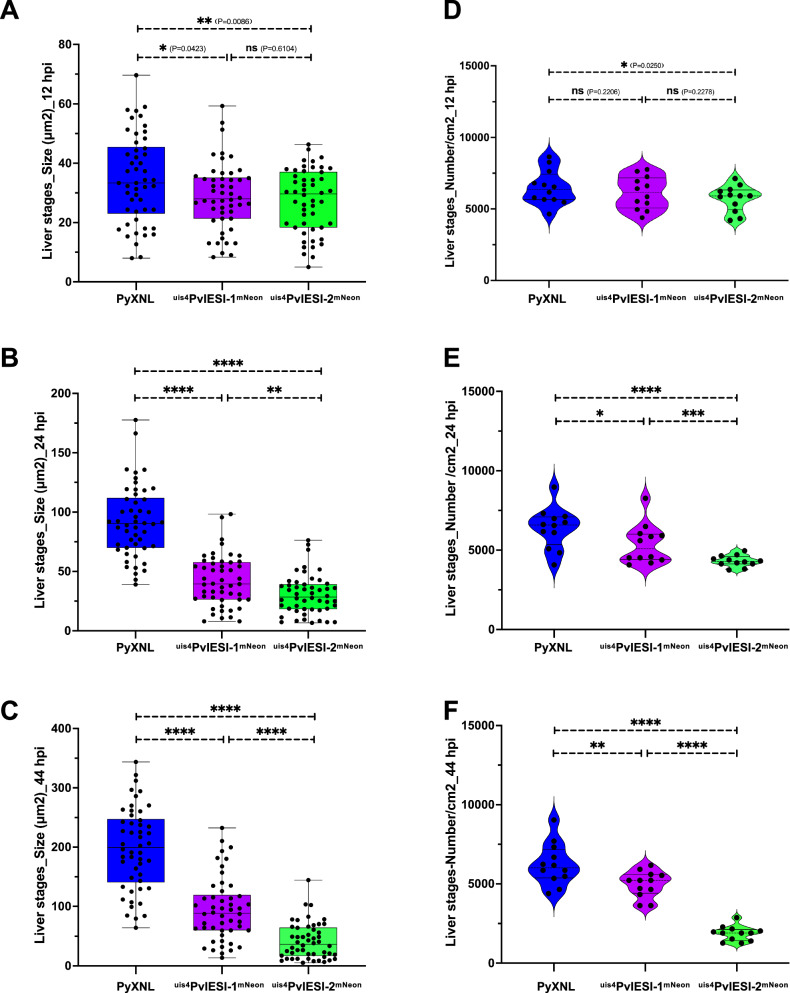
Overexpression of PvIESI-1 and PvIESI-2 progressively inhibit exo-erythrocytic schizogony and impact parasite survival. **A–C** Liver-stage parasite size shows reduced growth in parasites expressing PvIESI-1 or PvIESI-2 compared to the control PyXNL across the developmental time course from 12 to 24 h post-infection in HepG2-CD81 cells. Data are presented as mean ± SD from three independent experiments (*n* = 3). For each group, the sizes of 150 *P. yoelii* liver-stage schizonts were measured, with 50 parasites analyzed per independent experiment. Statistical analysis was carried out using two-way ANOVA with Tukey’s multiple comparison test. A *P* value of <0.05 was considered statistically significant. In **B**, **P = 0.0016, ****P < 0.0001; in **C**, ****P < 0.0001. The box plots show the median (central line), with whiskers indicating the minimum and maximum values. All individual data points are shown, with each point represents the mean size of three parasites across three independent experiments (50 data points in total). **D–F** Parasite numbers were quantified over the developmental time course in parasites expressing PvIESI-1 or PvIESI-2 and compared with the PyXNL control from 12 to 24 h post-infection in HepG2-CD81 cells. A progressive reduction in parasite numbers was observed over time, with a more pronounced defect in the PvIESI-2–expressing line. Data are presented as mean ± SD from four independent experiments (*n* = 4). For each condition, 12 randomly selected fields per 8-well plate were imaged at 20× magnification using a Keyence microscope and analyzed for parasite counts. Statistical significance was assessed using two-way ANOVA followed by Tukey’s multiple comparisons test, with *P* < 0.05 considered significant. In **E**, *P = 0.0296, ***P = 0.0004, and ****P< 0.0001; in **F**, **P = 0.0067 and ****P < 0.0001. Violin plots depict the full distribution of the data, with the central line indicating the median. All individual data points are shown, with each point represents the mean number of parasites per region per cm² (12 regions per cm² in total) across four independent experiments. Source data for this figure are provided in the Source Data file 4.

We quantitatively analyzed liver-stage growth of ^uis4^PvIESI-1^mNeon^ and ^uis4^PvIESI-2^mNeon^ at different time points in vitro. IFAs and size measurements revealed that expression of PvIESI-1 and PvIESI-2 resulted in stunted liver stage development. At 12 hpi, the size differences between the WT PyXNL and the transgenic lines were minimal (Fig. 4A). However, by 24 hpi, a significant reduction in liver stage size was observed for both transgenic lines when compared to WT PyXNL (Fig. 4B). At 44 hpi, most WT PyXNL parasites had grown into well developed liver stage schizonts. In stark contrast, ^uis4^PvIESI-1^mNeon^ and ^uis4^PvIESI-2^mNeon^ liver stages exhibited continued growth arrest and maintained a population of small parasites (< 5 μm) (Fig. 4C). Interestingly, this growth arrest was more pronounced for the ^uis4^PvIESI-2^mNeon^ liver stages (Fig. 4B, C), potentially explaining its more severe impact on blood stage transition. Similar phenotypes were also observed with ^uis4^PvIESI-1 and ^uis4^PvIESI-2 liver-stage lines lacking mNeon (Fig. S4B), confirming that growth arrest is specifically attributable to PvIESI-1 and PvIESI-2 expression.

We next quantified liver stage parasite numbers in transgenic lines compared to WT PyXNL over time. At 12 hpi, comparative liver stage parasite numbers were similar for transgenic and WT lines (Fig. 4D). However, by 24 and 44 hpi, the transgenic lines showed a decline in liver stage numbers compared to WT PyXNL, with a more pronounced declines observed for ^uis4^PvIESI-2^mNeon^ liver stages (Fig. 4E, F).

### PvIESI-1 and PvIESI-2 localize to liver stage cytoplasmic stress granules

Both PvIESI-1 and PvIESI-2 harbor RNA-binding domains (Fig. 1D, E), and their individual expression in *Py* liver stages inhibits liver-stage schizogony. One mechanism by which they might excerpt this effect is translational control via regulation of RNA stability and translational repression, which in *Plasmodium* spp. occurs in stress granules^34–37^. To examine the localization of PvIESI-1 and PvIESI-2, we performed IFAs on ^uis4^PvIESI-1^mNeon^ and ^uis4^PvIESI-2^mNeon^ liver-stages in HepG2-CD81 cells. The mNeon signal indicated that PvIESI-1 and PvIESI-2 predominantly localize to discrete punctate structures in the cytoplasm similar to RNA storage granules (Fig. 2G, H; Fig. S4C, D).

**Fig. 5:**
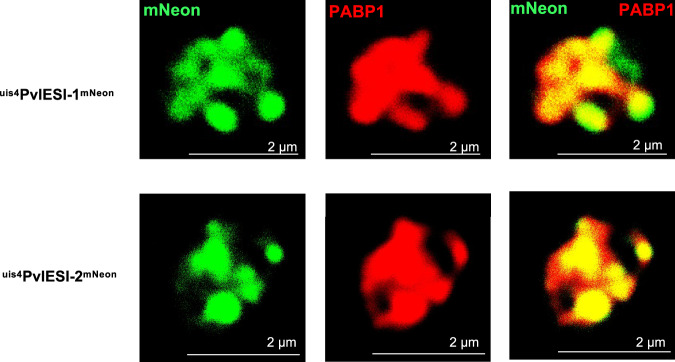
PvIESI-1 and PvIESI-2 localize to cytoplasmic stress granules during liver-stage development. Immunofluorescence analysis (IFA) of HepG2-CD81 cells infected with ^uis4^PvIESI-1^mNeon^ and ^uis4^PvIESI-2^mNeon^ parasites at 24 hpi. Parasites were stained for mNeon (green) and the stress granule marker PABP1 (red). Co-localization of PvIESI-1 and PvIESI-2 with PABP1 indicates their association with cytoplasmic stress granules. This co-localization pattern and fluorescence staining were consistently observed across three independent experiments.

We next compared PvIESI-1 and PvIESI-2 localization to that of poly (A)-binding protein 1 (PABP1), a protein known to associate with translational repression complexes, including those found in stress granules in *Plasmodium* spp^38,39^. As PABP1 binds to the poly (A) tail of mRNAs, its distribution highlights regions where mRNA is actively localized^40,41^. We observed strong co-localization of the PvIESI-1^mNeon^ and PvIESI-2^mNeon^ signals with PABP1 (Fig. 5), further substantiating their localization to stress granules.

### Overexpression of the RNA-binding protein PUF2 does not induce liver stage arrest

**Fig. 6:**
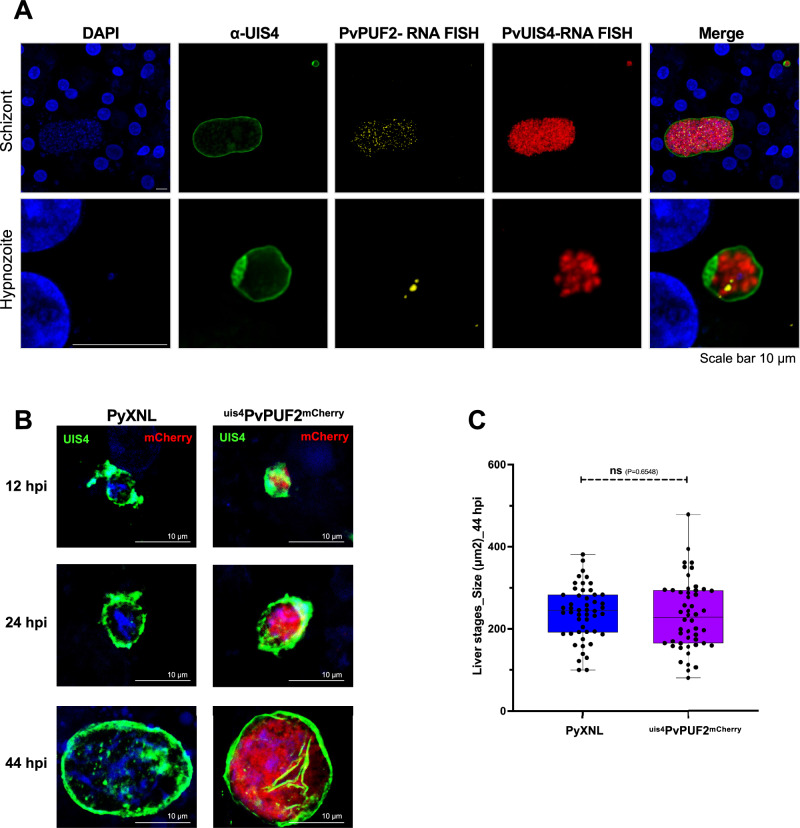
Overexpression of PvPUF2 during liver stage development does not affect parasite growth and liver-to-blood transition. **A** RNA-FISH analysis of PvPUF2 transcripts in *P. vivax* liver stage-infected PHH cells at day 8 post-sporozoite infection. Parasites were labeled with α-UIS4 antibody (green) to mark the PVM. The RNA probe targeting PvPUF2 transcripts (yellow) shows strong signal in both hypnozoites and schizonts. A probe against UIS4 transcripts (red) was included as a control for the RNA-FISH assay. **B** PvPUF2 protein expression during liver stage development in transgenic ^uis4^PvPUF2^mCherry^ *P. yoelii*. ^uis4^PvPUF2^mCherry^ parasites expressing PvPUF2-mCherry were examined from 12 to 44 hpi. α-UIS4 antibody (green) marks the PVM and identifies parasites, while the mCherry signal indicates PvPUF2 protein expression. The expression patterns of UIS4 (green) and PvPUF2-mCherry (red) were consistently observed in both the overexpression lines and the PyXNL line across four independent experiments. **C** Liver-stage parasite size at 44 hpi. No significant difference in parasite size was observed between the ^uis4^PvPUF2^mCherry^ line and the control PyXNL line. Data are presented as mean ± SD from three independent experiments (*n* = 3). For each group, the sizes of 150 *P. yoelii* liver-stage schizonts were measured, with 50 parasites analyzed per independent experiment. Statistical analysis was carried out using two-way ANOVA with Tukey’s multiple comparison test. A *P* value of >0.05 was considered not significant (ns). The box plots show the median (central line), with whiskers indicating the minimum and maximum values. All individual data points are shown, with each point representing the mean size of three parasites across three independent experiments (50 data points in total). Source data for this figure are provided in the Source Data file 4.

To address the possibility that overexpression of any *Plasmodium* RNA-binding protein in liver stages could nonspecifically arrest parasite development, potentially through excessive sequestration of mRNAs required for parasite viability, we performed a control experiment using an RNA-binding protein unrelated to IESI-1 and IESI-2. We selected PvPUF2, a well-characterized member of the PUF family containing a conserved PUM domain^42–44^. Bulk transcriptomic data from Gural et al.^17^ show that PvPUF2 transcripts were upregulated in hypnozoites relative to schizonts (Fig. S5A), indicating that this RNA-binding protein is naturally expressed during liver-stage development. We confirmed this expression pattern by RNA-FISH analysis, which detected PvPUF2 transcripts in both hypnozoites and schizonts (Fig. 6A). Using the same strategy applied for PvIESI-1 and 2, we generated a transgenic line overexpressing PvPUF2 fused to mCherry under the control of the UIS4 promoter (Fig. S5B) and confirmed correct genomic integration (Fig. S5C). Overexpression of PvPUF2 had no observable impact on blood stage development (Fig. S5D), gametocytogenesis (Fig. S5E), oocyst formation (Fig. S5F) or sporogony (Figure S5G). UIS4 promoter control ascertained PvPUF2 protein expression throughout liver stage development, which was visualized using antibodies to mCherry (Fig. 6B). Yet, despite robust liver stage expression of PvPUF2, parasite growth and maturation were indistinguishable from the PyXNL control, with comparable liver-stage parasite sizes 44 hpi (Fig. 6C). Consistent with normal liver stage progression, patent blood-stage infection occurred three days after sporozoite inoculation, with no delay relative to control (Table S1). These results show that overexpression of any RNA-binding protein does not inevitably impair parasite liver stage development, further ascertaining the specificity of the phenotype observed for PvIESI-1 and 2 expressions.

### IESI-1 and IESI-2 bind sequence-specific RNA

**Fig. 7:**
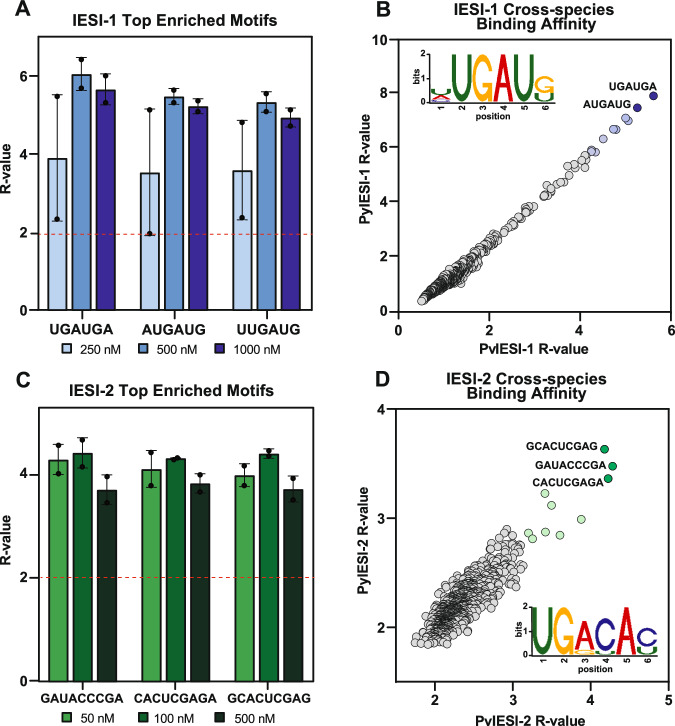
Determining the in vitro RNA binding specificity of IESI-1 and IESI-2. Results from RBNS shows the top motifs enriched above occurrences of all possible 6-mers as R-values^45^ with significance threshold indicated with dashed red line and individual technical replicates (mean of *n* = 2 ± SD) indicated as closed circles for PvIESI-1 **A** and PvIESI-2 **C** respectively. Correlation between the average enrichment (R-values) obtained for motifs in the *P. yoelii* and *P. vivax* IESI-1 **B** and IESI-2 **D** experiments. Representative sequence logo of 10 top motifs enriched in RBNS was generated in seqlogo in R. Source data for this figure are provided in the Source Data file 1.

Given the importance of both PvIESI-1 and PvIESI-2 to liver stage development, we investigated whether these proteins function as mRNA interactors based on predicted RNA-binding domains. Since functional studies in *Pv* liver stages are challenging, we characterized the RNA-binding specificity of recombinantly expressed PvIESI-1 and PvIESI-2 domains in vitro using RNA-Bind-n-Seq (RBNS)^45^. In this approach, recombinant protein domains are incubated with 40-nucleotide random RNA library at varying protein concentrations. Protein-bound RNAs are then isolated and identified by next-generation sequencing revealing RNA sequences preferentially bound by each domain. We tested the recombinant RRM domain from IESI-1 and the 3× CCCH-type zinc finger domain from IESI-2. The top enriched sequences were determined by comparing their frequencies to those in a sequenced input library control^45^ across two replicates for each protein domain of interest (Fig. 7).

The PvIESI-1 RRM showed strongest enrichment for a 6-nucleotide RNA sequence 5’-UGAUGA-3’ across all tested protein concentrations (R-value average = 5.2 ± 0.6), with other highly enriched sequences containing the 5’-UGA-3’ core motif (Fig. 7A). To determine whether the high amino acid sequence conservation of RRM domains across *Plasmodium* species (Fig. S1C) corresponds to conserved RNA-binding specificity, we analyzed orthologous RRM domains from *Pf* (PF3D7_1139100) and *Py* (PY17X_0911200). Both orthologs displayed identical motif enrichment, with 5’-UGAUGA-3’ as the top sequence (Fig. 7A, Fig. S6A, B), and the enrichment patterns were highly correlated across all three species (Fig. 7B, Fig. S6C). These findings demonstrate that the PvIESI-1 RRM selectively binds RNA sequences containing the conserved 5’-UGA-3’ core motif, and this binding specificity is maintained across divergent *Plasmodium* species.

We next determined the RNA-binding specificity of PvIESI-2 3xCCCH-type zinc-finger domain using RBNS. The highest enrichment was observed for 9-nucleotide sequences, with 5’-GAUACCCGA-3’ showing the strongest enrichment (average R-value = 4.3 ± 0.2) (Fig. 7C). Analysis of top enriched sequences revealed a conserved consensus motif, 5’-UGAYAYYYG-3’ (preferring cytosines at Y positions) (Fig. 7C, D), with the 5’ portion showing the strongest enrichment across *Py* (PY17XNL_000504404) and *Pf* (PF3D7_1019300) (Fig. S6D, F; 5′-UGAYAC-3′). Within this motif, pyrimidine positions were predominantly occupied by cytidines, and inclusion of a preceding uracil defined the most conserved 6-nucleotide sequence, 5’-UGACAC-3’ (Fig. 7D). Since each individual CCCH-type zinc-finger domain typically recognizes tri-nucleotide sequences with varying affinities^46,47^, the 6-nucleotide 5’-UGACAC-3’ motif likely represents the optimal binding site that allows simultaneous engagement of two adjacent zinc finger domains, rather than requiring the full 9-mer sequence for high affinity binding. Together, these data demonstrate that PvIESI-1 and PvIESI-2 are bona-fide RNA-binding proteins with distinct and specific sequence preferences.

### PvIESI-1 and PvIESI-2 binding sequences are enriched within the *P. vivax* liver stage transcriptome

**Fig. 8:**
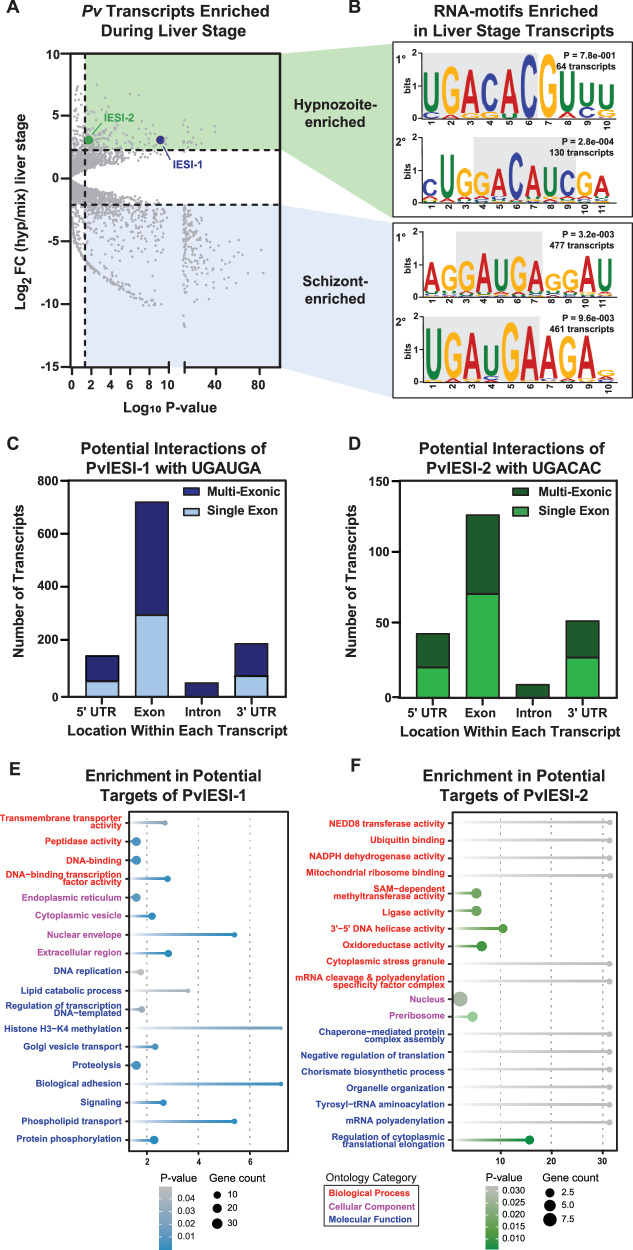
The IESI-1 and IESI-2 preferred binding sequences are relevant to *P. vivax* liver stage development. **A** The transcripts identified as differentially expressed in Gural et al.^17^ are visualized as a volcano plot showing their reported *P* values and Log_2_ Fold Change values, and **B** enriched motifs for each set were obtained using STREME with the *P. vivax* transcriptome as background. **C, D** The occurrences and locations (Exon, Intron, 5' or 3' Untranslated Regions (UTRs)) of the IESI-1 and IESI-2 binding sequences in the *P. vivax* transcriptome are indicated in stacked bar graphs. **E** Transcripts enriched in exo-erythrocytic schizont stages containing the IESI-1 preferred motif (5'-UGAUGA-3') and **F** hypnozoite transcripts containing the IESI-2 preferred motif were searched for enriched Gene Ontology terms (GO) using PlasmoDB (www.plasmodb.org) with *P* < 0.05. STREME and GO enrichment programs use Fisher’s Exact Tests to determine enrichment of either nucleotide motifs or specific processes above expected background values in **B**, **E**, **F**. Source data for this figure are provided in the Source Data file 2.

We next interrogated the *P. vivax* hypnozoite and liver-schizont transcriptomes to identify potential binding targets for PvIESI-1 and PvIESI-2, aiming to uncover their regulatory roles during liver stage development. Using an agnostic motif search across all liver stage-enriched transcripts, we assessed whether IESI-1 and IESI-2 preferred binding motifs identified by RBNS are present in developmentally regulated *Pv* liver stage transcripts. Differentially expressed transcripts from Gural et al.^17^ (Fig. 8A; Log_2_ Fold Change > 1, *P* < 0.05) were examined for motif enrichment of the IESI-1 and IESI-2 motifs in either hypnozoite- or schizont-enriched transcripts. Multiple motifs were identified as significantly enriched above background (E-value < 0.05, Supplementary data 2). In schizont-enriched transcripts, full (5’-UGAUGA-3’, *P* = 9.6 × 10^−3^) and partial (5’-GAUGA-3’, *P* = 3.2 × 10^−3^) matches to the IESI-1 motif appeared in 45% and 44% of transcripts, respectively (Fig. 8B). In hypnozoite-enriched transcripts, we identified both full (5’-UGACAC-3’, *P* = 7.8 × 10^−1^ and partial (5’-GACAUC-3’*, P* = 2.8 × 10^−4^) matches to the IESI-2 motif in 20% and 10% of transcripts, respectively (Fig. 8B).

When we examined all liver-schizont-enriched transcripts for the IESI-1 motif (Fig. 8A; 1059 transcripts), 79% contained at least one instance of the 5′-UGAUGA-3′ sequence. Because motif localization within transcripts can indicate functional significance, we examined the distribution of 5’-UGAUGA-3’ motifs, which were enriched in exonic regions, predominantly in multi-exonic transcripts (60%, Fig. 8C), suggesting a potential role in transcript splicing. The IESI-2 motif (5’-UGACAC-3’) was present in 33% of hypnozoite-enriched transcripts (Fig. 8A; 653 transcripts), primarily within coding sequences (Fig. 8D). Unlike IESI-1, the IESI-2 motif showed less exonic bias, and more frequently associated with introns or untranslated regions (UTRs) (Fig. 8D), suggesting a potential role in translational regulation of target transcripts.

We next performed a Gene Ontology (GO) enrichment analysis on transcripts containing each corresponding motif to identify potential functional categories of gene products that are co-regulated by PvIESI-1 or PvIESI-2. Transcripts containing IESI-1 motifs were strongly enriched for phosphate metabolism, particularly protein phosphorylation (GO:0006468) and phospholipid transport (GO:0015914) (Fig. 8E). Notably, 25 kinases and 6 phospholipid transporters contained the IESI-1 motif, suggesting IESI-1 might coordinate cellular energy management and signaling. These transcripts were also enriched for pathways essential to schizont development, including DNA replication (GO:0006260) and cell signaling (GO:0023052) (Fig. 8E). Among these are the most highly upregulated exported proteins identified in schizonts by Gural et al.^17^ (PVP01_0533700, PVP01_1402700, PVP01_0503800). Interestingly, IESI-1 motifs were also present in transcripts encoding DNA-binding transcription factors (GO:0006355), including ten ApiAP2 transcription factors such as AP2-I (PVP01_0807400) and AP2-LT (PVP01_0118100, Fig. 8E). This pattern suggests a regulatory hierarchy in which IESI-1 might act as a master regulator, potentially suppressing schizont-promoting factors to block schizogony.

GO enrichment analysis of hypnozoite-enriched transcripts containing IESI-2 motifs revealed potential links to dormancy-associated cellular machinery (Supplementary data 2). These putative target transcripts encompassed multiple aspects of gene expression regulation. First, they were involved in RNA metabolism: regulation of cytoplasmic translation elongation (GO:1900247), mRNA polyadenylation (GO:0006378), and cytoplasmic stress granules (GO:0010494). Second, they were enriched for negative regulation of translation (GO:0017148), including a 7-helix-1 protein (PVP01_1008400) and the known translational repressor PUF1 (PVP01_1015200). Third, transcripts encoding pre-ribosome components (GO:0030684) were enriched, such as BRX1 homolog (PVP01_0215100) and multiple RNA helicases (PVP0_1403600, PVP01_1408200). These findings implicate that PvIESI-2 may function as a key regulator of translational control, fine-tuning the gene expression machinery to promote blockade of schizogony.

## Discussion

When *P. vivax* sporozoites infect hepatocytes, they develop into trophozoites that either initiate immediate exo-erythrocytic schizogony, leading to primary blood stage infection, or form dormant hypnozoites, which persist in the liver and can activate after weeks or months to undergo secondary schizogony, which produces exo-erythrocytic merozoites long after primary infection and causes relapses^4,5^. Establishing this non-replicative state requires pausing cell cycle progression and preventing schizogony. We hypothesized that in hypnozoites, replication is actively suppressed through translational repression of genes necessary for schizogony^9^. Transcriptomic analyses of *Pv*^17–19^ and *P. cynomolgi*^20–22^ revealed distinct expression profiles between hypnozoites and schizonts, yet cross-study variability has hindered the identification of conserved regulators. To resolve this, we performed a comparative analysis and identified transcripts for two hypnozoite-enriched RNA-binding proteins, IESI-1 and IESI-2 and hypnozoite-specific expression was confirmed by gene-specific RNA-FISH.

The study of hypnozoite formation in *Pv* is limited by the absence of a continuous blood-stage culture and a facile transgenesis system, preventing generation of transgenic gametocytes, mosquito stages, and sporozoites^8,48^. Here, we used the *P. yoelii* model and its robust genetic tools to investigate *Pv* protein function. As *Py* lacks dormant liver stages, we hypothesized that expressing hypnozoite-associated genes could reveal latency mechanisms by showing novel phenotypes. Using UIS4 regulatory elements, PvIESI-1 *and* PvIESI-2 expression initiated post-hepatocyte infection, without affecting blood stage replication, gametocytogenesis, mosquito development, or sporozoite infectivity.

Hypnozoites are defined by their small cell size^8^, absence of DNA replication and lack of organelle expansion such as the endoplasmic reticulum, mitochondria, and apicoplast^7,17^, which clearly distinguishes them from replicating liver-stage schizonts. Overexpression of PvIESI-1 or PvIESI-2 in *Py* liver-stages recapitulated these features, producing static, uninuclear cells with underdeveloped organelles. Both transgenes delayed liver-to-blood stage transition compared to WT PyXNL, with PvIESI-2 showing a stronger inhibitory effect. This establishes PvIESI-1 and PvIESI-2 as potential regulators of developmental arrest and suggests they could be critical molecular switches controlling hypnozoite formation. Interestingly, a significant population of parasites with larger liver stage sizes for ^uis4^PvIESI-1^mNeon^ and ^uis4^PvIESI-2^mNeon^ parasites showed a diminished mNeon signals (Fig. S4C, D), indicating declining PvIESI expression. An important aspect of hypnozoite biology is reversibility of the non-replicative state (activation)^7,49^, which allows hypnozoites to enter schizogony. However, whether the declining expression of PvIESI-1 and PvIESI-2 in parasites is mechanistically linked to entry into schizogony cannot be ascertained from these observations and requires further study.

Translational repression mediated by cytoplasmic granules is a well-established regulatory mechanism in *Plasmodium* transmission stages. In gametocytes, maternal mRNAs are sequestered within DOZI- and CITH-containing messenger ribonucleoprotein (mRNP) complexes to ensure timely expression after fertilization^36,50,51^. Similarly, in sporozoites, RNA-binding proteins such as PUF2 and ALBA family members suppress translation to maintain infectivity until host invasion^35,44^. These stress granule–like assemblies underscore an evolutionarily conserved post-transcriptional control mechanism that ensures precise temporal protein expression just-in-time during host transitions. PvIESI-1 and PvIESI-2 were continuously expressed between 6 and 44 h after sporozoite infection and primarily localized to cytoplasmic granules. Their co-localization with the stress granule-associated protein PABP1^38,39^, indicated a role in RNA regulatory complexes, potentially involved in translational repression. These features align with their identity as RNA-binding proteins and point to a possible function in modulating mRNA fate during liver stage development. By influencing mRNA stability or translation, PvIESI-1 and PvIESI-2 might contribute to hypnozoite formation, suggesting them as potential targets for disrupting *Pv* persistence. However, since these expression and localization profiles originate from an artificial overexpression system, it will be important to further investigate their endogenous expression and localization using *Pv* hepatocyte infection models.

Given their annotation as RNA-binding proteins and their strong effects on liver stage development, we experimentally verified PvIESI-1 and PvIESI-2 as bona fide RNA-binding proteins. Using RNA Bind-n-Seq (RBNS), we identified distinct sequence motifs bound by the RNA-binding domains of IESI-1 and IESI-2 orthologs across three *Plasmodium* species. Although this in vitro approach cannot fully capture the spectrum of IESI-bound transcripts like in vivo approaches such as cross-linking immunoprecipitation and sequencing (CLIP-seq), it directly confirms specific RNA-binding activity to conserved motifs^45^ in systems such as *Pv* where generating samples for in vivo approaches may be untenable. The high conservation and sequence specificity of the IESI RNA-binding domains among *P. vivax*, *P. yoelii*, and *P. falciparum* underscore their essential molecular function.

The IESI-1 binding motif appeared across a broader mRNA target repertoire in liver stage parasites, suggesting modulatory role in stress adaptation or developmental plasticity rather than direct dormancy enforcement. PvIESI-1, annotated as a paralog of CELF1 (PVP01_1112500) and showing the phylogenetic similarity to splicing factor 3B, subunit 4 (PTHR15241: SF62, E-value = 1 × 10^−36^), may participate in RNA processing. Its *P. cynomolgi* ortholog (PCYB_094480, 74% identity) is predicted to positively regulate mRNA splicing via the spliceosome (GO:0048026). Consistent with these predictions, the human CELF1 binding motif (5’-UGU-3’)^45^ resembles the 5’-UGA-3’ sequence enriched in IESI-1 targets, supporting a possible role for IESI-1 in RNA splicing or processing. The IESI-1 motif is enriched in multi-exonic, highly expressed schizont-stage transcripts associated with DNA metabolism, helicase activity, and transcriptional regulation. The precise nature of regulation by PvIESI-1 remains uncertain, as CELF family splicing factors can function as either activators or repressors depending on their interaction partners in vivo^52^.

PvIESI-2 is annotated as a paralog of PVP01_0725700, the syntenic ortholog of gametocyte development protein 1 (GD1) in *P. berghei*, a gene essential for the production of gametocytes in rodent malaria species^53^. Although the function of both paralogs is still unexplored in *Pv*, the PvIESI-2 binding motif was enriched in hypnozoite-associated transcripts, and its perturbation strongly inhibits schizogony. These findings suggests that PvIESI-2 might function as a key effector of dormancy, possibly through the activation of hypnozoite-related transcripts, while PvIESI-1 provides a broader regulatory framework that supports stage transitions. Together, PvIESI-1 and PvIESI-2 might mediate post-transcriptional programs that coordinate the balance between dormancy and replication in *Pv* liver-stage development. Although in vitro RBNS identified specific RNA motifs bound by PvIESI-1 and PvIESI-2, in vivo validation through CLIP or RIP (RNA immunoprecipitation) would help identify their direct mRNA targets. While such experiments remain challenging in *Pv*, they are feasible in genetically tractable models such as *Pf* and *Py* whose conserved IESI RNA-binding domains share nearly identical RNA-binding motifs.

The RNA-binding domains of IESI-1 and IESI-2 are highly conserved across *Plasmodium* species, suggesting a shared molecular function in RNA recognition. However, despite this conservation, the *Pv* proteins might uniquely suppress schizogony during liver stage development, whereas the *Pf* and *Py* orthologs might not exhibit this phenotype under natural conditions. This functional divergence implies that regions outside the conserved RNA-binding domains might play a crucial role in determining species-specific regulatory functions. Indeed, sequence comparisons reveal substantial divergence in the non-RNA-binding regions among *Plasmodium* species, which might modulate the protein-protein interactions, subcellular localization, or post-translational modifications, ultimately shaping their roles in liver-stage development and dormancy regulation.

The comparison of IESI-1 and IESI-2 full-length protein sequences indicated that the *Pv* orthologs are genetically closer to *P. cynomolgi* than to orthologs in other *Plasmodium* species. This genetic proximity is markedly distinct from the human parasite *P. ovale*, which also forms hypnozoites^54,55^. If distinct biological functions reside in IESI-1 and IESI-2 protein regions unique to *Pv* and *P. cynomolgi*, the molecular mechanisms or conserved pathways regulating hypnozoite dormancy are more likely shared between *Pv* and *P. cynomolgi* rather than between *Pv* and *P. ovale*. Future studies examining the structural similarities and differences of IESI-1 and IESI-2 between relapsing and non-relapsing parasites might identify protein regions that mediate species-specific functions and reveal adaptations associated with hypnozoite formation and this will help to refine structure/function analysis of these proteins. If functional differences reside within the protein sequences, future overexpression studies of IESI-1 and IESI-2 orthologs from non-relapsing parasites, such as *Pf*, in *Py* and the design of chimeric proteins might reveal protein regions that are specifically involved in inducing a non-replicative state in liver stages.

Differences in expression profiles might also contribute to the distinct phenotypic effects of these proteins across parasite life cycle stages. Transcriptomic datasets available for PvIESI-1 and 2 showed preferential expression of either proteins in hypnozoites compared to liver schizonts but there was also evidence for blood stage expression of PvIESI-1 and 2 in patient isolates^56^. In *Py* and *Pf*, many more transcriptomic datasets are available for comparison of expression across the life cycle. In *Py*, IESI-2 transcripts were expressed at higher levels in blood stages than in salivary gland sporozoites (Fig. S7A), while IESI-1 displayed a similar expression pattern but with significantly lower transcript levels (Fig. S7A). This pattern is consistent with previously published microarray datasets^31^, which similarly show that PyIESI-2 transcripts are predominantly expressed during blood stage development, with minimal expression detected in sporozoite and liver stages (Fig. S7B), whereas IESI-1 consistently exhibits negligible transcript levels throughout the parasite life cycle (Fig. S7B). In *Pf*, PfIESI-1 transcripts were expressed most highly in salivary gland sporozoites^57^, though the transcript is also well-detected in ring and ookinete stages^58^ (Fig. S7C). Conversely, the PfIESI-2 transcript was most highly expressed in oocyst stages^57^ but was also well-detected in late-stage gametocytes and ookinetes^59^ (Fig. S7D).

These different expression patterns suggest that while these orthologous proteins bind the same RNA motifs cross *Plasmodium* species, their regulatory control has diverged. Relapsing species may have evolved altered expression of genes controlling parasite replication, potentially enabling these proteins to function in hypnozoite formation and maintenance. Future studies will characterize the endogenous expression and subcellular localization of these proteins in *Py, Pb*, and *Pf*, assessing whether translational regulation is conserved or has diverged among species with different liver stage developmental strategies.

Our data reveal important functions of PvIESI-1 and PvIESI-2 using the *Py* model. However the formation and persistence of hypnozoite in *Pv* are likely more complex than regulation by PvIESI-1 or PvIESI-2 alone. Many more factors must be involved, particularly those that enable hypnozoites to survive within hepatocytes and factors that enable activation. The fact that ^uis4^PvIESI-1^mNeon^ or ^uis4^PvIESI-2^mNeon^ *Py* parasites do not persist very well suggests that this species might lack key survival mechanisms of parasite forms that show prolonged growth and replication-quiescence in the liver.

**Fig. 9:**
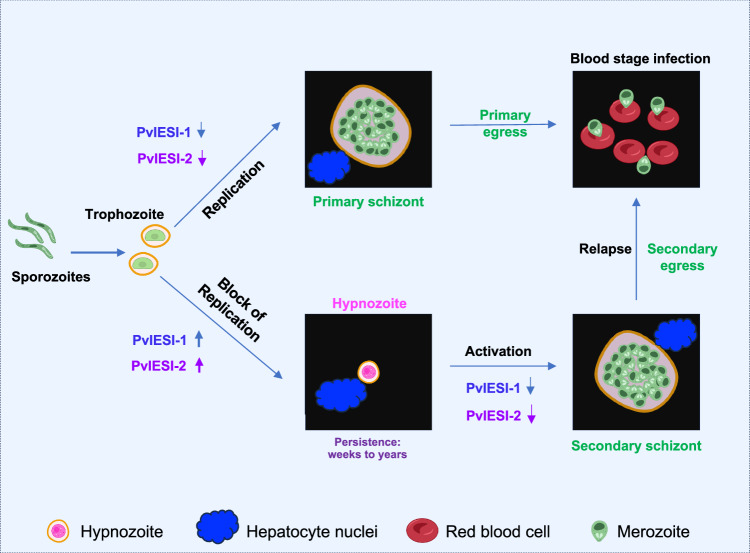
Proposed model for PvIESI-1 and PvIESI-2 – mediated regulation of liver stage fate. Schematic model illustrating how the formation of either replicating schizonts or dormant hypnozoites might depend on the expression levels of PvIESI-1 and PvIESI-2. The schematic diagram was generated using Microsoft PowerPoint, and the editable source file (in ppt format) is provided within this published article.

In summary, our study demonstrates that PvIESI-1 and PvIESI-2 are important suppressors of parasite replication, likely via modulation of post-transcriptional repression of transcripts that encode proteins required for schizogony. This suggests a working model in which elevated expression and RNA-binding activity of PvIESI-1 and PvIESI-2 promotes hypnozoite formation. Conversely, reduced expression might relieve this repression, allowing parasites to exit dormancy and enter schizogony. In established hypnozoites, subsequent changes in PvIESI-1 and PvIESI-2 abundance could be associated with activation, leading to secondary schizogony and potential blood-stage relapse (as illustrated in Fig. 9).

A key limitation of this study is that our functional characterization of IESI-1 and 2 relied on overexpression in a rodent malaria model, which cannot fully capture the biology of *Pv* liver stages, particularly hypnozoite persistence, and activation. However, given the current technical barriers to continuous culture and genetic manipulation of *Pv*, the experimental model we used provides a clear and tractable framework for functional interrogation of proteins that might regulate hypnozoite biology. Accordingly, our conclusions are constrained by the model used and should not be considered definitive for relapsing parasites. Despite these limitations, our findings provide a potential key mechanism by which malaria parasites can enter a non-replicating state in the liver and informs our understanding of dormancy in *Pv*. Integration of our functional data with RBNS-derived RNA targets and published liver stage transcriptomes revealed consistent enrichment of genes involved in translational control, cellular quiescence, and early liver stage development. This convergence supports a model in which IESI-1 and IESI-2 regulate post-transcriptional networks linked to parasite dormancy and provides a framework for future validation in relapsing malaria parasites.

## Methods

### Experimental animals and eukaryote cell lines

Seven-to eight-week-old female Swiss Webster (SW) mice purchasing from Envigo were used for the maintenance of *Py* life cycle and generation of transgenic parasites. The human hepatocyte HepG2-CD81 cells (originally obtained from Olivier Silvie Laboratory, Cimi, Paris, France) were cultured and maintained in DMEM medium supplemented with FBS, GlutaMAX and pen/strep antibiotics. HepG2-CD81 cells were used to infect with sporozoite of the PyXNL and transgenic strains. *Anopheles Stephensi* mosquitoes were reared in the in-house Insectary. Primary Human Hepatocytes (PHH) were purchased directly from BioIVT (BioIVT, batch #BGW, derived from a female donor). The PHH were used to infect *P. vivax* sporozoites. Four to six days old female mosquitoes were used to run the Plasmodium parasites’ infection cycle.

### Ethical oversight

This study was carried out in accordance with the recommendations of the NIH Office of Laboratory Animal Welfare standards (OLAW welfare assurance #D16-00119). The mice were maintained under specific pathogen-free conditions with 12 h light/12 h dark cycle, 72 °F temperature and 45% humidity at the Center for Global Infectious Disease Research, Seattle Children’s Research Institute (SCRI). The protocols were approved by the Center for Infectious Disease Research Institutional Animal Care and Use Committee (IACUC) under Protocol SK00505 and SK00666 (for rodent malaria parasites).

### Plasmid construction

The vector containing 5’ and 3’flanking regions of PyP230P was generated using the pYC vector backbond^60^. A single guide RNA (sgRNA; sequence: GAATGTGGTGTAACAAATGG) was designed to specifically target the PyP230P locus. The uis4 promoter and its corresponding 3′ regulatory/terminator region were subsequently inserted into the vector using AscI/PmeI and NheI/SalI restriction sites, respectively.

Fluorescent reporter cassettes were then generated by ligating either the mNeon or mCherry coding sequences into the vector via KpnI/NheI restriction sites, resulting in the pUIS4-mNeon (Figure S3B) and pUIS4-mCherry constructs. Codon-optimized open reading frames (ORFs) of PvIESI-1 and PvIESI-2 (synthesized by GENEWIZ and GenScript, respectively) were subsequently cloned into both fluorescent and non-fluorescent vectors at the PmeI/KpnI restriction sites. This cloning strategy generated four final constructs: pUIS4-PvIESI-1-mNeon and pUIS4-PvIESI-2-mNeon (Fig. S2A), as well as pUIS4-PvIESI-1 and pUIS4-PvIESI-2 (Fig. S3A).

In parallel, a codon-optimized PvPUF2 ORF (synthesized by GENEWIZ) was inserted into the pUIS4-mCherry vector using the same cloning strategy to generate the pUIS4-PvPUF2-mCherry construct (Fig. S5B).

### Creation of *P. yoelii* expressing PvIESI-1 and PvIESI-2 ORFs, and PvPUF2 ORF

The transfection and clonal isolation procedures were performed based on previously described protocols^61,62^ with several modifications to optimize efficiency for the PyXNL strain. Briefly, plasmid constructs were purified using a QIAGEN Plasmid Midi Kits (Cat no./ ID 12143), and quantified prior to transfection. For each transfection, 10 µg of plasmid DNA was resuspended in 100 µL of Human T Cell Nucleofector Solution (Lonza; Catalog #VPA-1002).

Highly synchronized blood-stage schizonts of PyXNL parasites were enriched by density gradient centrifugation using 60% Accudenz. Purified schizonts were washed once in incomplete RPMI and resuspended in nucleofection solution containing plasmid DNA. Electroporation was performed using an Amaxa Nucleofector II device (Lonza) with program U-033. Transfected parasites were immediately recovered in pre-warmed complete RPMI prior to injection.

For in vivo propagation, the entire transfection mixture was injected retro-orbitally into Swiss Webster mice under anesthesia. Parasitemia was monitored daily by Giemsa-stained thin blood smears. Drug selection was initiated 24 h post-injection with pyrimethamine (mg/L) administered in the drinking water and maintained until resistant parasites emerged (typically 3–5 days).

To obtain clonal parasite lines, limiting dilution cloning was performed. Briefly, infected blood was diluted serially in naïve erythrocytes to achieve approximately three parasites per inoculum, and retro-orbital injected into individual recipient mice. Clonal populations were confirmed by monitoring parasitemia and, where necessary, by genotyping.

Using a CRISPR/Cas9-based genome editing strategy, we successfully generated six independent transgenic parasite lines: ^uis4^PvIESI-1^mNeon^, ^uis4^PvIESI-2^mNeon^, ^uis4^PvIESI-1, ^uis4^PvIESI-2, ^uis4^mNeon, and ^uis4^PvPUF2^mCherry^. Integration of the transgenes was confirmed by diagnostic PCR using locus-specific primers, and, where applicable, by fluorescence microscopy to verify reporter expression.

All oligonucleotide primers used for the generation and genomic screening of ^uis4^PvIESI-1^mNeon^, ^uis4^PvIESI-2^mNeon^, ^uis4^PvIESI-1, ^uis4^PvIESI-2, ^uis4^mNeon line, and ^uis4^PvPUF2^mCherry^ are listed in Table S2. Table S2 is provided in Supplementary Information file with this manuscript. All primers were obtained from Integrated DNA Technologies (IDT) vendor.

### In vivo and in vitro sporozoites infection

The *A. stephensi* mosquito’s salivary gland infected with seven parasite lines (PyXNL, ^uis4^PvIESI-1^mNeon^, ^uis4^PvIESI-2^mNeon^, ^uis4^PvIESI-1, ^uis4^PvIESI-2, ^uis4^mNeon, and ^uis4^PvPUF2^mCherry^), sporozoites were dissected between 14 and 15 days after the primary infectious blood meal. The alive-inactive sporozoites in ice-Schneider buffer were retro-orbital injected into SW mice (10,000 Spz/mouse, for patent examination). To set up the invitro infection, 5 × 10^4^–7.5 × 10^4^ Spz were seeded into the confluent HepG2-CD81 cells, and sampled at 6, 12, 24, and 44 h following infection.

### RNA-FISH assay

RNA fluorescence in situ hybridization (RNA-FISH) combined with immunofluorescence assay (IFA) was performed on *P. vivax* liver-stage schizonts and hypnozoites derived from infected primary human hepatocytes (BioIVT, batch #BGW) at day 8 post-infection. The RNAscope Multiplex Fluorescent Assay v2 (Advanced Cell Diagnostics) was carried out according to the manufacturer’s instructions. Cells were treated with protease Solution 3 (1:10 dilution) for 20 min at room temperature, followed by overnight incubation with mouse monoclonal anti-PvUIS4 antibody (1:1000 dilution)^63^, and fixed in neutral-buffered formalin for 10 min.

Probe hybridization was performed for 2 h at 40 °C using probes targeting *P. vivax* transcripts PVP01_0939900 (PvIESI-1) (20ZZ probe, regions 2–1056) and PVP01_0604500 (PvIESI-2) (20ZZ probe, regions 2702–3701), PVP01_0526500 (PvPUF2) (20ZZ probe, regions 200-1232) and PVP01_0602100 (PvUIS4) (9ZZ probe, regions 2–458). Signal amplification was achieved using tyramide signal amplification (TSA) with TSA Vivid fluorophores, following the manufacturer’s protocol. Cells were counterstained with Alexa Fluor 488-conjugated secondary antibody and DAPI. Imaging was performed using the Stellaris 8 confocal microscope (Leica Microsystems), with image processing via Leica Lightning and analysis conducted in Fiji (ImageJ).

### RT-qPCR

1 × 10^6^ to 2 × 10^6^ SG Spz for each parasite strain was used to extract the total RNA, using Qiagen miRNeasy mini kit (cat. no. 217004). QuantiTect-Qiagen Reverse transcription kit (Cat. No. 205311) was used to reverse RNA to cDNA. qRT-PCR was performed using SYBR Green Master Mix (cat. no. B21202) and Reference Dye 2 on a Real-time PCR instrument (QuantStudio 5). Specific primers for the target genes, and *Py*18S RNA gene were designed using PrimerQuest^TM^ Tool (https://www.idtdna.com/PrimerQuest/Home/Index). The primer sequences are detailed in Table S2. Relative gene expression was calculated using the 2^−ΔΔCt^ method, with normalization to the control Py18S RNA gene. All reactions were performed in triplicate.

### Indirect immunofluorescence assay (IFA)

IFA was performed as previously described^64^. Briefly, either salivary gland sporozoites (SG Spz) or SG-infected HepG2 + CD81 cells (at 6, 12, 24, or 44 hpi) were fixed with 4% PFA in PBS for 10–15 min, followed by three 5-min washes with PBS. For SG Spz, fixed parasites were air-dried in a biosafety hood overnight before proceeding to immunostaining.

Dry-fixed sporozoites and fixed SG-infected HepG2-CD81 cells were blocked and permeabilized with 0.2% Triton X-100/2% BSA in PBS for 20 min at room temperature (RT). Primary antibodies, diluted in 0.2% Triton X-100/2% BSA/PBS, were applied for 1–2 h at RT or overnight at 4 °C. Samples were washed three times with PBS, then incubated with secondary antibodies for 45 min at RT. After one PBS wash, cells were stained with DAPI for 15 min at RT, followed by three PBS washes and mounting with antifade reagent. Samples were air-dried overnight before imaging.

The primary antibodies used including, PymTIP (1:500, Stefan Kappe, rabbit polyclonal)^65^, PbUIS4 (1:500, goat polyclonal AB0042-500, SIGEN), mNeonGreen (1:400, mouse monoclonal 32F6, Proteintech), mCherry (1:400, rat monoclonal antibody (16D7), Invitrogen), Histon H3K9Ac (1:200, mouse monoclonal MABI0305, Gene Tex), PvHSP70 (1:400, rabbit, polyclonal)^7^, PyPABP1 (1:200, Scott Lindner, rabbit polyclonal)^38^. Secondary antibodies were used including, donkey anti-mouse 488 (Invitrogen, Cat # A21202; RRID: AB_141607), donkey anti- mouse 594 (Invitrogen, Cat # A21203; RRID: AB_141633), donkey anti-rabbit 488 (Invitrogen, Cat # A-21206; RRID: AB_2532792), donkey anti-rabbit 594 (Invitrogen, Cat # A21207; RRID: AB_141637), donkey anti-rabbit 647 (Invitrogen, Cat # A-31573; RRID: AB_2536183), donkey anti-goat 594 (Invitrogen, Cat # A-11058; RRID: AB_2534105), donkey anti-goat 647 (Invitrogen, Cat # A-21447; RRID: AB_2535864), donkey anti-rat 594 (Invitrogen, Cat # A-21209; RRID: AB_2535795). All the secondary antibodies were used at a dilution 1:1000.

### Microscopy

All fluorescent images were captured using the Stellaris 8 confocal microscopy (Leica Microsystems), equipped with 63× water objective. The Lightning software (Leica Microsystems) and LAS X Life Science Microscope Software were used for image processing. The liver stage counts and sizes were quantified using the “Hybrid Duo Count and Measure” function in Keyence Microscopy (BZ-X710).

### RNA Bind-n-Seq experiments

For IESI-1, gene sequences that correspond to homologous regions containing the annotated RRM for orthologues from *P. falciparum* (aa 875–1232), *P. vivax* (aa 896–1203) and *P. yoelii* (aa 795–956) were cloned into the pGEX6P1 (Cytiva, cat. no. 28-9546-48) vector for expression with an N-terminal GST-tag. Sequences corresponding to aa 1–109 containing the three CCCH zinc finger domains of IESI-2 orthologues from *P. falciparum, P. vivax* and *P. yoelii* were also cloned downstream of a GST-tag in the pGEX6P1 (Cytiva, cat. no. 28-9546-48) vector for recombinant protein expression in bacteria, *E. coli* Rosetta 2 (DE3)pLysS (EMD Millipore, cat. no. 71401). To prepare for protein expression, bacteria were grown from a starter culture in 100 mL LB broth with 100 µg/mL ampicillin and 35 µg/mL chloramphenicol at 37 °C until OD_600_ of approximately 0.6 before induction with 0.2 mM IPTG. Following induction, the temperature was reduced to 30 °C and 0.1 mM zinc chloride was added to bacterial cultures expressing IESI-2 and protein was expressed for 2 h before freezing the bacterial pellet at −80 °C until purification. Prior to purification, bacteria were lysed in BPER reagent (Thermo Fisher Scientific, cat. no. 89821) at room temperature for 10 min. Purification of the GST-tagged proteins was performed using a GST Fusion Protein purification kit (Genscript, cat. no. L00207) per the manufacturer’s instruction with modifications. The GST-tagged protein purification included the addition of 10 U of DNAse (Biobasic, cat. no. BS88253) and 10 U RNAse A (Biobasic, cat. no. RB0473) to the bacterial lysate. For IESI-2, the pH of the gravity flow buffer was decreased to 6.8 to prevent chelation of the zinc from the CCCH zinc finger in the proteins^66^ before elution at pH 8.0 in the standard elution buffer recommended by the manufacturer. RNA bind-n-seq (RBNS) was carried out at room temperature as in^45^, using 50, 100, and 500 nM concentrations of the IESI-1 RRM or IESI-2 zinc finger domains from each species and a 40-mer random in vitro transcribed RNA library. The GST-protein-RNA complexes were pulled down using glutathione high-capacity magnetic agarose beads (Sigma Aldrich, cat. no. G0924) prior to three washes in wash buffer (25 mM Tris, pH 7.5, 150 mM KCl, 60 μg/mL BSA, 0.5 mM EDTA, 0.01% Tween). The RNA was released from the protein complex in elution buffer (10 mM Tris, pH 7.0, 1 mM EDTA, 1%SDS) for 10 min at 70 °C and remaining protein was digested with 1.5 µg proteinase K for 15 min at 37 °C. The RNA was then purified on Zymo RNA clean and concentrator columns (cat. no. R1016) and eluted into nuclease-free water. The purified RNA and 0.5 pmol of the input library were reverse transcribed using SSIV (Invitrogen, cat. no. 18090010) and sequencing library preparation via PCR with primers and indexes for Illumina Truseq RNA libraries as described in Lambert et al.^67^. RBNS libraries were pooled and single-end sequencing performed with a Miseq V3 at a concentration of 10 pM. Data from RBNS were analyzed in Linux using scripts from the C Burge lab RBNS pipeline to identify sequences enriched the bound RNA (https://github.com/cburgelab/RBNS_pipeline). Representative sequence logos of motifs enriched in RNA-bind-n-seq were generated in seqlogo (10.18129/B9.bioc.seqLogo) in R v 4.2.3 and graphs were created in GraphPad Prism v 10.

### Sequence analyses

Motif searches:

Enriched sequence motifs were identified hypnozoite associated transcripts by using data from Gural et al.^17^. Transcripts that were increased > 1 Log_2_ Fold Change, *P* < 0.05 in hypnozoites or liver stage parasites were searched for enriched sequenced motifs compared to all *P. vivax* transcripts as background using STREME. To identify occurrences of the RBNS motif in transcripts, a.fasta file containing all *P. vivax* transcribed sequences was searched using SeqKit^68^ v2.10.0 for occurrences of either 5’-TGATGA-3’ or 5’-TGACAC-3’ and the location mapped back to the annotated gene components (intron, exon, UTRs) in the.gtf file for *P. vivax*.

Protein structures:

The predicted protein structures of the RRM domain (1043 aa–1123 aa) in PVP01_0939900 and C3H1-type domains (1–100 aa) in PVP01_0604500 were generated using ColabFold^69^ v1.5.5 with default parameters. From ColabFold v1.5.5, the best ranked model was selected and visualized with ChimeraX^70^ v1.9. Sequence conservation was assessed by aligning the corresponding residues in *P. yoelii* of the RRM domain in PY17X_0911200 and CH31-type domains in Py17XNL_000504404.

### Phylogeny

Sequences were retrieved from BLASTP of PVP01_0939900 (IESI-1) & PVP01_0604500 (IESI-2). Sequences were aligned with MAFFT^71^ v7.525 (default parameters) and trimmed with ClipKIT^72^ v2.1.3 using the “smart-gap” mode. The trimmed sequences were then used to construct a maximum likelihood tree (ML) with IQtree^73,74^ v3.0.1. with 1000 ultrafast bootstrap replicates and the best fit model for IESI-1 (Q.BIRD + F + I + G4) and IESI-2 (Q.MAMMAL + F + I + G4). The *Plasmodium* species, *P. gallinaceum* and *P. relictum* were used as outgroups to root both trees and each tree were visualized and exported from ITOL^75^.

### Statistics and reproducibility

The experiments were performed using the number of mice between 2 and 3 per group. At least three biological replicates of *P. yoelii* SG Spz from each of the seven parasite lines were used to infect HepG2-CD81 cells. Additionally, a minimum of three biological replicates per liver-stage time point (6, 12, 24, and 44 hpi) were included to enable robust quantification of liver-stage parasite size and number. The presented data are either from there to five independent experiments. All attempts at replication in this study were successful. Immunofluorescence assay (IFA) images with marker staining yielded consistent results across all independent experiments. The statistical analyses were performed in GraphPad Prism v10.3.0. Two-sided non-parametric Mann–Whitney U-test was used for comparing two groups. Two-way ANOVA with Tukey’s multiple comparison test for comparing three groups. A *P* value of <0.05 was considered as statistically significant.

### Reporting summary

Further information on research design is available in the Nature Portfolio Reporting Summary linked to this article.

## Supplementary information

Supplementary Information

Description Of Additional Supplementary File

Supplementary Data 1

Supplementary Data 2

Reporting summary

Transparent Peer Review file

## Source data

Source Data

## Data Availability

For the RNA-bind-n-seq results, raw sequencing reads have been submitted to the SRA: PRJNA1372697 [https://www.ncbi.nlm.nih.gov/bioproject/?term = (PRJNA1372697)] with public access and the processed data are included in Supplementary data 1. The Figure and Supplementary Fig. data generated in this study are provided in the Source Data files. Source Data files are provided with this paper.

## References

[1] World Health Organization. *World Malaria Report 2025*. (World Health Organization, Geneva, 2025).

[2] Howes, R. E. et al. Global epidemiology of Plasmodium vivax. *Am. J. Trop. Med Hyg.* **95**, 15–34 (2016). 27402513 10.4269/ajtmh.16-0141 PMC5198891

[3] Vaughan, A. M. & Kappe, S. H. I. Malaria parasite liver infection and exoerythrocytic biology. *Cold Spring Harb. Perspect. Med.* **7**, a025486 10.1101/cshperspect.a025486 (2017). 10.1101/cshperspect.a025486 PMC5453383 28242785

[4] Flannery, E. L. et al. Plasmodium vivax latent liver infection is characterized by persistent hypnozoites, hypnozoite-derived schizonts, and time-dependent efficacy of primaquine. *Mol. Ther. Methods Clin. Dev.* **26**, 427–440 (2022). 36092359 10.1016/j.omtm.2022.07.016 PMC9418049

[5] Zanghi, G. & Vaughan, A. M. Plasmodium vivax pre-erythrocytic stages and the latent hypnozoite. *Parasitol. Int* **85**, 102447 (2021). 34474178 10.1016/j.parint.2021.102447

[6] Krotoski, W. A. et al. Demonstration of hypnozoites in sporozoite-transmitted Plasmodium vivax infection. *Am. J. Trop. Med. Hyg.* **31**, 1291–1293 (1982). 6816080 10.4269/ajtmh.1982.31.1291

[7] Mikolajczak, S. A. et al. Plasmodium vivax liver stage development and hypnozoite persistence in human liver-chimeric mice. *Cell Host Microbe*. **17**, 526–535 (2015). 25800544 10.1016/j.chom.2015.02.011 PMC5299596

[8] Adams, J. H. & Mueller, I. The biology of Plasmodium vivax. *Cold Spring Harb. Perspect Med.* **7**, a025585 10.1101/cshperspect.a025585 (2017). 10.1101/cshperspect.a025585 PMC5580510 28490540

[9] Schafer, C., Zanghi, G., Vaughan, A. M. & Kappe, S. H. I. Plasmodium vivax latent liver stage infection and relapse: biological insights and new experimental tools. *Annu. Rev. Microbiol.* **75**, 87–106 (2021). 34196569 10.1146/annurev-micro-032421-061155

[10] Frampton, J. E. Tafenoquine: first global approval. *Drugs* **78**, 1517–1523 (2018). 30229442 10.1007/s40265-018-0979-2

[11] Bennett, J. W. et al. Primaquine failure and cytochrome P-450 2D6 in Plasmodium vivax malaria. *N. Engl. J. Med*. **369**, 1381–1382 (2013). 24088113 10.1056/NEJMc1301936

[12] Krotoski, W. A. et al. Observations on early and late post-sporozoite tissue stages in primate malaria. I. Discovery of a new latent form of Plasmodium cynomolgi (the hypnozoite), and failure to detect hepatic forms within the first 24 hours after infection. *Am. J. Trop. Med. Hyg.* **31**, 24–35 (1982). 7058977

[13] Krotoski, W. A. et al. Relapses in primate malaria: discovery of two populations of exoerythrocytic stages. Preliminary note. *Br. Med J.* **280**, 153–154 (1980). 6766771 10.1136/bmj.280.6208.153-a PMC1600318

[14] Schafer, C. et al. A Humanized mouse model for Plasmodium vivax to test interventions that block liver stage to blood stage transition and blood stage infection. *iScience* **23**, 101381 (2020). 32739836 10.1016/j.isci.2020.101381 PMC7399188

[15] March, S. et al. A microscale human liver platform that supports the hepatic stages of Plasmodium falciparum and vivax. *Cell Host Microbe*. **14**, 104–115 (2013). 23870318 10.1016/j.chom.2013.06.005 PMC3780791

[16] Mazier, D. et al. Cultivation of the liver forms of Plasmodium vivax in human hepatocytes. *Nature* **307**, 367–369 (1984). 6363939 10.1038/307367a0

[17] Gural, N. et al. In Vitro culture, drug sensitivity, and transcriptome of Plasmodium vivax hypnozoites. *Cell Host Microbe*. **23**, 395–406 e394 (2018). 29478773 10.1016/j.chom.2018.01.002 PMC8048090

[18] Mancio-Silva, L. et al. A single-cell liver atlas of Plasmodium vivax infection. *Cell Host Microbe*. **30**, 1048–1060 e1045 (2022). 35443155 10.1016/j.chom.2022.03.034 PMC12285726

[19] Ruberto, A. A. et al. Single-cell RNA profiling of Plasmodium vivax-infected hepatocytes reveals parasite- and host- specific transcriptomic signatures and therapeutic targets. *Front Cell Infect. Microbiol.* **12**, 986314 (2022). 36093191 10.3389/fcimb.2022.986314 PMC9453201

[20] Cubi, R. et al. Laser capture microdissection enables transcriptomic analysis of dividing and quiescent liver stages of Plasmodium relapsing species. *Cell Microbiol.* **19**, e12735 (2017). 10.1111/cmi.12735 PMC5516136 28256794

[21] Voorberg-van der Wel, A. et al. A comparative transcriptomic analysis of replicating and dormant liver stages of the relapsing malaria parasite *Plasmodium cynomolgi*. *eLife* **6**, e29605 (2017). 10.7554/eLife.29605 PMC5758109 29215331

[22] Toenhake, C. G. et al. Epigenetically regulated RNA-binding proteins signify malaria hypnozoite dormancy. *Cell Rep.* **42**, 112727 (2023). 37392389 10.1016/j.celrep.2023.112727

[23] Reddy, B. P. et al. A bioinformatic survey of RNA-binding proteins in Plasmodium. *BMC Genomics* **16**, 890 (2015). 26525978 10.1186/s12864-015-2092-1 PMC4630921

[24] Zhang, M. et al. The Plasmodium eukaryotic initiation factor-2alpha kinase IK2 controls the latency of sporozoites in the mosquito salivary glands. *J. Exp. Med.* **207**, 1465–1474 (2010). 20584882 10.1084/jem.20091975 PMC2901070

[25] Silvie, O., Goetz, K. & Matuschewski, K. A sporozoite asparagine-rich protein controls initiation of Plasmodium liver stage development. *PLoS Pathog.* **4**, e1000086 (2008). 18551171 10.1371/journal.ppat.1000086 PMC2398788

[26] Aly, A. S. et al. Targeted deletion of SAP1 abolishes the expression of infectivity factors necessary for successful malaria parasite liver infection. *Mol. Microbiol.* **69**, 152–163 (2008). 18466298 10.1111/j.1365-2958.2008.06271.x PMC2615191

[27] Kaiser, K., Matuschewski, K., Camargo, N., Ross, J. & Kappe, S. H. Differential transcriptome profiling identifies Plasmodium genes encoding pre-erythrocytic stage-specific proteins. *Mol. Microbiol.* **51**, 1221–1232 (2004). 14982620 10.1046/j.1365-2958.2003.03909.x

[28] Mueller, A. K. et al. Plasmodium liver stage developmental arrest by depletion of a protein at the parasite-host interface. *Proc. Natl. Acad. Sci. USA.* **102**, 3022–3027 (2005). 15699336 10.1073/pnas.0408442102 PMC548321

[29] Mackellar, D. C. et al. Plasmodium falciparum PF10_0164 (ETRAMP10.3) is an essential parasitophorous vacuole and exported protein in blood stages. *Eukaryot. Cell* **9**, 784–794 (2010). 20228203 10.1128/EC.00336-09 PMC2863949

[30] Vera, I. M., Beatty, W. L., Sinnis, P. & Kim, K. Plasmodium protease ROM1 is important for proper formation of the parasitophorous vacuole. *PLoS Pathog.* **7**, e1002197 (2011). 21909259 10.1371/journal.ppat.1002197 PMC3164628

[31] Tarun, A. S. et al. A combined transcriptome and proteome survey of malaria parasite liver stages. *Proc. Natl. Acad. Sci. USA.* **105**, 305–310 (2008). 18172196 10.1073/pnas.0710780104 PMC2224207

[32] Lindner, S. E. et al. Total and putative surface proteomics of malaria parasite salivary gland sporozoites. *Mol. Cell Proteom.* **12**, 1127–1143 (2013). 10.1074/mcp.M112.024505 PMC3650326 23325771

[33] Silvie, O., Briquet, S., Muller, K., Manzoni, G. & Matuschewski, K. Post-transcriptional silencing of UIS4 in Plasmodium berghei sporozoites is important for host switch. *Mol. Microbiol.* **91**, 1200–1213 (2014). 24446886 10.1111/mmi.12528

[34] Bennink, S. et al. A seven-helix protein constitutes stress granules crucial for regulating translation during human-to-mosquito transmission of Plasmodium falciparum. *PLoS Pathog.* **14**, e1007249 (2018). 30133543 10.1371/journal.ppat.1007249 PMC6122839

[35] Lindner, S. E. et al. Transcriptomics and proteomics reveal two waves of translational repression during the maturation of malaria parasite sporozoites. *Nat. Commun.* **10**, 4964 (2019). 31673027 10.1038/s41467-019-12936-6 PMC6823429

[36] Mair, G. R. et al. Regulation of sexual development of Plasmodium by translational repression. *Science* **313**, 667–669 (2006). 16888139 10.1126/science.1125129 PMC1609190

[37] Zhang, M., Joyce, B. R., Sullivan, W. J. Jr. & Nussenzweig, V. Translational control in Plasmodium and toxoplasma parasites. *Eukaryot. Cell* **12**, 161–167 (2013). 23243065 10.1128/EC.00296-12 PMC3571306

[38] Minns, A. M., Hart, K. J. Subramanian, S. Hafenstein, S. & Lindner, S. E. Nuclear, cytosolic, and surface-localized poly(A)-binding proteins of *Plasmodium yoelii*. *mSphere* **3**, e00435-17 10.1128/mSphere.00435-17 (2018). 10.1128/mSphere.00435-17 PMC5760745 29359180

[39] Munoz, E. E. et al. ALBA4 modulates its stage-specific interactions and specific mRNA fates during *Plasmodium yoelii* growth and transmission. *Mol. Microbiol.* **106**, 266–284 (2017). 28787542 10.1111/mmi.13762 PMC5688949

[40] Mangus, D. A., Evans, M. C. & Jacobson, A. Poly(A)-binding proteins: multifunctional scaffolds for the post-transcriptional control of gene expression. *Genome Biol.* **4**, 223 (2003). 12844354 10.1186/gb-2003-4-7-223 PMC193625

[41] Brook, M. et al. The multifunctional poly(A)-binding protein (PABP) 1 is subject to extensive dynamic post-translational modification, which molecular modelling suggests plays an important role in co-ordinating its activities. *Biochem. J.* **441**, 803–812 (2012). 22004688 10.1042/BJ20111474 PMC3298439

[42] Miao, J. et al. Puf mediates translation repression of transmission-blocking vaccine candidates in malaria parasites. *PLoS Pathog.* **9**, e1003268 (2013). 23637595 10.1371/journal.ppat.1003268 PMC3630172

[43] Lindner, S. E. et al. Perturbations of Plasmodium Puf2 expression and RNA-seq of Puf2-deficient sporozoites reveal a critical role in maintaining RNA homeostasis and parasite transmissibility. *Cell Microbiol.* **15**, 1266–1283 (2013). 23356439 10.1111/cmi.12116 PMC3815636

[44] Muller, K., Matuschewski, K. & Silvie, O. The Puf-family RNA-binding protein Puf2 controls sporozoite conversion to liver stages in the malaria parasite. *PLoS One* **6**, e19860 (2011). 21673790 10.1371/journal.pone.0019860 PMC3097211

[45] Lambert, N. et al. RNA Bind-n-Seq: quantitative assessment of the sequence and structural binding specificity of RNA binding proteins. *Mol. Cell* **54**, 887–900 (2014). 24837674 10.1016/j.molcel.2014.04.016 PMC4142047

[46] Ertekin, A., Morgan, B. R., Ryder, S. P. & Massi, F. Structure and dynamics of the CCCH-Type tandem zinc finger domain of POS-1 and implications for RNA binding specificity. *Biochem.-US/***63**, 2632–2647 (2024). 10.1021/acs.biochem.4c00259 PMC12288836 39321355

[47] Dutta, S., Madan, S. & Sundar, D. Exploiting the recognition code for elucidating the mechanism of zinc finger protein-DNA interactions. *BMC Genomics* **17**, 1037 10.1186/s12864-016-3324-8 (2016). 10.1186/s12864-016-3324-8 PMC5260074 28155654

[48] Bermudez, M., Moreno-Perez, D. A., Arevalo-Pinzon, G., Curtidor, H. & Patarroyo, M. A. *Plasmodium vivax* in vitro continuous culture: the spoke in the wheel. *Malar. J.* **17**, 301 (2018). 30126427 10.1186/s12936-018-2456-5 PMC6102941

[49] Voorberg-van der Wel, A. M. et al. A dual fluorescent *Plasmodium cynomolgi* reporter line reveals in vitro malaria hypnozoite reactivation. *Commun. Biol.* **3**, 7 (2020). 31909199 10.1038/s42003-019-0737-3 PMC6941962

[50] Guerreiro, A. et al. Genome-wide RIP-Chip analysis of translational repressor-bound mRNAs in the Plasmodium gametocyte. *Genome Biol.* **15**, 493 (2014). 25418785 10.1186/s13059-014-0493-0 PMC4234863

[51] Min, H. et al. The DEAD-box RNA helicase PfDOZI imposes opposing actions on RNA metabolism in Plasmodium falciparum. *Nat. Commun.* **15**, 3747 (2024). 38702310 10.1038/s41467-024-48140-4 PMC11068891

[52] Han, J. & Cooper, T. A. Identification of CELF splicing activation and repression domains. *Nucleic Acids Res.* **33**, 2769–2780 (2005). 15894795 10.1093/nar/gki561 PMC1126903

[53] Russell, A. J. C. et al. Regulators of male and female sexual development are critical for the transmission of a malaria parasite. *Cell Host Microbe*. **31**, 305–319 e310 (2023). 36634679 10.1016/j.chom.2022.12.011 PMC7616090

[54] Richter, J., Franken, G., Mehlhorn, H., Labisch, A. & Haussinger, D. What is the evidence for the existence of Plasmodium ovale hypnozoites? *Parasitol. Res*. **107**, 1285–1290 (2010). 20922429 10.1007/s00436-010-2071-z

[55] Veletzky, L. et al. Molecular evidence for relapse of an imported Plasmodium ovale wallikeri infection. *Malar. J.* **17**, 78 (2018). 29426330 10.1186/s12936-018-2226-4 PMC5807828

[56] De Meulenaere, K. et al. Band 3-mediated *Plasmodium vivax* invasion is associated with transcriptional variation in PvTRAg genes. *Front. Cell Infect. Microbiol.* **12**, 1011692 (2022). 36250048 10.3389/fcimb.2022.1011692 PMC9563252

[57] Wichers-Misterek, J. S. et al. The exception that proves the rule: virulence gene expression at the onset of Plasmodium falciparum blood stage infections. *PLoS Pathog.* **19**, e1011468 (2023). 37384799 10.1371/journal.ppat.1011468 PMC10337978

[58] Zanghi, G. et al. A Specific PfEMP1 Is Expressed in P. falciparum sporozoites and plays a role in hepatocyte infection. *Cell Rep.* **22**, 2951–2963 (2018). 29539423 10.1016/j.celrep.2018.02.075 PMC5863040

[59] Lopez-Barragan, M. J. et al. Directional gene expression and antisense transcripts in sexual and asexual stages of Plasmodium falciparum. *BMC Genomics* **12**, 587 (2011). 22129310 10.1186/1471-2164-12-587 PMC3266614

[60] Zhang, C. et al. Efficient editing of malaria parasite genome using the CRISPR/Cas9 system. *mBio*. **5**, e01414–e01414 (2014). 24987097 10.1128/mBio.01414-14 PMC4161241

[61] Vaughan, A. M. et al. A Plasmodium parasite with complete late liver stage arrest protects against preerythrocytic and erythrocytic stage infection in mice. *Infect. Immun.* **86**, e00088-18 10.1128/IAI.00088-18 (2018). 10.1128/IAI.00088-18 PMC5913857 29440367

[62] Janse, C. J., Ramesar, J. & Waters, A. P. High-efficiency transfection and drug selection of genetically transformed blood stages of the rodent malaria parasite Plasmodium berghei. *Nat. Protoc.* **1**, 346–356 (2006). 17406255 10.1038/nprot.2006.53

[63] Schafer, C. et al. A recombinant antibody against Plasmodium vivax UIS4 for distinguishing replicating from dormant liver stages. *Malar. J.* **17**, 370 (2018). 30333026 10.1186/s12936-018-2519-7 PMC6192329

[64] Miller, J. L., Harupa, A., Kappe, S. H. & Mikolajczak, S. A. Plasmodium yoelii macrophage migration inhibitory factor is necessary for efficient liver-stage development. *Infect. Immun.* **80**, 1399–1407 (2012). 22252874 10.1128/IAI.05861-11 PMC3318411

[65] Bergman, L. W. et al. Myosin A tail domain interacting protein (MTIP) localizes to the inner membrane complex of Plasmodium sporozoites. *J. Cell Sci.* **116**, 39–49 (2003). 12456714 10.1242/jcs.00194

[66] Kim, Y. J. et al. Purifying properly folded cysteine-rich, zinc finger containing recombinant proteins for structural drug targeting studies: the CH1 domain of p300 as a case example. *Bio Protoc.* **7**, e2537 10.21769/BioProtoc.2537 (2017). 10.21769/BioProtoc.2537 PMC5621770 28966947

[67] Lambert, N. J., Robertson, A. D. & Burge, C. B. RNA bind-n-seq: measuring binding affinity landscape of RNA-binding proteins. *Methods Enzymol.* **558**, 465–493 (2015). 26068750 10.1016/bs.mie.2015.02.007 PMC5576890

[68] Shen, W., Sipos, B. & Zhao, L. Y. SeqKit2: A Swiss army knife for sequence and alignment processing. *Imeta* **3**, e191 10.1002/imt2.191 (2024). 10.1002/imt2.191 PMC11183193 38898985

[69] Mirdita, M. et al. ColabFold: making protein folding accessible to all. *Nat. Methods* **19**, 679–682 (2022). 35637307 10.1038/s41592-022-01488-1 PMC9184281

[70] Pettersen, E. F. et al. UCSF ChimeraX: structure visualization for researchers, educators, and developers. *Protein Sci.* **30**, 70–82 (2021). 32881101 10.1002/pro.3943 PMC7737788

[71] Katoh, K. & Standley, D. M. MAFFT multiple sequence alignment software version 7: improvements in performance and usability. *Mol. Biol. Evol.* **30**, 772–780 (2013). 23329690 10.1093/molbev/mst010 PMC3603318

[72] Steenwyk, J. L., Loucks, J. T. & Buida, T. J. ClipKIT in the browser: fast online trimming of multiple sequence alignments for phylogenetics. *Nucleic Acids Res.* **53**, W169–W171 (2025). 40260744 10.1093/nar/gkaf325 PMC12230650

[73] Minh, B. Q. et al. IQ-TREE 2: new models and efficient methods for phylogenetic inference in the genomic era. *Mol. Biol. Evol.* **37**, 1530–1534 (2020). 32011700 10.1093/molbev/msaa015 PMC7182206

[74] Minh, B. Q. et al. Corrigendum to: IQ-TREE 2: new models and efficient methods for phylogenetic inference in the genomic era. *Mol. Biol. Evol.* **37**, 2461 (2020). 32556291 10.1093/molbev/msaa131 PMC7403609

[75] Letunic, I. & Bork, P. Interactive tree of life (iTOL) v6: recent updates to the phylogenetic tree display and annotation tool. *Nucleic Acids Res.* **52**, W78–W82 (2024). 38613393 10.1093/nar/gkae268 PMC11223838

