# Supplementary material for: Two Plasmodium vivax hypnozoite-expressed RNA-binding proteins inhibit liver stage replication
Source: Nat Commun. 2026 May 30;17:7048. doi: 10.1038/s41467-026-73666-0 (PMC13392028; doi:10.1038/s41467-026-73666-0)
Supplement: Supplementary file 1 — Supplementary Information [file 41467_2026_73666_MOESM1_ESM.pdf]

Figure S1

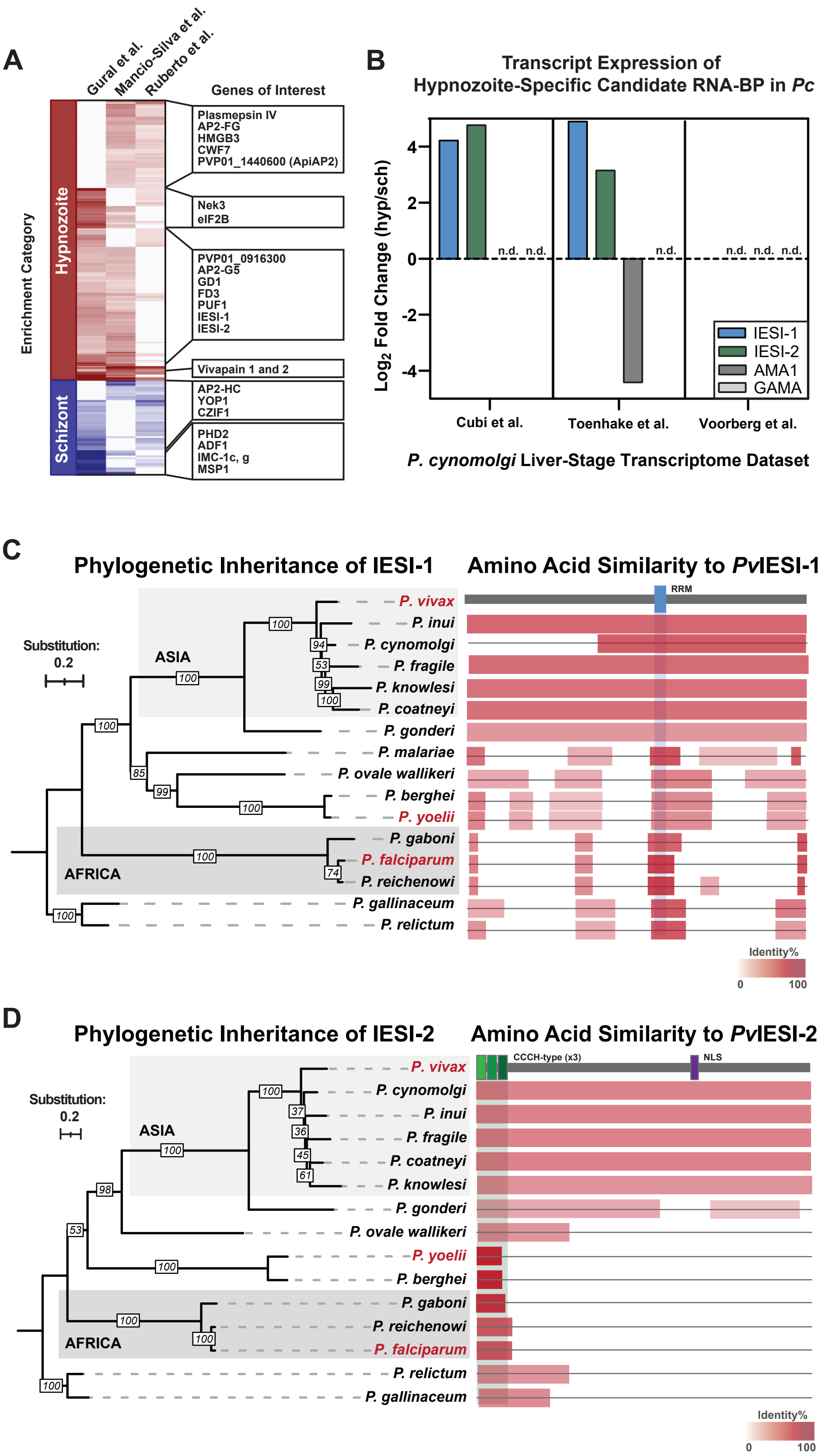
A
Gural et al.
Mancio-Silva et al.
Ruberto et al.
Genes of Interest
Enrichment Category
Hypnozoite
Schizont
Plasmepsin IV
AP2-FG
HMGB3
CWF7
PVP01_1440600 (ApiAP2)
Nek3
eIF2B
PVP01_0916300
AP2-G5
GD1
FD3
PUF1
IESI-1
IESI-2
Vivapain 1 and 2
AP2-HC
YOP1
CZIF1
PHD2
ADF1
IMC-1c, g
MSP1
B
Transcript Expression of Hypnozoite-Specific Candidate RNA-BP in Pc
Log2 Fold Change (hyp/sch)
n.d.
IESI-1
IESI-2
AMA1
GAMA
Cubi et al.
Toenhake et al.
Voorberg et al.
P. cynomolgi Liver-Stage Transcriptome Dataset
C
Phylogenetic Inheritance of IESI-1
Amino Acid Similarity to PvIESI-1
Substitution: 0.2
ASIA
AFRICA
RRM
P. vivax
P. inui
P. cynomolgi
P. fragile
P. knowlesi
P. coatneyi
P. gonderi
P. malariae
P. ovale wallikeri
P. berghei
P. yoelii
P. gaboni
P. falciparum
P. reichenowi
P. gallinaceum
P. relictum
Identity%
D
Phylogenetic Inheritance of IESI-2
Amino Acid Similarity to PvIESI-2
CCCH-type (x3)
NLS
Substitution: 0.2
ASIA
AFRICA
P. vivax
P. cynomolgi
P. inui
P. fragile
P. coatneyi
P. knowlesi
P. gonderi
P. ovale wallikeri
P. yoelii
P. berghei
P. gaboni
P. reichenowi
P. falciparum
P. relictum
P. gallinaceum
Identity%

Figure S1. Structure and conservation of IESI 1 and 2 proteins across diverse parasite species and in hypnozoite transcriptomes. (A) Comparison of published *P. vivax* hypnozoite transcriptomes. Differentially expressed transcripts from bulk RNA sequencing (Gural et al.[17], Mancio-silva et al.[18]) and scRNA-seq (Ruberto et al.)[19] datasets are shown in heatmap with specific examples highlighted. **(B)** Bargraph shows expression of markers of liver stage schizogony (AMA1, GAMA) and IESI-1 and IESI-2 with their previously reported relative expression in hypnozoites compared to liver schizonts in *P.cynomolgi* liver-stage transcriptome datasets[20-22]**.** For each IESI protein, a schematic of the *P. vivax* protein **(C, D)** indicates the relative position of the RNA binding domains and any other identifiable domains in the protein (NLS = Nuclear Localization Signal). Red bars indicate regions of homology where color intensity indicates relative sequence identity to the corresponding *P. vivax* protein. Phylogenetic trees show bootstrap values and length of branches indicate relative distance between species. Source data for this figure are provided in the Source Data file 3.

Figure S2

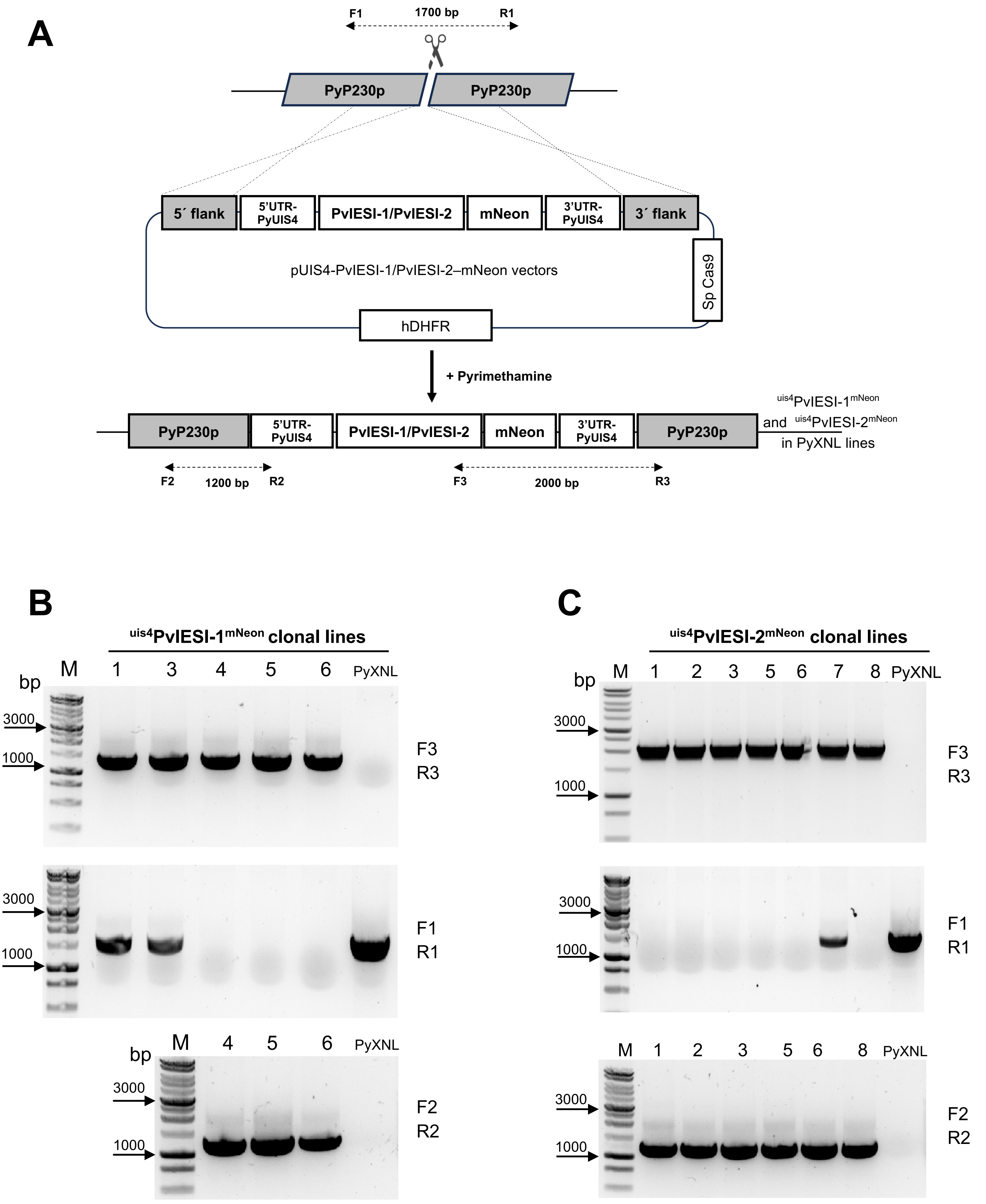

A
F1
1700 bp
R1
PyP230p
PyP230p
5´ flank
5'UTR-PyUIS4
PvIESI-1/PvIESI-2
mNeon
3'UTR-PyUIS4
3´ flank
pUIS4-PvIESI-1/PvIESI-2–mNeon vectors
Sp Cas9
hDHFR
+ Pyrimethamine
PyP230p
5'UTR-PyUIS4
PvIESI-1/PvIESI-2
mNeon
3'UTR-PyUIS4
PyP230p
uis4PvIESI-1mNeon and uis4PvIESI-2mNeon in PyXNL lines
F2
1200 bp
R2
F3
2000 bp
R3
B
uis4PvIESI-1mNeon clonal lines
M 1 3 4 5 6 PyXNL
bp
3000
1000
F3 R3
3000
1000
F1 R1
bp
M 4 5 6 PyXNL
3000
1000
F2 R2
C
uis4PvIESI-2mNeon clonal lines
M 1 2 3 5 6 7 8 PyXNL
bp
3000
1000
F3 R3
3000
1000
F1 R1
M 1 2 3 5 6 8 PyXNL
3000
1000
F2 R2

Figure S2. Generation and validation of ^{uis4}PvIESI-1^{mNeon} and ^{uis4}PvIESI-2^{mNeon} transgenic parasite lines.

(A) The scheme outlines the creation of ^{uis4}PvIESI-1^{mNeon} and ^{uis4}PvIESI-2^{mNeon} lines. For each construct, a plasmid containing Cas9, PyP230p-specific guide RNA, 5′/3′ PyP230p homology regions, 5′/3′ PyUIS4 UTRs, the codon optimized PvIESI-1 or PvIESI-2 ORFs, and mNeon was transfected into the PyXNL strain. Transgenic parasites were selected based on DHFR expression. The schematic diagram was generated using Microsoft PowerPoint, and the editable source file is provided within this published article.

(B–C) Agarose gel electrophoresis confirming successful integration and overexpression of ^{uis4}PvIESI-1^{mNeon} **(B)** and ^{uis4}PvIESI-2^{mNeon} **(C)** using recombinant-specific primers (labelled as F and R) as described in Table S2. Expected amplicon sizes for WT PyXNL, ^{uis4}PvIESI-1^{mNeon} and ^{uis4}PvIESI-2^{mNeon} are indicated in the schematic. M, Molecular weight marker; bp, base pairs. The uncropped gel images are provided in the Source Data file 4.

Figure S3

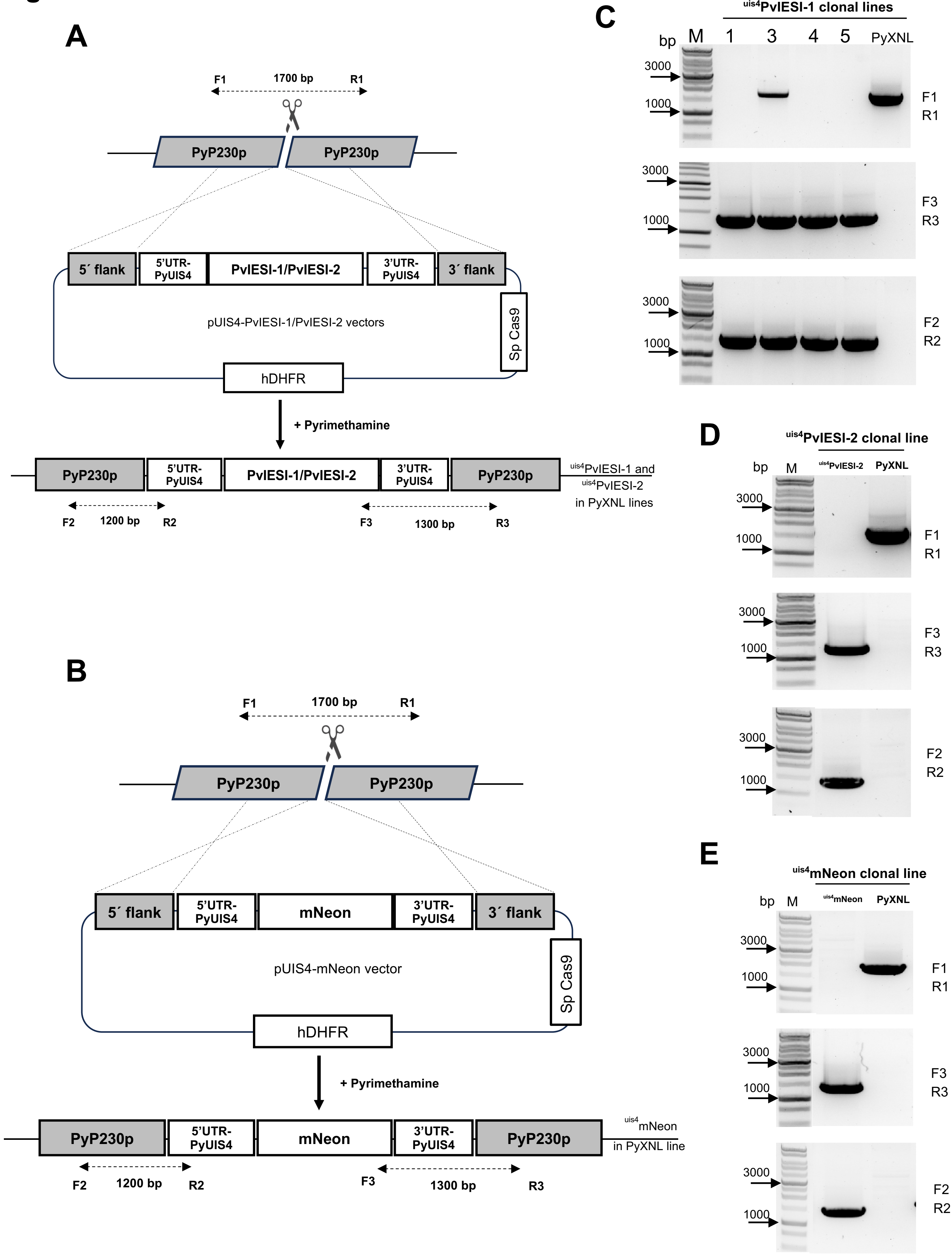

A
F1
1700 bp
R1
PyP230p
PyP230p
5´ flank
5'UTR-PyUIS4
PvIESI-1/PvIESI-2
3'UTR-PyUIS4
3´ flank
pUIS4-PvIESI-1/PvIESI-2 vectors
Sp Cas9
hDHFR
+ Pyrimethamine
PyP230p
5'UTR-PyUIS4
PvIESI-1/PvIESI-2
3'UTR-PyUIS4
PyP230p
uis4PvIESI-1 and uis4PvIESI-2 in PyXNL lines
F2
1200 bp
R2
F3
1300 bp
R3
B
F1
1700 bp
R1
PyP230p
PyP230p
5´ flank
5'UTR-PyUIS4
mNeon
3'UTR-PyUIS4
3´ flank
pUIS4-mNeon vector
Sp Cas9
hDHFR
+ Pyrimethamine
PyP230p
5'UTR-PyUIS4
mNeon
3'UTR-PyUIS4
PyP230p
uis4mNeon in PyXNL line
F2
1200 bp
R2
F3
1300 bp
R3
C
uis4PvIESI-1 clonal lines
bp
M
1
3
4
5
PyXNL
3000
1000
F1
R1
F3
R3
F2
R2
D
uis4PvIESI-2 clonal line
bp
M
uis4PvIESI-2
PyXNL
3000
1000
F1
R1
F3
R3
F2
R2
E
uis4mNeon clonal line
bp
M
uis4mNeon
PyXNL
3000
1000
F1
R1
F3
R3
F2
R2

Figure S3. Generation and validation of ^{uis4}PvIESI-1, ^{uis4}PvIESI-2, and ^{uis4}mNeon transgenic parasite lines.

(A–B) Schematic representation of the constructs used to generate ^{uis4}PvIESI-1, ^{uis4}PvIESI-2 parasite lines (**A**) and ^{uis4}mNeon line (**B**). A Cas9-expressing plasmid containing the PyP230p guide RNA, 5′/3′ PyP230p homology regions, 5′/3′ PyUIS4 UTRs, and either the condon optimized PvIESI-1 or PvIESI-2 ORF (without mNeon) (**A**) or only the mNeon ORF (**B**) was transfected into PyXNL parasites. Transgenic parasites were selected based on DHFR expression. The schematic diagrams were generated using Microsoft PowerPoint, and the editable source files is provided within this published article.

(C–E) PCR verification of transgenic clonal lines using recombinant-specific primers F and R (Table S2), confirming successful integration and overexpression of ^{uis4}PvIESI-1 (**C**), ^{uis4}PvIESI-2 (**D**), and ^{uis4}mNeon (**E**). Expected amplicon sizes for WT PyXNL, ^{uis4}PvIESI-1, ^{uis4}PvIESI-2, and ^{uis4}mNeon lines are indicated in the schematic. M, Molecular weight marker; bp, base pairs. The uncropped gel images are provided in the Source Data file 4.

Figure S4

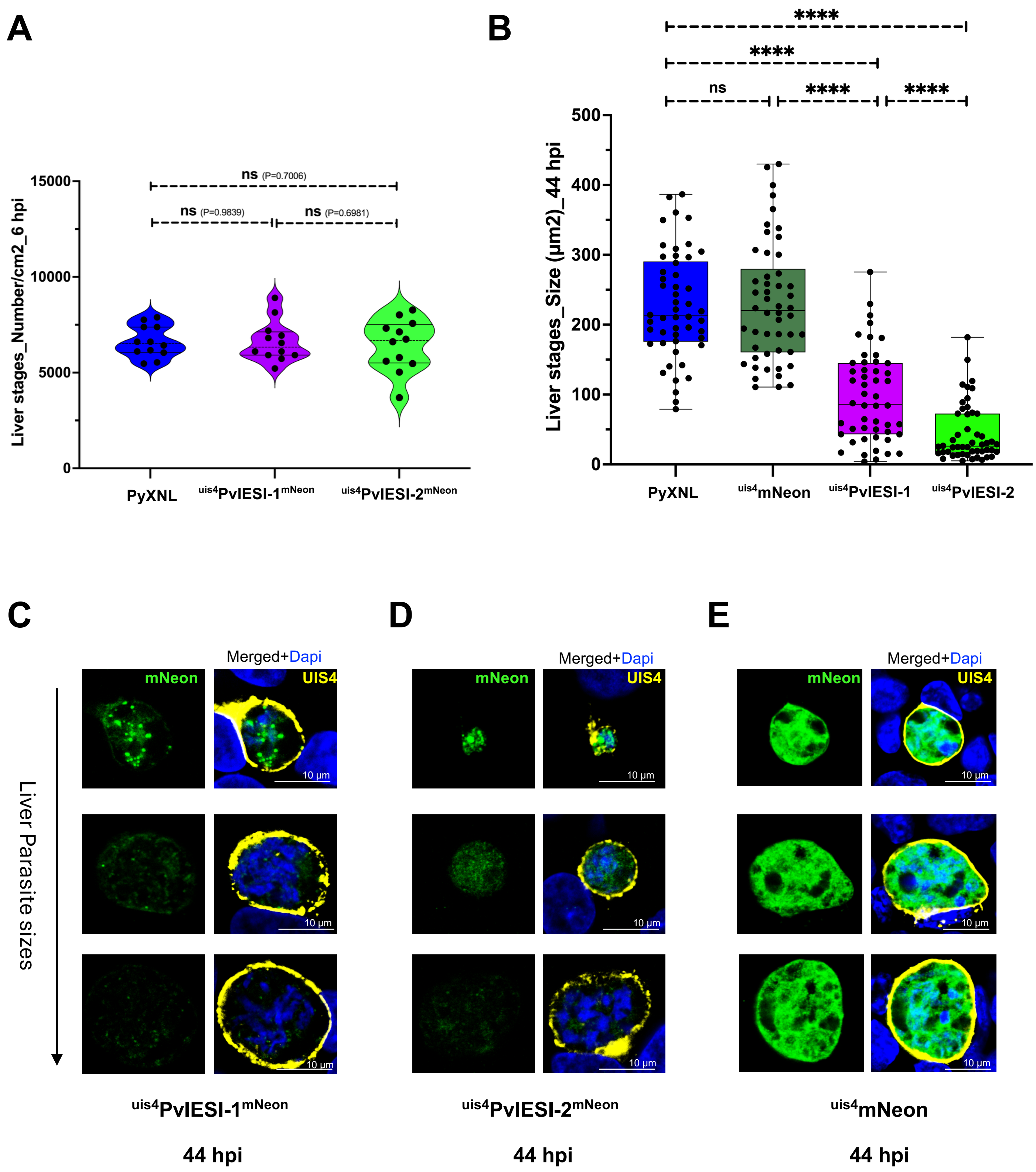
A
Liver stages_Number/cm2_6 hpi
15000
10000
5000
0
ns (P=0.7006)
ns (P=0.9839)
ns (P=0.6981)
PyXNL
uis4PvIESI-1mNeon
uis4PvIESI-2mNeon
B
Liver stages_Size (µm2)_44 hpi
500
400
300
200
100
0
****
****
ns
****
****
PyXNL
uis4mNeon
uis4PvIESI-1
uis4PvIESI-2
C
D
E
mNeon
Merged+Dapi
UIS4
10 µm
Liver Parasite sizes
uis4PvIESI-1mNeon
44 hpi
uis4PvIESI-2mNeon
44 hpi
uis4mNeon
44 hpi

Figure S4. Stable liver stage growth with differential mNeon signal dynamics in PvIESI-1 and PvIESI-2 overexpressing lines. (A) No significant differences in liver-stage parasite numbers were observed at 6 hpi between the PyXNL control and the ^{uis4}PvIESI-1, ^{uis4}PvIESI-2 lines. Data are shown as mean ± SD from four independent experiments (n=4). Statistical significance was assessed using two-way ANOVA followed by Tukey's multiple comparisons test, with $P > 0.05$ considered not significant. For each group, 12 randomly selected fields per 8-well plate were imaged at 20× magnification using a Keyence microscope and used to quantify parasite numbers. The violin plots illustrate the full distribution of the data, with the central line representing the median. All individual data points are shown, and each point corresponds to the mean parasite number per region per cm^2 (12 regions per cm^2 in total) across four independent experiments.

(**B**) The graph shows liver-stage parasite sizes across four parasite strains (PyXNL, ^{uis4}mNeon, ^{uis4}PvIESI-1, ^{uis4}PvIESI-2). Data are presented as mean ± SD from three independent experiments (n = 3). For each group, the sizes of 150 *P. yoelii* liver-stage schizonts were measured, with 50 parasites analyzed per independent experiment. Statistical significance was evaluated using two-way ANOVA followed by Tukey's multiple comparisons test. $P > 0.05$ was considered not significant (ns), whereas $P < 0.05$ was considered significant, ****$P < 0.0001$. Box plots display the median (central line), with whiskers indicating the minimum and maximum values; each data point represents the mean size of three parasites per independent experiment across three independent experiments (50 data points in total). Source data for this figure are provided in the Source Data file 4.

(C–E) IFA of ^{uis4}PvIESI-1, ^{uis4}PvIESI-2 and ^{uis4}mNeon lines infected HepG2-CD81 cells at 44 hpi. Parasites were analyzed using UIS4 (yellow) to visualize PVM development, and mNeon (green) to detect the PvIESI-1 or PvIESI-2 expressing mNeon signal. The mNeon signal gradually decreased as parasite size increased in ^{uis4}PvIESI-1^{mNeon} line **(C)** and ^{uis4}PvIESI-2^{mNeon} line **(D).** Conversely, the mNeon signal remained stable in ^{uis4}mNeon parasites **(E).** The staining patterns and mNeon signal dynamics were consistently observed across three independent experiments.

Figure S5

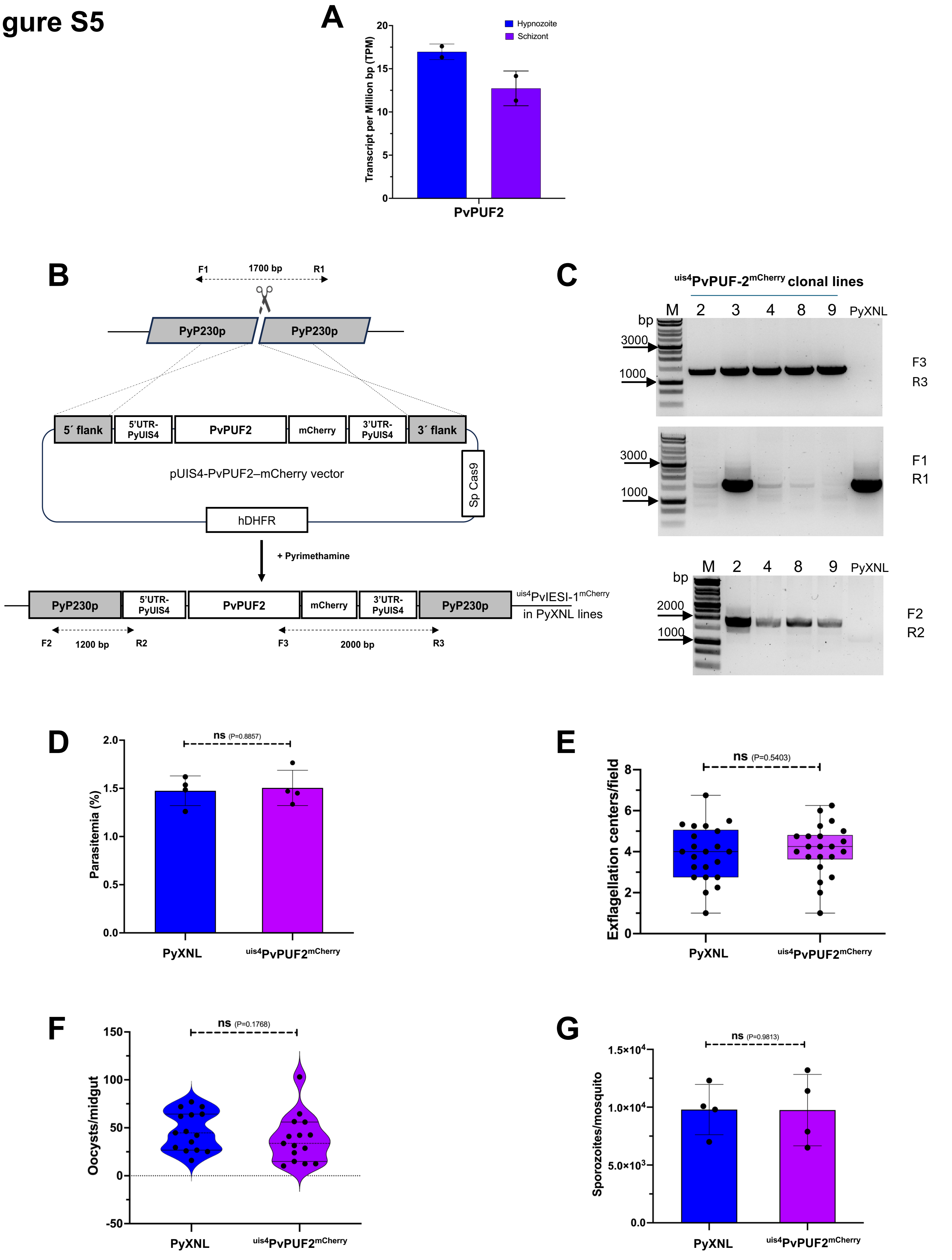

A
Hypnozoite
Schizont
Transcript per Million bp (TPM)
PvPUF2
B
F1
1700 bp
R1
PyP230p
PyP230p
5´ flank
5'UTR-PyUIS4
PvPUF2
mCherry
3'UTR-PyUIS4
3´ flank
pUIS4-PvPUF2–mCherry vector
Sp Cas9
hDHFR
+ Pyrimethamine
uis4PvIESI-1mCherry in PyXNL lines
F2
1200 bp
R2
F3
2000 bp
R3
C
uis4PvPUF-2mCherry clonal lines
M
PyXNL
bp
3000
1000
2000
F3
R3
F1
R1
F2
R2
D
ns (P=0.8857)
Parasitemia (%)
PyXNL
uis4PvPUF2mCherry
E
ns (P=0.5403)
Exflagellation centers/field
F
ns (P=0.1768)
Oocysts/midgut
G
ns (P=0.9813)
Sporozoites/mosquito

Figure S5. Generation and validation of the ^{uis4}PvPUF2^{mCherry} transgenic line.

(A) Reported transcriptomic data showing the upregulated PvPUF2 transcript levels in hypnozoites and schizonts, based on bulk RNA-seq data from Gural et al.[17]. data are presented as mean ± SD (n=2 biological replicates, represented by two data points). **(B)** Schematic diagram illustrating the generation of the ^{uis4}PvPUF2^{mCherry} parasite line. A plasmid containing Cas9, a PyP230p-specific guide RNA, 5′ and 3′ PyP230p homology arms, 5′/3′ PyUIS4 UTRs, the condon optimized PvPUF2 ORF, and mCherry was transfected into the PyXNL strain. Transgenic parasites were selected via DHFR expression. The schematic diagrams were generated using Microsoft PowerPoint, and the editable source files is provided within this published article.

(C) PCR verification of transgenic clonal lines using recombinant-specific primers F and R (Table S2), confirming successful integration and overexpression of ^{uis4}PvPUF2^{mCherry}. Expected amplicon sizes are indicated in the schematic. M, Molecular weight marker; bp, base pairs. The uncropped gel images are provided in the Source Data file 4.

(D–G) Asexual blood-stage parasitemia **(D)**, male gamete emergence (exflagellation centers counted across 22 random fields, 7–10 min post-blood smear) **(E)**, mosquito oocyst counts (day 8) (**F)**, and SG Spz counts (days 14–15) **(G)** showed no significant differences between PyXNL and ^{uis4}PvPUF2^{mCherry} lines. In (**D–G**), data are presented as mean ± SD from four independent experiments (n=4). Statistical analysis was performed using two-sided non-parametric Mann–Whitney U-test. A P value >0.05 was considered not significant (ns). In (**D**), each data point represents the mean parasitemia per experiment (n=4). In (**E**), box plot shows the median (central line), while whiskers indicate the minimum and maximum values; individual points represent the mean of four exflagellation centers across four independent experiments (22 total data points). In **(F)**, violin plots with central line indicates the median; individual points represent the mean number of oocysts per four mosquitoes across four independent experiments (15 total data points). In (**G**), each data point represents the mean number of sporozoites per mosquito from four independent experiments. Source data for this figure are provided in the Source Data file 4.

Figure S6

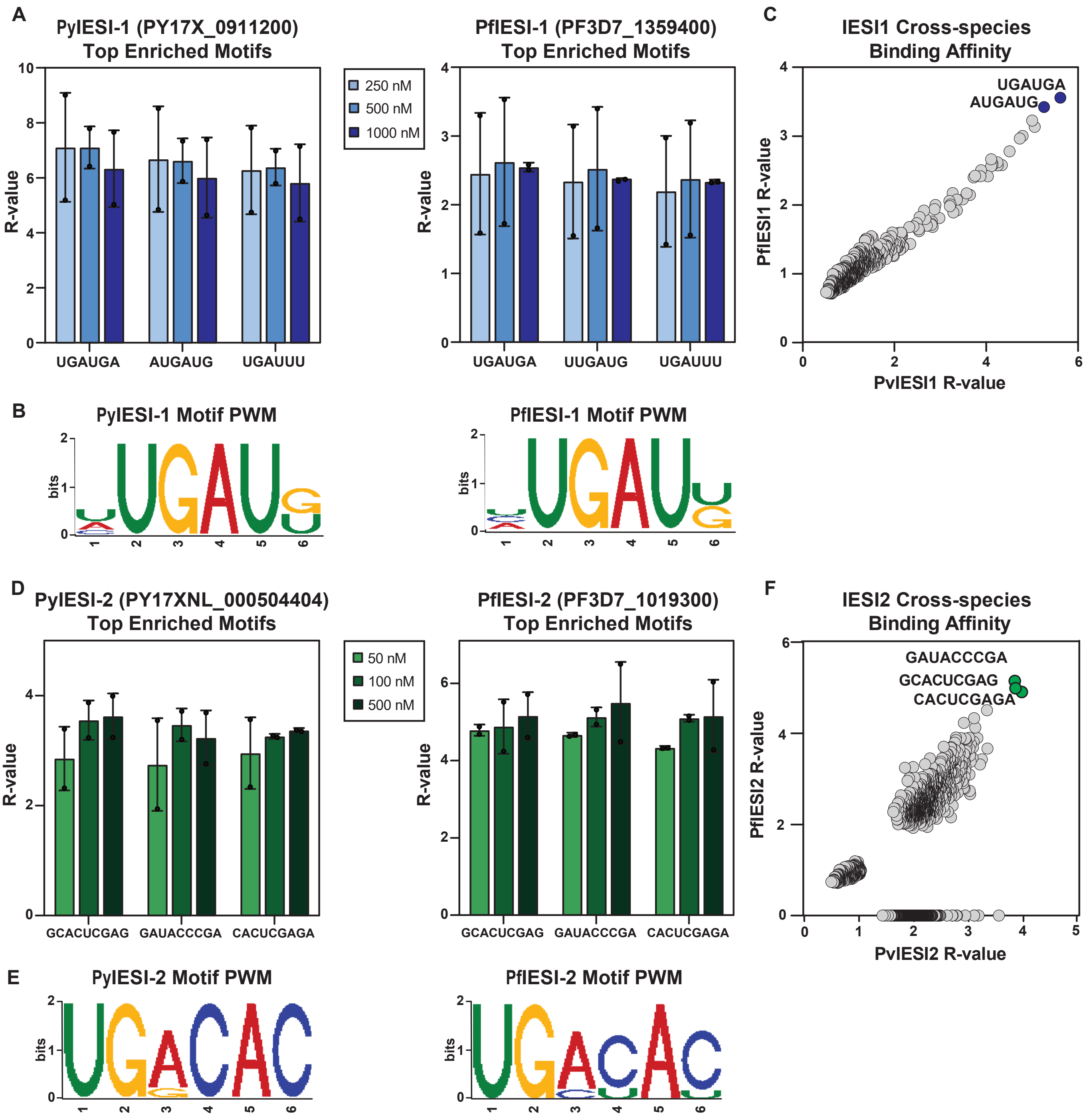

A
PyIESI-1 (PY17X_0911200)
Top Enriched Motifs
250 nM
500 nM
1000 nM
R-value
UGAUGA
AUGAUG
UGAUUU
PfIESI-1 (PF3D7_1359400)
Top Enriched Motifs
UUGAUG
C
IESI1 Cross-species
Binding Affinity
PfIESI1 R-value
PvIESI1 R-value
B
PyIESI-1 Motif PWM
PfIESI-1 Motif PWM
bits
D
PyIESI-2 (PY17XNL_000504404)
Top Enriched Motifs
50 nM
100 nM
500 nM
GCACUCGAG
GAUACCCGA
CACUCGAGA
PfIESI-2 (PF3D7_1019300)
Top Enriched Motifs
F
IESI2 Cross-species
Binding Affinity
PfIESI2 R-value
PvIESI2 R-value
E
PyIESI-2 Motif PWM
PfIESI-2 Motif PWM

Figure S6. Determining the *in vitro* RNA binding specificity of IESI-1 and IESI-2 across species. (A, D) Results from RNA-bind-n-seq show the top motifs enriched above occurrences of all possible 6-mers as R-values for *P. falciparum* and *P. yoelii* IESI-1 and IESI-2 respectively. The individual two technical replicates are indicated as closed circles. **(B, E)** Representative sequence logo of 10 top motifs enriched in RNA-bind-n-seq was generated in seqlogo in R for for *P. falciparum* and *P. yoelii* IESI-1 **(B)** and IESI-2 **(E)**. Correlation between the average enrichment (R-values) obtained for motifs in the *P. falciparum* and *P. vivax* IESI-1 **(C)** and IESI-2 **(F)** experiments with top motifs highlighted in color. Source data for this figure are provided in the Source Data file 1.

Figure S7

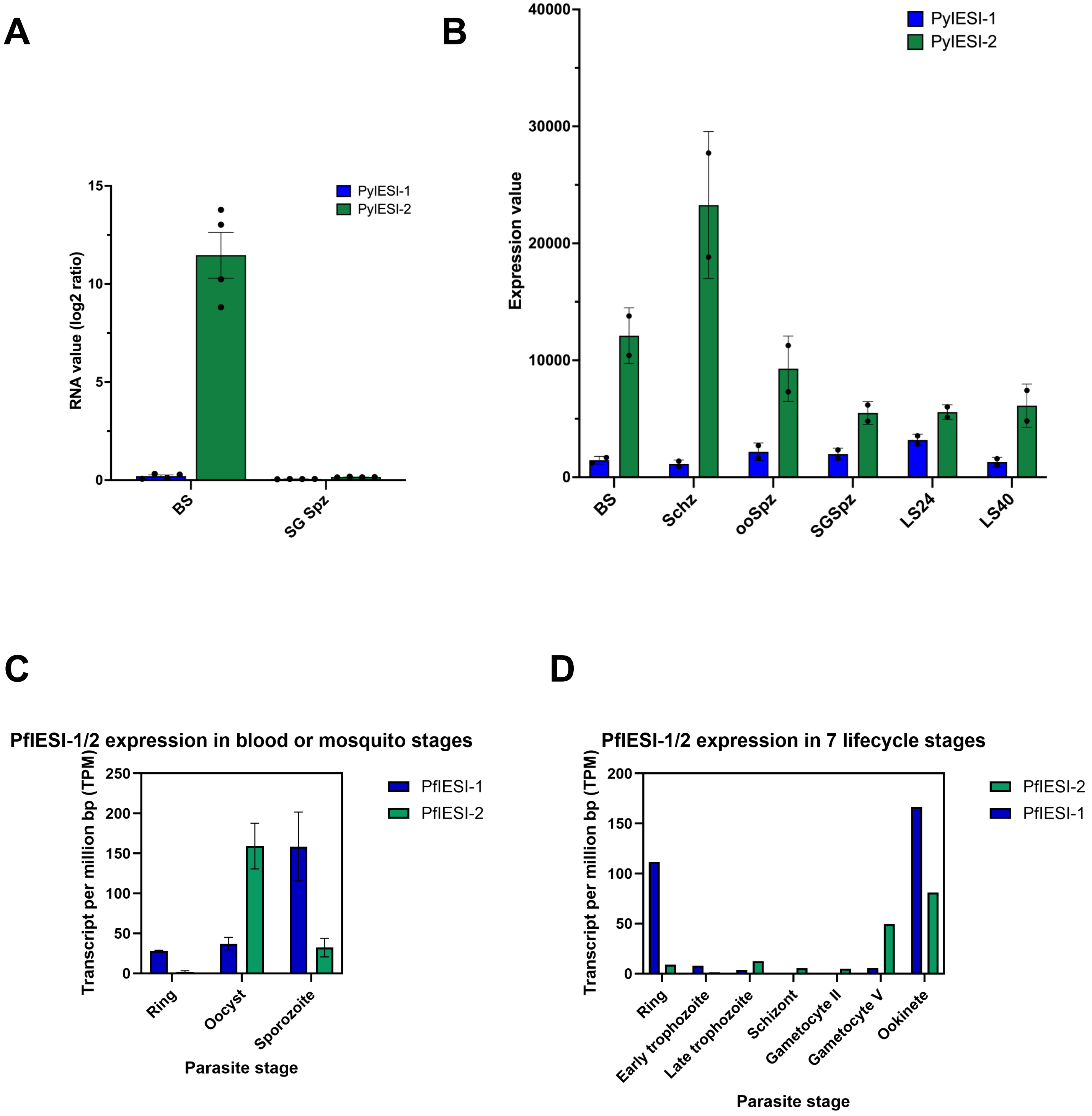
A
B
C
D
PyIESI-1
PyIESI-2
RNA value (log2 ratio)
BS
SG Spz
Expression value
Schz
ooSpz
SGSpz
LS24
LS40
PfIESI-1/2 expression in blood or mosquito stages
Transcript per million bp (TPM)
Ring
Oocyst
Sporozoite
Parasite stage
PfIESI-1
PfIESI-2
PfIESI-1/2 expression in 7 lifecycle stages
Early trophozoite
Late trophozoite
Schizont
Gametocyte II
Gametocyte V
Ookinete

Figure S7. Gene expression of PyIESI-1 and 2 and PfIESI-1 and 2 across the *Plasmodium* life cycle.

(A) RT–qPCR analysis of PyIESI-1 and PyIESI-2 transcript levels in blood stages and SG sporozoites. Relative gene expression was calculated using the 2^−ΔΔCt method, with normalization to the control Py18S RNA gene. Data are presented as mean ± SD from four independent experiments (n=4). Each data point represents the mean value of two technical replicates within each independent experiment. **(B)** Published transcriptomic profiles of PyIESI-1 and PyIESI-2 across the parasite life cycle, with highest expression of PyIESI-2 transcripts observed in blood stages, while PyIESI-1 transcripts consistently low expression across parasite life cycle. Data retrieved from Tarun et al[31] based on microarray analysis (Data are presented as mean ± SD, n=2 biological replicates, represented by two data points). BS, blood stages; Schz, schizonts; ooSpz, oocyst sporozoites; SGSpz, salivary gland sporozoites; LS24, liver-stage 24 hpi; LS40, liver-stage 40 hpi. Previously reported transcript levels of PfIESI-1/2 are shown for RNA-seq of **(C)** Ring, Oocyst and Sporozoite stages published in Zanghi et al.[58] (Data are presented as mean ± SD, n=2 biological replicates). or **(D)** seven life-cycle stages encompassing asexual and sexual blood-stage development and ookinetes published in in Lopez-Barragan et al.[59] (Data derived from one biological replicate). Source data and the reported data from published studies for this figure are included in the Source Data file 4.

Table S1. Blood stage patency analysis of the ^{uis4}PvPUF2^{mCherry} line compared with wildtype PyXNL.

Mouse strain	Parasite strain	^{a}Number of sporozoite	^{b}Number of mice patency (days to patency)*
SW	PyXNL	10.000	6/6 (3)
SW	^{uis4}PyPUF2^{mCherry}	10.000	6/6 (3)

Table S1. Blood stage patency analysis of the ^{uis4}PvPUF2^{mCherry} line compared with wildtype PyXNL. The table summarizes the time to blood stage patency following sporozoite infection, showing no observable delay in the liver-to-blood stage transition in the ^{uis4}PvPUF2^{mCherry} line when compared to PyXNL. The table summarizes patency results: ^{a}salivary gland sporozoites injected per mouse, and ^{b}patent mice per total infected, with *days to patency in parentheses.

Table S2. Primers used for vector construction and genomic screening of transgenic parasites.

Primer Name	Primer Sequence (5'-3')	Vector/Target
HindIII-PyP230p 5'flank-F1	CTCATCAAGCTTGAAGATTTTATCATTATTCGAG	Construction of the pUIS4 vector containing 5'/3' P230p flanking regions and 5'/3' PyUIS4 UTRs
AscI-PyP230p 5'flank-R1	CTCATCGGCGCGCCGGTTAGTGGATATGATCCAT	
Sa1I-PyP230 3'flank-F1	CTCATCGTCGACGACTGATTATAGAAGAGCAATTA	
NotI-PyP230 3'flank-R2	CTCATC GCGGCCGCGAAGTTTGATTCTTAATTCC	
NheI-PyUIS4 3'UTR-F1	TATATACGCGTTGCTAGCCATTATGAGGGTAATTCAGAAAGAGAAC	
Sa1I-PyUIS4 3'UTR-R1	ATA TAGCGG CCG CCA GGT TTG CAT ATA CGG CTG TTT TC	
PUIS4 5'UTR-F1	TATATGGCGCGCCTTTTTCTTTTAATGTATTAATTAGTGTAATAATTTTGTAAAAACTTTATTCATTTATTTATTTTCCGTACTACATTG	
PyUIS4 5'UTR-R1	ATA TAG TCG ACA GTT TAA ACT TTA TTC AGA TGT AAT TAT GTA CTA AAG GGT ATA TAT GTA GTC AAT ATC GAT C	
PyP230-mid ORF-Guide F	tattGAATGTGGTGTAACAAATGG	To insert sgRNA targeting the middle of the PyP230p ORF into pUIS4 vector.
PyP230-mid ORF-Guide R	aaacCCATTTGTTACACCACATTC	
KpnI-linker-mNeon-F1	CTCATCGGTACCGGGGGAGGTGGCTCCATGGTTAGTAAAGGAGAAGAA	Construction of the pUIS4-mNeon vector
NheI-mNeon-R1	CTCATCGCTAGCTTACATCATATCTGTAAATGCTTTTTGCCATTC	
KpnI-linker-mcherry-F1	ctcatcGGTACCGGGGGAGGTGGCGTTTCAAAAGGAGAAGAA	Construction of the pUIS4-mCherry vector
NheI-mcherry-R1	ctcatcGCTAGCTTATTTATATAATTCATCCATTCC	
PyP230p 5'flank-Scr-F1	CCTCCATATAGCAATAAAACAGGGGATC	For clonal line screening of the uis4-PvIESI-1-mNeon, uis4-PvIESI-2-mNeon, uis4-PvIESI-1, uis4-PvIESI-2, and uis4-mNeon, and uis4-PvPUF2-mCherry parasite lines.
PyP230p 3'flank-Scr-R1	GGTATAACATCTGTTATGCTTGATACACTTTGC	
PvIESI-1-ORF-Scr-F1	TGGAGATCTTCAACAAGTTC	
PvIESI-2- ORF-Scr-F1	GACGGAGCATTTTGTGTTGA	
PyUSI4 5'UTRmid-Scr-R1	GTCAATGGTAATATCCGCATGTATCC	
mNeon-Scr-F1	GAACCAACCAATGTATGTATTTCGCAAAAC	

PvPuf2-ORF-5end-Scr-R1	CTTGGTAATGATTCGTCTAAAAATGCACTTTC	
PvPuf2-ORF-3end-Scr-F1	GCGATTTGTAAAGATTGTTATGG	
mCherry-3end-Scr-F1	GAACAATATGAAAGAGCAGAAGGAAGACATAG	
PvIESI-1-qPCR-F1	GCTTATGAACGAGGACGATCAG	For RT-qPCR analysis to quantify transcript levels of PvIESI-1 and PvIESI-2, as well as PyIESI-1 and PyIESI-2.
PvIESI-1-qPCR-R1	CCTTACCTACTCCAGCCATTTC	
PvIESI-2-qPCR-F1	CAAGACGGCAAAGAGGACTATC	
PvIESI-2-qPCR-R1	CACACTCTCTATCGGTCCATTTC	
PyIESI-1-qPCR-F1	GAGAATGATGATCGTGCCAGTA	
PyIESI-1-qPCR-R1	GCAGATCTACCCATCTCGATATTC	
PyIESI-2-qPCR-F1	GTGGAAAGTGGTGCAGTAGAA	
PyIESI-2-qPCR-R1	GCCAACCTTTCCAACTTTGC	
Py18s rRNA-F1	GGAAGTTTAAGGCAACAACAGG	
Py18s rRNA-R1	CACCCACGCGCAGATATAA	
