# Supplementary material for: Two Plasmodium vivax hypnozoite-expressed RNA-binding proteins inhibit liver stage replication
Source: Nat Commun. 2026 May 30;17:7048. doi: 10.1038/s41467-026-73666-0 (PMC13392028; doi:10.1038/s41467-026-73666-0)
Supplement: Supplementary file 2 — Description Of Additional Supplementary File [file 41467_2026_73666_MOESM2_ESM.pdf]

Description of Additional supplementary files

Supplementary data 1: RBNS results for IESI proteins across species, including enriched 6-mers (IESI-1) and 9-mers (IESI-2), averaged per replicate.

Supplementary data 2: Genome-wide IESI-1/IESI-2 motif locations in P. vivax P01 transcriptomes, STREME motifs, motif occurrences in differentially expressed transcripts, and GO enrichment analyses for IESI-1 (liver schizonts) and IESI-2 (mixed populations).
