# Supplementary material for: Two Plasmodium vivax hypnozoite-expressed RNA-binding proteins inhibit liver stage replication
Source: Nat Commun. 2026 May 30;17:7048. doi: 10.1038/s41467-026-73666-0 (PMC13392028; doi:10.1038/s41467-026-73666-0)
Supplement: Supplementary file 5 — Reporting summary [file 41467_2026_73666_MOESM5_ESM.pdf]

nature portfolio

Corresponding author(s): Kim Chi Vo
Stefan H.I. Kappe

Last updated by author(s): 04 May 2026

Reporting Summary

Nature Portfolio wishes to improve the reproducibility of the work that we publish. This form provides structure for consistency and transparency in reporting. For further information on Nature Portfolio policies, see our Editorial Policies and the Editorial Policy Checklist.

Statistics

For all statistical analyses, confirm that the following items are present in the figure legend, table legend, main text, or Methods section.

n/a	Confirmed	
☐	☒	The exact sample size (*n*) for each experimental group/condition, given as a discrete number and unit of measurement
☒	☐	A statement on whether measurements were taken from distinct samples or whether the same sample was measured repeatedly
☐	☒	The statistical test(s) used AND whether they are one- or two-sided<br>*Only common tests should be described solely by name; describe more complex techniques in the Methods section.*
☒	☐	A description of all covariates tested
☒	☐	A description of any assumptions or corrections, such as tests of normality and adjustment for multiple comparisons
☒	☐	A full description of the statistical parameters including central tendency (e.g. means) or other basic estimates (e.g. regression coefficient) AND variation (e.g. standard deviation) or associated estimates of uncertainty (e.g. confidence intervals)
☒	☐	For null hypothesis testing, the test statistic (e.g. *F*, *t*, *r*) with confidence intervals, effect sizes, degrees of freedom and *P* value noted<br>*Give P values as exact values whenever suitable.*
☒	☐	For Bayesian analysis, information on the choice of priors and Markov chain Monte Carlo settings
☒	☐	For hierarchical and complex designs, identification of the appropriate level for tests and full reporting of outcomes
☒	☐	Estimates of effect sizes (e.g. Cohen's *d*, Pearson's *r*), indicating how they were calculated

Our web collection on statistics for biologists contains articles on many of the points above.

Software and code

Policy information about availability of computer code

Data collection

- Database used for accessing Plasmodium data: www.Plasmodb.org

- IFA images were acquired using Lightning Software and LAS X Life Science Microscope Software.

- RT-qPCR data were generated on the QuantStudio 5 Real-Time PCR system.

- All software packages used for data collection are publicly available and open source; software versions are listed below. Briefly:
• RNA bind-n-seq (RBNS): RBNS libraries were pooled and subjected to single-end sequencing on a MiSeq V3 platform.
• Motif occurrence analysis: To identify occurrences of RBNS-derived motifs in transcripts, a FASTA file containing all P. vivax transcript sequences was scanned with SeqKit v2.10.0 for the motifs 5′-TGATGA-3′ or 5′-TGACAC-3′. Identified motif locations were then mapped to annotated gene features (introns, exons, UTRs) using the corresponding P. vivax GTF file.
• Motif enrichment in hypnozoite-associated transcripts: Enriched sequence motifs were identified in hypnozoite-associated transcripts using data from Gural et al. (2018). Transcripts upregulated by >1 $\log_2$ fold change with P < 0.05 in hypnozoites or liver-stage parasites were analyzed for enriched motifs relative to all P. vivax transcripts using STREME (https://meme-suite.org/meme/doc/streme.html).

Data analysis

- IFA image processing was carried out with Lightning Software and LAS X Life Science Microscope Software.

-Statistical analyses and data visualization were performed using GraphPad Prism v10.3.0. Comparisons between two groups were conducted using a two-sided nonparametric Mann–Whitney U test, while comparisons among three groups were analyzed by two-way ANOVA followed by Tukey’s multiple comparisons test. A P value < 0.05 was considered statistically significant (*p < 0.05; **p < 0.01; ***p < 0.001; ****p < 0.0001).

All software used for data analysis is publicly available and open-source; specific versions are reported.
• RBNS data analysis: RBNS datasets were analyzed in Linux using scripts from the Burge Lab RBNS pipeline to identify enriched RNA-binding sequences (https://github.com/cburgelab/RBNS_pipeline).
• Motif visualization: Representative sequence logos of enriched motifs from RNA-bind-n-seq were generated using the seqLogo package (DOI: 10.18129/B9.bioc.seqLogo) in R v4.2.3.
• Protein structure prediction: Predicted structures of the RRM domain (aa 1,043–1,123) in PVP01_0939900 and the C3H1-type domain (aa 1–100) in PVP01_0604500 were generated with ColabFold v1.5.5 using default parameters. The top-ranked models were visualized in ChimeraX v1.9. Sequence conservation was evaluated by aligning the corresponding residues in P. yoelii (RRM domain in PY17X_0911200 and C3H1-type domain in Py17XNL_000504404).
• Phylogenetic analysis: Protein sequences were retrieved using BLASTP for PVP01_0939900 (IESI-1) and PVP01_0604500 (IESI-2). Sequences were aligned with MAFFT v7.525 (default parameters) and trimmed with ClipKIT v2.1.3 using the "smart-gap" mode. Trimmed alignments were used to infer maximum-likelihood trees with IQ-TREE v3.0.1, using 1,000 ultrafast bootstrap replicates and the best-fit substitution models (IESI-1: Q.BIRD+F+I+G4; IESI-2: Q.MAMMAL+F+I+G4). P. gallinaceum and P. relictum were used as outgroups to root the trees, which were visualized and exported using iTOL.

For manuscripts utilizing custom algorithms or software that are central to the research but not yet described in published literature, software must be made available to editors and reviewers. We strongly encourage code deposition in a community repository (e.g. GitHub). See the Nature Portfolio guidelines for submitting code & software for further information.

Data

Policy information about availability of data

All manuscripts must include a data availability statement. This statement should provide the following information, where applicable:

- Accession codes, unique identifiers, or web links for publicly available datasets
- A description of any restrictions on data availability
- For clinical datasets or third party data, please ensure that the statement adheres to our policy

For the RNA-bind-n-seq results, raw sequencing reads have been submitted to the SRA: PRJNA1372697 [https://www.ncbi.nlm.nih.gov/bioproject/?term=(PRJNA1372697)] with public access and the processed data are included in Supplementary File 1. The Figure and Supplementary Figure data generated in this study are provided in the Source Data files. Source Data files are provided with this paper.

Research involving human participants, their data, or biological material

Policy information about studies with human participants or human data. See also policy information about sex, gender (identity/presentation), and sexual orientation and race, ethnicity and racism.

Reporting on sex and gender	*Use the terms sex (biological attribute) and gender (shaped by social and cultural circumstances) carefully in order to avoid confusing both terms. Indicate if findings apply to only one sex or gender; describe whether sex and gender were considered in study design; whether sex and/or gender was determined based on self-reporting or assigned and methods used.*<br>*Provide in the source data disaggregated sex and gender data, where this information has been collected, and if consent has been obtained for sharing of individual-level data; provide overall numbers in this Reporting Summary. Please state if this information has not been collected.*<br>*Report sex- and gender-based analyses where performed, justify reasons for lack of sex- and gender-based analysis.*
Reporting on race, ethnicity, or other socially relevant groupings	*Please specify the socially constructed or socially relevant categorization variable(s) used in your manuscript and explain why they were used. Please note that such variables should not be used as proxies for other socially constructed/relevant variables (for example, race or ethnicity should not be used as a proxy for socioeconomic status).*<br>*Provide clear definitions of the relevant terms used, how they were provided (by the participants/respondents, the researchers, or third parties), and the method(s) used to classify people into the different categories (e.g. self-report, census or administrative data, social media data, etc.)*<br>*Please provide details about how you controlled for confounding variables in your analyses.*
Population characteristics	*Describe the covariate-relevant population characteristics of the human research participants (e.g. age, genotypic information, past and current diagnosis and treatment categories). If you filled out the behavioural & social sciences study design questions and have nothing to add here, write "See above."*
Recruitment	*Describe how participants were recruited. Outline any potential self-selection bias or other biases that may be present and how these are likely to impact results.*
Ethics oversight	*Identify the organization(s) that approved the study protocol.*

Note that full information on the approval of the study protocol must also be provided in the manuscript.

Field-specific reporting

Please select the one below that is the best fit for your research. If you are not sure, read the appropriate sections before making your selection.

☒ Life sciences ☐ Behavioural & social sciences ☐ Ecological, evolutionary & environmental sciences

For a reference copy of the document with all sections, see nature.com/documents/nr-reporting-summary-flat.pdf

Life sciences study design

All studies must disclose on these points even when the disclosure is negative.

Sample size	At least three biological replicates of P. yoelii salivary gland sporozoites from each of the seven parasite lines were used to infect HepG2-CD81 cells. Additionally, a minimum of three biological replicates per liver-stage time point (6, 12, 24, and 44 hpi) were included to enable robust quantification of liver-stage parasite size and number. All attempts at replication in this study were successful.
Data exclusions	N/A
Replication	Every experiment has been performed with a minimum of three biological replicates
Randomization	For all experiments, animals were randomly assigned to experimental groups. Mice of similar age and the same sex were randomly selected for each study. For experiments not involving animals, samples were randomly allocated. Microscopy analyses were conducted on images acquired in a random, unbiased manner.
Blinding	N/A

Reporting for specific materials, systems and methods

We require information from authors about some types of materials, experimental systems and methods used in many studies. Here, indicate whether each material, system or method listed is relevant to your study. If you are not sure if a list item applies to your research, read the appropriate section before selecting a response.

Materials & experimental systems

n/a	Involved in the study	
☐	☒	Antibodies
☐	☒	Eukaryotic cell lines
☒	☐	Palaeontology and archaeology
☐	☒	Animals and other organisms
☒	☐	Clinical data
☒	☐	Dual use research of concern
☒	☐	Plants

Methods

n/a	Involved in the study	
☒	☐	ChIP-seq
☒	☐	Flow cytometry
☒	☐	MRI-based neuroimaging

Antibodies

Antibodies used

All antibodies used in this study are detailed in the Methods sections:
- anti-PymTIP (1:500, rabbit polyclonal, PMID: 12456714)
- anti-PbUIS4 (1:500, goat polyclonal, AB0042-500, SIGEN)
- anti-mNeonGreen (1:400, mouse monoclonal 32F6, Proteintech)
- anti-Histon H3K9Ac (1:200, mouse monoclonal MABI0305, Gene Tex)
- anti- PvHSP70 (1:400, rabbit polyclonal, PMID: 25800544)
- anti-PyPABP1 (1:200, rabbit polyclonal, PMID: 29359180)
- anti -mCherry (1:400, rat monoclonal antibody (16D7), Invitrogen)
- donkey anti-mouse 488 (Invitrogen, Cat # A21202; RRID: AB_141607)
- donkey anti- mouse 594 (Invitrogen, Cat # A21203; RRID: AB_141633)
- donkey anti-rabbit 488 (Invitrogen, Cat # A-21206; RRID: AB_2532792)
- donkey anti-rabbit 594 (Invitrogen, Cat # A21207; RRID: AB_141637)
- donkey anti-rabbit 647 (Invitrogen, Cat # A-31573; RRID: AB_2536183)
- donkey anti-goat 594 (Invitrogen, Cat # A-11058; RRID: AB_2534105)
- donkey anti-goat 647 (Invitrogen, Cat # A-21447; RRID: AB_2535864)
- donkey anti-rat 594 (Invitrogen, Cat # A-21209; RRID: AB_2535795)

All the secondary antibodies were used at a dilution 1:1000

Validation

Non-commercially available antibodies were first validated individually as described below:
- anti-PymTIP (PMID: 12456714), on salivary gland sporozoites
- anti- PvHSP70 (PMID: 25800544), on liver stages
- anti-PyPABP1 (PMID: 29359180), on blood stages and salivary gland sporozoites

Eukaryotic cell lines

Policy information about cell lines and Sex and Gender in Research

Cell line source(s)	- HepG2-CD81 (orginally obtained from Olivier Silvie Laboratory, Cimi, Paris, France,)<br>- Primary Human Hepatocytes (BioIVT, batch #BGW)
Authentication	- HepG2-CD81: This cell line was originally obtained from the Olivier Silvie Laboratory (Cimi, Paris, France). No additional authentication was performed in our laboratory; however, the line was routinely monitored for morphology and growth characteristics.<br>-Primary Human Hepatocytes (BioIVT, batch #BGW, derived from a female donor): These primary cells were purchased directly from BioIVT. No further authentication was carried out beyond the provider's certificate of analysis, which includes donor information, viability, and quality-control testing.
Mycoplasma contamination	Both the HepG2-CD81 and the primary human hepatocytes cell lines tested negative for mycoplasma contamination.
Commonly misidentified lines (See ICLAC register)	N/A

Animals and other research organisms

Policy information about studies involving animals; ARRIVE guidelines recommended for reporting animal research, and Sex and Gender in Research

Laboratory animals	- Seven to eight weeks old female Swiss Webster (SW) mice were purchasing from Envigo Laboratory.<br>- Anopheles Stephensi mosquitoes were reared in the in-house Insectary. Four to six days old female mosquitoes were used to run the Plasmodium parasites' infection cycle.
Wild animals	N/A
Reporting on sex	Female
Field-collected samples	N/A
Ethics oversight	This study was carried out in accordance with the recommendations of the NIH Office of Laboratory Animal Welfare standards (OLAW welfare assurance #D16-00119). The mice were maintained under specific pathogen-free conditions with 12 h light/12 h dark cycle, 72°F temperature and 45% humidity at the Center for Global Infectious Disease Research, Seattle Children's Research Institute (SCRI). The protocols were approved by the Center for Infectious Disease Research Institutional Animal Care and Use Committee (IACUC) under Protocol SK00505 and SK00666 (for rodent malaria parasites).

Note that full information on the approval of the study protocol must also be provided in the manuscript.

Plants

Seed stocks	N/A
Novel plant genotypes	N/A
Authentication	N/A
