# Supplementary material for: Two Plasmodium vivax hypnozoite-expressed RNA-binding proteins inhibit liver stage replication
Source: Nat Commun. 2026 May 30;17:7048. doi: 10.1038/s41467-026-73666-0 (PMC13392028; doi:10.1038/s41467-026-73666-0)
Supplement: Supplementary file 6 — Transparent Peer Review file [file 41467_2026_73666_MOESM6_ESM.pdf]

nature portfolio

Peer Review File

Two Plasmodium vivax hypnozoite-expressed RNA-binding proteins inhibit liver stage replication

Corresponding Author: Professor Stefan Kappe

This file contains all reviewer reports in order by version, followed by all author rebuttals in order by version.

Version 0:

Reviewer comments:

Reviewer #1

(Remarks to the Author)
In this manuscript, the authors follow up on the hypothesis that two previously identified RNA-binding proteins are involved in regulation of liver stage dormancy in relapsing malaria parasites, including the human parasite P. vivax. In the study, they over-express the two candidates in liver stage parasites of the non-relapsing rodent malaria P. yoelii, for which a versatile transfection system has been developed. Such studies are currently not possible in P. vivax itself. The authors investigate the in vivo and in vitro phenotypes of the transgenic parasites and see that the liver stages stop growing and deteriorate in culture. Based on these data they conclude that both RNA-binding proteins, for which they also determined binding motifs, by binding to RNAs, inhibit liver stage growth and that this is thus one potential mechanism for hypnozoite formation.
While the studies have been carefully conducted, with many appropriate controls, and the work is of interest to a wide public, this reviewer thinks a vital control is missing, making it not possible to draw firm conclusions from the phenotypic analyses. I can easily imagine that massive over-expression of any RNA-binding protein in liver stages will result in stop of development and ultimately cell death, due to massive binding of mRNA needed for cell viability. This is then unrelated to hypnozoite formation and dormancy. An appropriate control for this would be to also over-express an unrelated RNA-binding protein and only if a completely different phenotype would be observed, the current conclusion may be valid. The use of a regulatable system and/or the use of a much weaker (or the native) promoter, may further strengthen the data set. Such studies are possible in P. yoelii which was used here because of its sophisticated transfection system. Without such additional data the current data are very hard to interpret and the current conclusion of the authors is very premature.

Specific minor comments
• Line 1: The title is somewhat misleading. Please add …….in a rodent malaria model
• Line 16, 69 and further in the text: This hypothesis has been published in ref 22, where also the 2 RNA-binding proteins were identified in P. cynomolgi hypnozoites. This should be acknowledged
• Line 60: P. cynomolgi, apart from a nonhuman primate malaria parasite, is also an emerging zoonosis
• Line 90: Ref 22 describes single cell transcriptomics of hypnozoites, not bulk
Textual:
• Line 62: 'enriched hypnozoites' should be 'enriched hypnozoite'
• Line 86: please delete 'for'
• Line 87: …proteins that are….
• Line 90: hypnozoites rather than hypnozoite
• Line 108: remains rather than remain
• Line 354: continuously
• Line 400: associations
• Sup Fig.1B: cynomolgi rather than cynamolgi

Reviewer #2

(Remarks to the Author)
The manuscript entitled "Two Plasmodium vivax hypnozoite-expressed RNA-binding proteins inhibit liver stage replication" by Vo et al. examines expression of Initiation of Exo-erythrocytic Schizogony Inhibited (IESI) proteins during P. vivax liver stages. Due to experimental constraints with P. vivax, the study used the rodent parasite P. yoelii for the functional analysis

of IESIs. The data generally supports the claims in the title and abstract.

Key findings include:

IESI expression is restricted to hypnozoite
Using RNA-FISH and transcriptomic analyses, the study demonstrates that IESI-1 and IESI-2 are highly expressed in hypnozoites, while their expression is not detected in schizonts. This expression pattern highlights the specific association of these RNA-binding proteins with the dormant liver stage of P. vivax. Immunofluorescence studies of overexpressed IESI transgenes in the P. yoleii show that both IESI-1 and IESI-2 co-localize with the stress granule marker PABP1. This co-localization suggests that these proteins are involved in translational repression possibly contributing to the maintenance of hypnozoite dormancy.

Functional characterization of IESI in Liver Stages
Functional studies involving overexpression of these proteins in the liver stages of P. yoelii revealed significant phenotypic effects. Specifically, their overexpression led to growth arrest, minimal nuclear replication, and a marked delay in the patency of liver to blood-stage transition. These results suggest a potent inhibitory role during liver-stage development. The effects of IESI-1 and IESI-2 are specific to the liver stage, as no impact was observed on either blood or mosquito stages.

Determination of IESI RNA-Binding sequences
RNA Bind-N-Seq (RBNS) experiments further established the RNA-binding motifs recognized by these proteins. IESI-1 preferentially binds to the UGAUGA motif, while IESI-2 binds to the UGACAC motif. Both motifs are notably enriched within the liver-stage transcriptomes, indicating a potential regulatory role in this developmental phase.

A significant weakness of the study is the limitation of functional characterization of IESIs to overexpression in a non-relapsing parasite. While it poses challenges, it seems like an acceptable proxy system given the experimental constraints of relapsing parasites, but the authors should address this major limitation more explicitly in the discussion. Despite this limitation, these findings enhance our knowledge of dormancy in vivax malaria, an underexplored area vital for developing new treatments. There are, however, a few major points that I would like to see addressed and discussed before publication.

Major comments:

1.While the authors demonstrate that UIS4-driven PvIESIs overexpression does not influence blood or mosquito stages, employing mNeon as a negative control alone may not adequately establish the specificity of effects observed during the liver stage. The manuscript would be strengthened by confirming the specificity through mutation or deletion of critical regions, such as the RNA binding domain. At minimum, overexpressing an unrelated RNA binding protein—one that does not elicit similar liver stage phenotypes—should be considered to rule out that the effects seen are not just the results of some non-specific “toxicity” of RNA binding proteins overexpression. Have the authors explored the possibility of overexpressing the Pb or Pf orthologs? This approach may also enable structure/function analysis by constructing chimeras to pinpoint the essential regions of Pv IESI proteins involved in inhibiting schizogony.

2.The authors establish that overexpressing Py parasites are arrested, small and show longer patency periods. It would be useful to compare the global transcriptome of those arrested Py schizonts to normal schizonts. What are the genes that are specifically inhibited in the arrested Py parasites? Can we still detect some of the early schizonts markers (e.g. LISP2) in those arrested parasites, which of the late schizonts genes are repressed? Any relationship with the genes identified as possible targets in Pv containing the putative RNA binding sites identified?

3.Notably, dormancy in hypnozoites is a reversible phase, and it remains uncertain whether the arrested Py parasites overexpressing IESIs are simply non-viable or retain the ability to resume growth. While I acknowledge the technical challenges associated with generating regulatable transgenes, if this cannot be addressed experimentally, it is important that claims regarding the resemblance of these phenotypes in Py to Pv dormancy are appropriately moderated in the results and discussion sections.

Minor comments:

1.Providing an analysis of the structural similarities and differences in IESIs between relapsing parasites (P. vivax, P. ovale, and P. cynomolgi) and non-relapsing parasites (P. falciparum, P. malariae, and P. knowlesi) would be beneficial.

2.There is a missing section to the sentence between line 271-272 starting with “Because motif localization….”

3.Another defining feature of dormancy is its reversibility and this should be mentioned in discussion line 335-337

4.Line 406- 413, the authors suggest that differences in structure, expression pattern, and/or subcellular localization may account for functional differences between Pv IESIs and their non-relapsing Plasmodium orthologs. They should include data on expression patterns and subcellular localization of IESIs orthologs in the Py life cycle.

5.Figure 8 model is speculative, as the manuscript does not contain direct data supporting it. It would be more appropriate in a review article or an opinion piece, and I would recommend removing it from the manuscript.

6.There are typos throughout the manuscript (which I appreciate as it is clear proof of authenticity) that should be corrected. I passed on some of those to the editor privately and they will share it with you prior to the final proof review.

Version 1:

Reviewer comments:

Reviewer #1

(Remarks to the Author)
The authors have carefully considered and replied to my queries. Importantly, they followed my suggestion to overexpress an unrelated RNA-binding protein (in this case PUF2 of P. vivax) and noted no phenotypic changes as compared to wild-type liver stage development. This strongly suggests that the effects seen with over-expression of IESI-1 and 2 must be due to the specific proteins. Whether the effects are reversible, important to know since true hypnozoites can reactivate, apparently cannot be tested experimentally in Py. Nonetheless, the additional data provided have strengthened the original conclusions.

Reviewer #2

(Remarks to the Author)
The authors have made significant improvements to the manuscript and addressed the primary concerns raised during review. However, I still find Figure 9 to be somewhat misleading because the images depict parasites not used in this study. I acknowledge that this is mainly an editorial decision, and if the editor believes it is an effective use of space, I would not oppose publication in its current form. Additionally, I agree with the other reviewer that adding clarification about the use of a rodent malaria model in the title would be appropriate.

Overall, I appreciate the authors' meaningful contribution to research on relapsing malaria and I recommend publication.

Open Access This Peer Review File is licensed under a Creative Commons Attribution 4.0 International License, which permits use, sharing, adaptation, distribution and reproduction in any medium or format, as long as you give appropriate credit to the original author(s) and the source, provide a link to the Creative Commons license, and indicate if changes were made.
In cases where reviewers are anonymous, credit should be given to 'Anonymous Referee' and the source.
The images or other third party material in this Peer Review File are included in the article’s Creative Commons license, unless indicated otherwise in a credit line to the material. If material is not included in the article’s Creative Commons license and your intended use is not permitted by statutory regulation or exceeds the permitted use, you will need to obtain permission directly from the copyright holder.
To view a copy of this license, visit https://creativecommons.org/licenses/by/4.0/

Author Responses to Reviewer's Comments (Point-by-Point)

We greatly appreciate all of the reviewers' comments and suggestions and thank them for helping us to improve our manuscript.

Below you can find our detailed response for all points raised by the reviewers.

REVIEWER COMMENTS

Reviewer #1 (Remarks to the Author):

In this manuscript, the authors follow up on the hypothesis that two previously identified RNA-binding proteins are involved in regulation of liver stage dormancy in relapsing malaria parasites, including the human parasite P. vivax. In the study, they over-express the two candidates in liver stage parasites of the non-relapsing rodent malaria P. yoelii, for which a versatile transfection system has been developed. Such studies are currently not possible in P. vivax itself. The authors investigate the in vivo and in vitro phenotypes of the transgenic parasites and see that the liver stages stop growing and deteriorate in culture. Based on these data they conclude that both RNA-binding proteins, for which they also determined binding motifs, by binding to RNAs, inhibit liver stage growth and that this is thus one potential mechanism for hypnozoite formation.
While the studies have been carefully conducted, with many appropriate controls, and the work is of interest to a wide public, this reviewer thinks a vital control is missing, making it not possible to draw firm conclusions from the phenotypic analyses. I can easily imagine that massive over-expression of any RNA-binding protein in liver stages will result in stop of development and ultimately cell death, due to massive binding of mRNA needed for cell viability. This is then unrelated to hypnozoite formation and dormancy. An appropriate control for this would be to also over-express an unrelated RNA-binding protein and only if a completely different phenotype would be observed, the current conclusion may be valid. The use of a regulatable system and/or the use of a much weaker (or the native) promoter, may further strengthen the data set. Such studies are possible in P. yoelii which was used here because of its sophisticated transfection system. Without such additional data the current data are very hard to interpret and the current conclusion of the authors is very premature.

We thank the reviewer for this thoughtful and constructive critique. We agree that distinguishing protein-specific functions and phenotypes from unspecific phenotypes that might be caused by expression of any RNA-binding protein is important. We thus have addressed this issue by performing the additional control experiment suggested by the reviewer. We expressed the RNA binding protein PUF2 of *P. vivax,* using the same expression cassette that we used for IESI-1 and IESI-2, in *P. yoelii* and show that this does not cause arrest of liver stage replication. This substantiates our findings and strongly supports our conclusions regarding the specific function(s) of IESI-1 and IESI-2. The data is included in the revised manuscript results section and shown in Figure 6 and supplemental Figure 5:

"Overexpression of the RNA-binding protein PUF2 does not induce liver-stage arrest

To address the possibility that overexpression of any Plasmodium RNA-binding protein in liver stages could nonspecifically arrest parasite development, potentially through excessive

sequestration of mRNAs required for parasite viability, we performed a control experiment using an RNA-binding protein unrelated to IESI-1 and IESI-2. We selected PvPUF2, a well-characterized member of the PUF family containing a conserved PUM domain[42-44]. Bulk transcriptomic data from Gural et al.[17] show that PvPUF2 transcripts were upregulated in hypnozoites relative to schizonts (Figure S5A), indicating that this RNA-binding protein is naturally expressed during liver-stage development. We confirmed this expression pattern by RNA-FISH analysis, which detected PvPUF2 transcripts in both hypnozoites and schizonts (Figure 6A). Using the same strategy applied for PvIESI-1 and 2, we generated a transgenic line overexpressing PvPUF2 fused to mCherry under the control of the UIS4 promoter (Figure S5B) and confirmed correct genomic integration (Figure S5C). Overexpression of PvPUF2 had no observable impact on blood stage development (Figure S5D), gametocytogenesis (Figure S5E), oocyst formation (Figure S5F) or sporogony (Figure S5G). UIS4 promoter control ascertained PvPUF2 protein expression throughout liver-stage development, which was visualized using antibodies to mCherry (Figure 6B). Yet, despite robust liver stage expression of PvPUF2, parasite growth and maturation were indistinguishable from the PyXNL control, with comparable liver-stage parasite sizes 44 hours post infection (Figure 6C). Consistent with normal liver stage progression, patent blood-stage infection occurred three days after sporozoite inoculation, with no delay relative to control (Table S1). These results show that overexpression of any RNA-binding protein does not inevitably impair parasite liver stage development, further ascertaining the specificity of the phenotype observed for PvIESI-1 and 2 expressions."

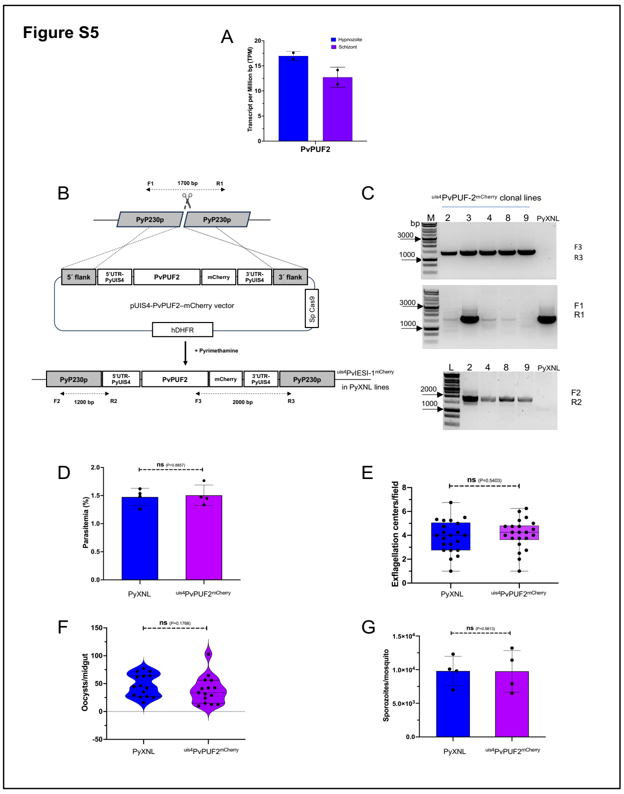

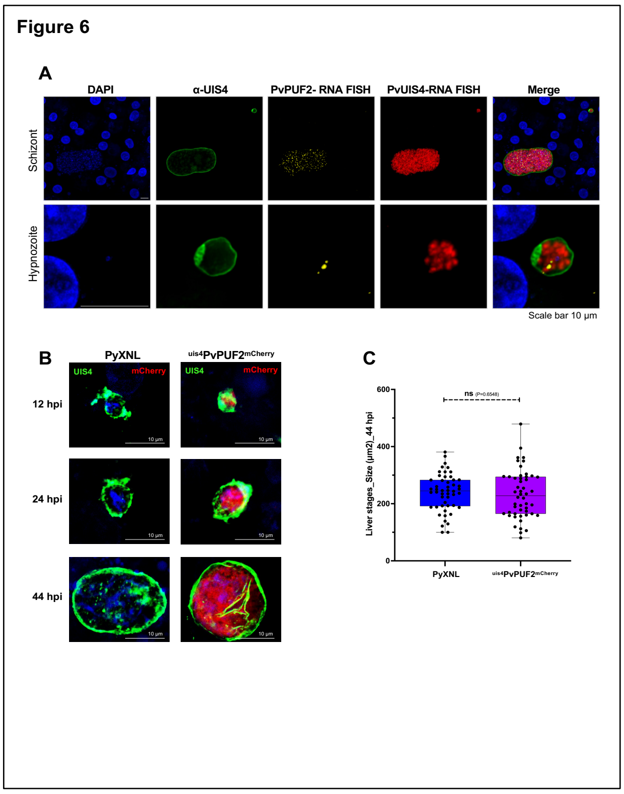

Specific minor comments:

We thank the reviewer for the careful reading of our manuscript and the helpful suggestions. We have addressed each point as detailed below.

• Line 1: The title is somewhat misleading. Please add …….in a rodent malaria model
Adding this would exceed allowed title length. Also, the use of a rodent malaria model is first mentioned in the abstract and it is thus clear what model we use. We prefer to maintain the title as is.

• Line 16, 69 and further in the text: This hypothesis has been published in ref 22, where also the 2 RNA-binding proteins were identified in P. cynomolgi hypnozoites. This should be acknowledged

We thank the reviewer for pointing this out. We have now explicitly acknowledged Ref 22 in Lines 69 (and where appropriate later in the text), clarifying that the two RNA-binding proteins were identified in *P. cynomolgi* hypnozoites in that study. These updates have been incorporated into the Introduction section as detailed below:

"…This analysis identified two putative RNA-binding proteins expressed in *Pv* hypnozoites, with the orthologs of these proteins also previously identified as potential candidates regulating hynozoite dormancy in *P.cynomolgi*[22]."

• Line 60: P. cynomolgi, apart from a nonhuman primate malaria parasite, is also an emerging zoonosis

We agree. The text has been revised to indicate that *P. cynomolgi* is not only a nonhuman primate malaria parasite but also an emerging zoonotic infection in humans.

• Line 90: Ref 22 describes single cell transcriptomics of hypnozoites, not bulk

Thank you for this correction. We have revised the text to accurately state that Ref 22 reports single-cell transcriptomic analysis of hypnozoites rather than bulk transcriptomics.

Textual:

We appreciate the reviewer's careful attention to detail. The following corrections have been made:

• Line 62: 'enriched hypnozoites' should be 'enriched hypnozoite'
• Line 86: please delete 'for'
• Line 87: …proteins that are….
• Line 90: hypnozoites rather than hypnozoite
• Line 108: remains rather than remain
• Line 354: continuously
• Line 400: associations
• Sup Fig.1B: cynomolgi rather than cynamolgi

Reviewer #2 (Remarks to the Author):

The manuscript entitled "Two Plasmodium vivax hypnozoite-expressed RNA-binding proteins inhibit liver stage replication" by Vo et al. examines expression of Initiation of Exo-erythrocytic Schizogony Inhibited (IESI) proteins during P. vivax liver stages. Due to experimental

constraints with P. vivax, the study used the rodent parasite P. yoelii for the functional analysis of IESIs. The data generally supports the claims in the title and abstract.

Key findings include:

IESI expression is restricted to hypnozoite Using RNA-FISH and transcriptomic analyses, the study demonstrates that IESI-1 and IESI-2 are highly expressed in hypnozoites, while their expression is not detected in schizonts. This expression pattern highlights the specific association of these RNA-binding proteins with the dormant liver stage of P. vivax. Immunofluorescence studies of overexpressed IESI transgenes in the P. yoleii show that both IESI-1 and IESI-2 co-localize with the stress granule marker PABP1. This co-localization suggests that these proteins are involved in translational repression possibly contributing to the maintenance of hypnozoite dormancy.

Functional characterization of IESI in Liver Stages
Functional studies involving overexpression of these proteins in the liver stages of P. yoelii revealed significant phenotypic effects. Specifically, their overexpression led to growth arrest, minimal nuclear replication, and a marked delay in the patency of liver to blood-stage transition. These results suggest a potent inhibitory role during liver-stage development. The effects of IESI-1 and IESI-2 are specific to the liver stage, as no impact was observed on either blood or mosquito stages.

Determination of IESI RNA-Binding sequences
RNA Bind-N-Seq (RBNS) experiments further established the RNA-binding motifs recognized by these proteins. IESI-1 preferentially binds to the UGAUGA motif, while IESI-2 binds to the UGACAC motif. Both motifs are notably enriched within the liver-stage transcriptomes, indicating a potential regulatory role in this developmental phase.

A significant weakness of the study is the limitation of functional characterization of IESIs to overexpression in a non-relapsing parasite. While it poses challenges, it seems like an acceptable proxy system given the experimental constraints of relapsing parasites, but the authors should address this major limitation more explicitly in the discussion. Despite this limitation, these findings enhance our knowledge of dormancy in vivax malaria, an underexplored area vital for developing new treatments. There are, however, a few major points that I would like to see addressed and discussed before publication.

We thank the reviewer for raising this point. We acknowledge that overexpression in a non-relapsing parasite cannot fully capture *Plasmodium vivax* biology, including hypnozoite formation and activation. This limitation has now been explicitly noted in the “Limitation of the study” section following the Discussion section.

“Limitation of the study

A key limitation of this study is that our functional characterization of IESI-1 and 2 relied on overexpression in a rodent malaria model, which cannot fully capture the biology of *Pv* liver stages, particularly hypnozoite persistence, and activation. However, given the current technical barriers to continuous culture and genetic manipulation of *Pv*, the experimental

model we used provides a clear and tractable framework for functional interrogation of proteins that might regulate hypnozoite biology. Accordingly, our conclusions are constrained by the model used and should not be considered definitive for relapsing parasites. Despite these limitations, our findings provide a potential key mechanism by which malaria parasites can enter a non-replicating state in the liver and informs our understanding of dormancy in *Pv*. Integration of our functional data with RBNS-derived RNA targets and published liver-stage transcriptomes reveals consistent enrichment of genes involved in translational control, cellular quiescence, and early liver stage development. This convergence supports a model in which IESI-1 and IESI-2 regulate post-transcriptional networks linked to parasite dormancy and provides a framework for future validation in relapsing malaria parasites.

Major comments:

1.While the authors demonstrate that UIS4-driven PvIESIs overexpression does not influence blood or mosquito stages, employing mNeon as a negative control alone may not adequately establish the specificity of effects observed during the liver stage. The manuscript would be strengthened by confirming the specificity through mutation or deletion of critical regions, such as the RNA binding domain. At minimum, overexpressing an unrelated RNA binding protein—one that does not elicit similar liver stage phenotypes—should be considered to rule out that the effects seen are not just the results of some non-specific “toxicity” of RNA binding proteins overexpression.

We thank the reviewer for this thoughtful and constructive critique. We agree that distinguishing protein-specific functions and phenotypes from unspecific phenotypes that might be caused by expression of any RNA-binding protein is important. We thus have addressed this issue by performing the additional control experiment suggested by the reviewer. We expressed the RNA binding protein PUF2 of *P. vivax,* using the same expression cassette that we used for IESI-1 and IESI-2 in *P. yoelii* and show that this does not cause arrest of liver stage replication. This substantiates our findings and strongly supports our conclusions regarding the specific function(s) of IESI-1 and IESI-2. The data is included in the revised manuscript results section and shown in Figure 6 and supplemental Figure 5:

“Overexpression of the RNA-binding protein PUF2 does not induce liver-stage arrest

To address the possibility that overexpression of any Plasmodium RNA-binding protein in liver stages could nonspecifically arrest parasite development, potentially through excessive sequestration of mRNAs required for parasite viability, we performed a control experiment using an RNA-binding protein unrelated to IESI-1 and IESI-2. We selected PvPUF2, a well-characterized member of the PUF family containing a conserved PUM domain[42-44]. Bulk transcriptomic data from Gural et al.[17] show that PvPUF2 transcripts were upregulated in hypnozoites relative to schizonts (Figure S5A), indicating that this RNA-binding protein is naturally expressed during liver-stage development. We confirmed this expression pattern by RNA-FISH analysis, which detected PvPUF2 transcripts in both hypnozoites and schizonts

(Figure 6A). Using the same strategy applied for PvIESI-1 and 2, we generated a transgenic line overexpressing PvPUF2 fused to mCherry under the control of the UIS4 promoter (Figure S5B) and confirmed correct genomic integration (Figure S5C). Overexpression of PvPUF2 had no observable impact on blood stage development (Figure S5D), gametocytogenesis (Figure S5E), oocyst formation (Figure S5F) or sporogony (Figure S5G). UIS4 promoter control ascertained PvPUF2 protein expression throughout liver-stage development, which was visualized using antibodies to mCherry (Figure 6B). Yet, despite robust liver stage expression of PvPUF2, parasite growth and maturation were indistinguishable from the PyXNL control, with comparable liver-stage parasite sizes 44 hours post infection (Figure 6C). Consistent with normal liver stage progression, patent blood-stage infection occurred three days after sporozoite inoculation, with no delay relative to control (Figure 6D). These results show that overexpression of any RNA-binding protein does not inevitably impair parasite liver stage development, further ascertaining the specificity of the phenotype observed for PvIESI-1 and 2 expressions."

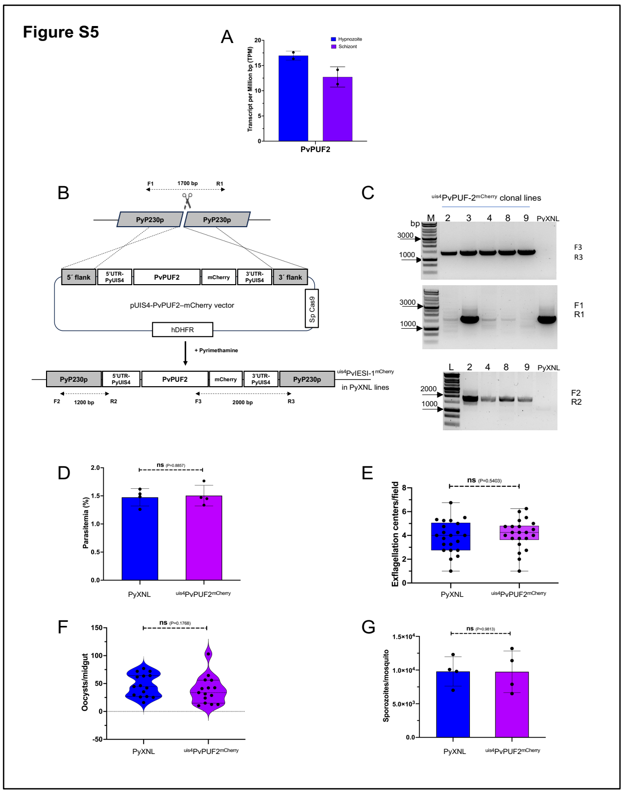

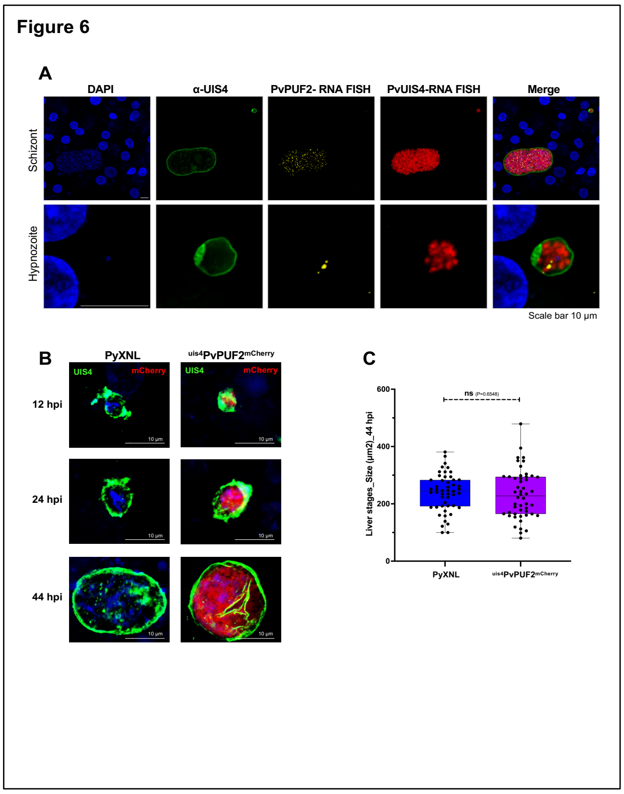

Have the authors explored the possibility of overexpressing the Pb or Pf orthologs? This approach may also enable structure/function analysis by constructing chimeras to pinpoint the essential regions of *Pv* IESI proteins involved in inhibiting schizogony.

We thank the reviewer for this suggestion. We have not yet attempted overexpression of the *P. berghei* or *P. falciparum* orthologs and while a detailed structure/function analysis of IESI-1 and IESI-1 is planned, we feel it is beyond the scope of this study.

We agree that cross-species overexpression and chimeric approaches could provide valuable structure–function insights. We have acknowledged it as an important direction for future investigation in the Discussion section below:

" If functional differences reside within the protein sequences, future overexpression studies of IESI-1 and IESI-2 orthologs from non-relapsing parasites, such as *Pf,* in *Py* and the design of chimeric proteins might reveal protein regions that are specifically involved in inducing a non-replicative state in liver stages."

2.The authors establish that overexpressing Py parasites are arrested, small and show longer patency periods. It would be useful to compare the global transcriptome of those arrested Py schizonts to normal schizonts. What are the genes that are specifically inhibited in the arrested Py parasites? Can we still detect some of the early schizonts markers (e.g. LISP2) in those arrested parasites, which of the late schizonts genes are repressed? Any relationship with the genes identified as possible targets in Pv containing the putative RNA binding sites identified?

We thank the reviewer for this suggestion. We fully agree that a comparative global transcriptomic analysis between arrested and normally developing liver-stages would provide valuable insights into the genes and pathways affected by PvIESI-1 and 2 overexpression. However, we are currently unable to conduct such an experiment due to technical limitations. Specifically, the PvIESI-1 and 2–mNeon signals do not allow for fluorescence-based enrichment or isolation of the overexpressing parasite population. Consequently, we are unable to obtain sufficient and well-defined parasite material to support robust RNA sequencing analysis of the arrested schizonts.

Despite this limitation, we sought to address the reviewer's question through complementary approaches. Our RBNS analysis identified a set of candidate RNA targets and associated molecular pathways potentially regulated by IESI-1 and 2, many of which are implicated in liver-stage growth and schizogony. Furthermore, integration of these findings with published hypnozoite-enriched transcriptomic datasets provides indirect but supportive evidence for the types of genes and pathways that may be selectively repressed in PvIESI-1 and 2–overexpressing parasites. While this does not replace a direct transcriptomic comparison, it offers valuable insights consistent with the observed developmental arrest phenotype,

although the mechanistic insights require further validation, as discussed in the Discussion section.

3.Notably, dormancy in hypnozoites is a reversible phase, and it remains uncertain whether the arrested Py parasites overexpressing IESIs are simply non-viable or retain the ability to resume growth. While I acknowledge the technical challenges associated with generating regulatable transgenes, if this cannot be addressed experimentally, it is important that claims regarding the resemblance of these phenotypes in Py to Pv dormancy are appropriately moderated in the results and discussion sections.

We thank the reviewer for this important point. We agree that it remains unclear whether the arrested *Py* parasites overexpressing IESI-1 or 2 are non-viable or retain the ability to resume growth. Given the current technical limitations in generating transgenes that can be exquisitely regulated in liver stages, we cannot directly address reversibility. We have therefore moderated our statements in the Results and Discussion to indicate that the developmental arrest resembles aspects of *Pv* dormancy, without implying confirmed functional equivalence.

Minor comments:

1.Providing an analysis of the structural similarities and differences in IESIs between relapsing parasites (P. vivax, P. ovale, and P. cynomolgi) and non-relapsing parasites (P. falciparum, P. malariae, and P. knowlesi) would be beneficial.

We agree that the structure and sequence conservation between relapsing and non-relapsing parasite species is of immediate interest. We have expanded upon the phylogenetic analysis included in the original submission (Figures S1C, D) in the text of the results (line 130-140). This expanded analysis demonstrates that the RNA-binding domains of IESI-1 and IESI-2 are highly conserved across both relapsing (*P. vivax*, *P. cynomolgi*, *P. ovale*) and non-relapsing (*P. falciparum*, *P. yoelii*, *P. knowlesi*, *P. malariae*) species, with >95% identity in the RRM domain of IESI-1 and >98% identity in the CCCH zinc finger domains of IESI-2. In contrast, regions outside the RNA-binding domains show substantial sequence divergence between species. This sequence divergence is also apparent in the AlphaFold predicted secondary and tertiary structures of PvIESI-1 and 2 compared to PyIESI-1 and 2 (shown below). In fact, the AlphaFold algorithm was only able to confidently predicted regions (pIDDT>70) of the protein structures were those corresponding to the RNA binding domains themselves (shown below). These findings suggest that while the RNA-binding function is conserved, species-specific regulatory mechanisms may determine the distinct roles of these proteins in liver stage development. The text has been modified as follows:

“Phylogenetic analysis showed that both IESI-1 and IESI-2 orthologues are conserved across the *Plasmodium* genus (Figures S1C, S1D). Their full sequences are conserved among Asian malaria species (Figures S1C, S1D), though the *P. cynomolgi* IESI-1 orthologue is significantly

shorter (1078 aa vs 1865 aa in *P. vivax*), sharing only the C-terminal region (Figure S1C). For both proteins, the RNA-binding domains are highly conserved among divergent *Plasmodium* species (Figures S1C, S1D), with the *Pv* and *Py* orthologs (PY17X_0911200 for IESI-1; PY17XNL_000504404 for IESI-2) sharing 95% and 98% identity in their RRM and CCCH domains, respectively. In fact, for IESI-2, the N-terminal zinc finger domains are the only regions of sequence conservation outside of the Asian *Plasmodium* parasite species, with sequence identity >70% for all *Plasmodium* orthologues (Figures S1C, S1D). Comparatively, the full-length protein sequence alignments indicate substantial similarity between PvIESI-1 or PvIESI-2 and their corresponding orthologues in *P. cynomolgi*, at 85% and 74%, respectively, while sequence similarity with orthologs from other *Plasmodium* species range between 45% and 60% (Figure S1C, S1D)."

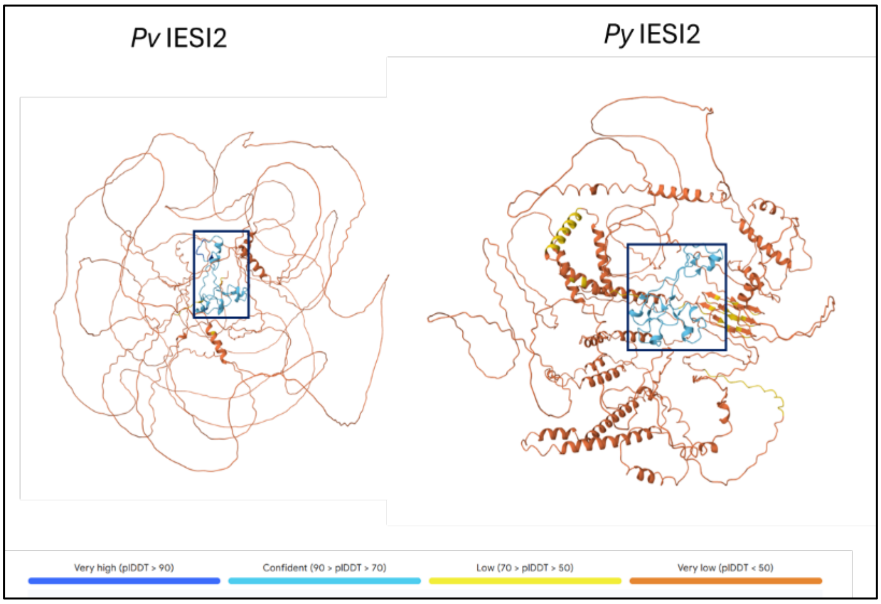

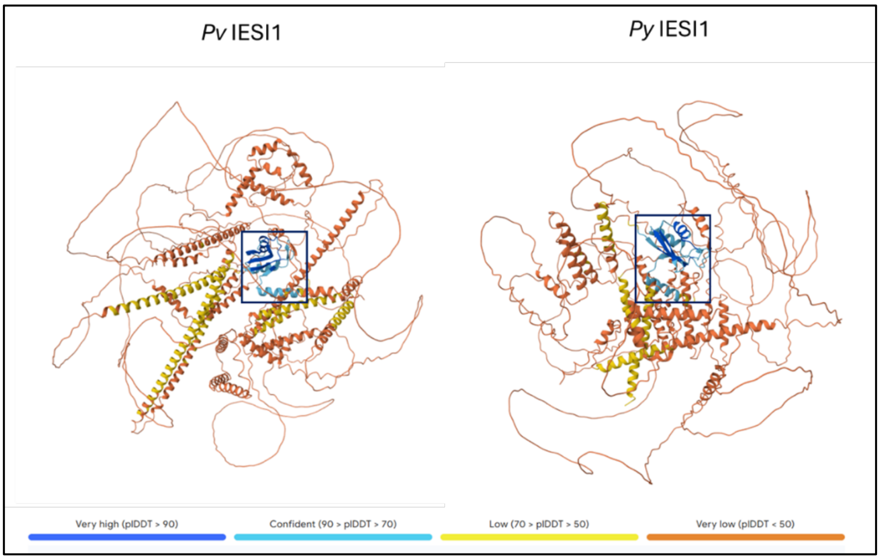

For future studies, we plan to conduct a more comprehensive structural analysis of these proteins. Such work is expected to provide mechanistic insights into the differences between relapsing and non-relapsing parasites and yield valuable functional information. We have incorporated this point into the Discussion section as follows:

"...The comparison of IESI-1 and IESI-2 full-length protein sequences indicated that the *Pv* orthologs are genetically closer to *P. cynomolgi* than to othologs in other *Plasmodium* species. This genetic proximity is markedly distinct from the human parasite *P. ovale*, which also forms hypnozoites[54,55]. If distinct biological functions reside in IESI-1 and IESI-2 protein regions unique to *Pv* and *P.cynomolgi*, the molecular mechanisms or conserved pathways regulating hypnozoite dormancy are more likely shared between *Pv* and *P. cynomolgi* rather than between *Pv* and *P. ovale.* Future studies examining the structural similarities and differences of IESI-1 and IESI-2 between relapsing and non-relapsing parasites might identify protein regions that mediate species-specific functions and reveal adaptations associated with hypnozoite formation and this will help to refine structure/function analysis of these proteins.."

2.There is a missing section to the sentence between line 271-272 starting with "Because motif localization…."

We thank the reviewer for catching this. We have revised the sentence to ensure it reads fully and clearly, completing the thought regarding motif localization in line 434-436: "Because motif localization within transcripts can indicate functional significance, we examined the distribution of 5'-UGAUGA-3' motifs, which was enriched in exonic regions,…".

3.Another defining feature of dormancy is its reversibility and this should be mentioned in discussion line 335-337

We agree that reversibility is a key feature of hypnozoite dormancy. We have added a statement in the Discussion to acknowledge that hypnozoite dormancy is reversible, highlighting the uncertainty regarding whether arrested *Py* parasites overexpressing IESIs retain the ability to resume growth. The added text is provided below:

"An important aspect of hypnozoite biology is reversibility of the non-replicative state (activation)[7,49], which allows hypnozoites to enter schizogony. However, whether the declining expression of PvIESI-1 and PvIESI-2 in parasites is mechanistically linked to entry into schizogony cannot be ascertained from these observations and requires further study."

4.Line 406- 413, the authors suggest that differences in structure, expression pattern, and/or subcellular localization may account for functional differences between Pv IESIs and their non-relapsing Plasmodium orthologs. They should include data on expression patterns and subcellular localization of IESIs orthologs in the Py life cycle.

We appreciate this suggestion and have incorporated the available information on the expression patterns of IESI orthologs across the *Py and Pf* life cycles, as included in the Discussion section:

Differences in expression profiles might also contribute to the distinct phenotypic effects of these proteins across parasite life cycle stages. Transcriptomic datasets available for PvIESI-1 and 2 showed preferential expression of either protein in hypnozoites compared to liver schizonts (Figure 1A), but there was also evidence for blood stage expression of PvIESI-1 and 2 in patient isolates[56]. In *Py* and *Pf,* many more transcriptomic datasets are available for comparison of expression across the life cycle. In PyIESI-2 transcripts were expressed at higher levels in blood stages than in salivary gland sporozoites (Figure S7A), while IESI-1 displayed a similar expression pattern but with significantly lower transcript levels (Figure S7A). This pattern is consistent with previously published microarray datasets[31], which similarly show that PyIESI-2 transcripts are predominantly expressed during blood-stage development, with minimal expression detected in sporozoite and liver stages (Figure S7B), whereas IESI-1 consistently exhibits negligible transcript levels throughout the parasite life cycle (Figure S7B). In *Pf,* PfIESI-1 transcripts were expressed most highly in salivary gland sporozoites[57], though the transcript is also well-detected in ring and ookinete stages[58] (Figure S7C). Conversely, the PfIESI-2 transcript was most highly expressed in oocyst stages[57] but was also well-detected in late-stage gametocytes and ookinetes[59] (Figure S7D).

Figure S7

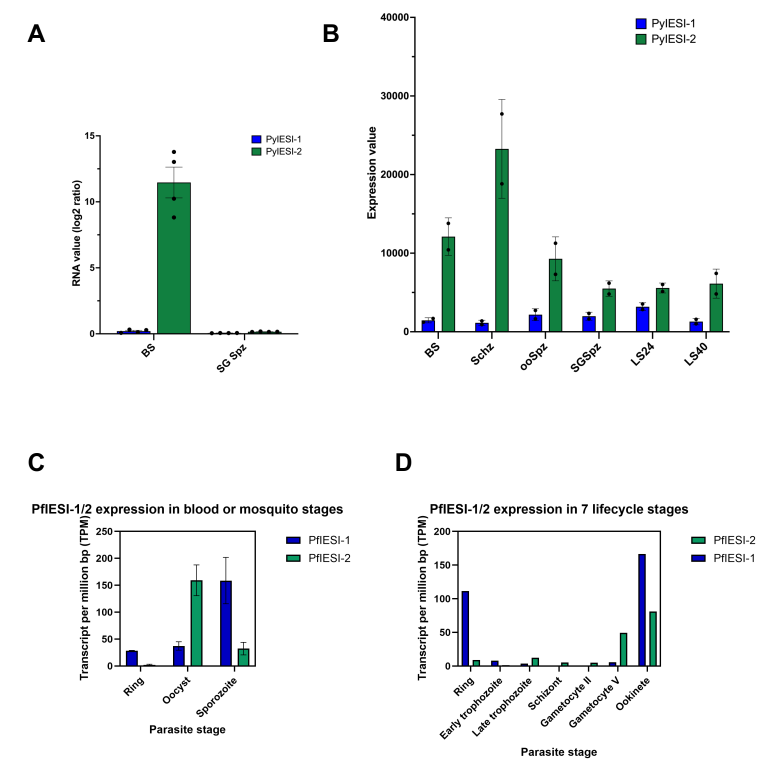

In addition, we plan to generate endogenously tagged PyIESI-1 and 2 parasites, but this will take time and we are not able to provide subcellular localization data at this time. We added this research direction to the Discussion section a shown below:

“These different expression patterns suggest that while these orthologous proteins bind the same RNA motifs cross Plasmodium species, their regulatory control has diverged. Relapsing species may have evolved altered expression of genes controlling parasite replication, potentially enabling these proteins to function in hypnozoite formation and maintenance. Future studies will characterize the endogenous expression and subcellular localization of these proteins in Py, Pb, and Pf, assessing whether translational regulation is conserved or has diverged among species with different liver stage developmental strategies.”

5. Figure 8 model is speculative, as the manuscript does not contain direct data supporting it. It would be more appropriate in a review article or an opinion piece, and I would recommend removing it from the manuscript.

We respectfully disagree. Figure 8 (now figure 9) frames a hypothesis that has been conceived based on our current data. We showed that IESI-1 and IESI-2 inhibit liver stage development when overexpressed in P. yoelii. IESI-1 and IESI-2 transcripts are expressed in P. vivax hypnozoites but not in schizonts. We think that this is good support for our hypothesis that these proteins might therefore be key regulators of hypnozoite formation through a major suppression of parasite replication. We find this useful as it will guide future experiments. We feel that the figure is a informative visual addition to our manuscript and enhances the manuscripts findings for the reader. We have described the proposed model in the Discussion text in a more cautions manner. The revised text reads:

“In summary, our study demonstrates that PvIESI-1 and PvIESI-2 are important suppressors of parasite replication, likely via modulation of post-transcriptional repression of transcripts that encode proteins required for schizogony. This suggests a working model in which elevated expression and RNA-binding activity of PvIESI-1 and PvIESI-2 promote hypnozoite formation. Conversely, reduced expression might relieve this repression, allowing parasites to exit dormancy and enter schizogony. In established hypnozoites, subsequent changes in PvIESI-1 and PvIESI-2 abundance could be associated with activation, leading to secondary schizogony and potential blood-stage relapse (as illustrated in Figure 9)”.

6.There are typos throughout the manuscript (which I appreciate as it is clear proof of authenticity) that should be corrected. I passed on some of those to the editor privately and they will share it with you prior to the final proof review.

We appreciate the reviewer’s note, and we have carefully corrected all typos identified by the editor, as well as performed an additional thorough review to improve clarity and readability.

Finally, we sincerely thank all reviewers for their thoughtful and constructive feedback, and we believe that the revisions and clarifications provided have substantially strengthened the manuscript and improved its clarity.

Revision #2

REVIEWERS' COMMENTS

Reviewer #1 (Remarks to the Author):

The authors have carefully considered and replied to my queries. Importantly, they followed my suggestion to overexpress an unrelated RNA-binding protein (in this case PUF2 of P. vivax) and noted no phenotypic changes as compared to wild-type liver stage development. This strongly suggests that the effects seen with over-expression of IESI-1 and 2 must be due to the specific proteins. Whether the effects are reversible, important to know since true hypnozoites can reactivate, apparently cannot be tested experimentally in Py. Nonetheless, the additional data provided have strengthened the original conclusions.

We thank the reviewer for the careful reassessment of our manuscript and for recognizing the importance of the additional experiments. As noted, overexpression of the unrelated RNA-binding protein PUF2 from Plasmodium vivax did not result in detectable phenotypic changes compared to wild-type liver-stage development, supporting the specificity of the effects observed for IESI-1 and IESI-2.

We agree that assessing reversibility would be highly informative in the context of hypnozoite biology. However, as correctly pointed out, this cannot currently be addressed in the *Plasmodium yoelii* model due to the absence of an experimental system for reactivation. This limitation is now clearly stated in the revised manuscript and highlighted as an important direction for future studies in systems that more faithfully model *Plasmodium vivax* malaria relapse.

We appreciate the reviewer’s constructive feedback and are encouraged that the additional data strengthen our conclusions.

Reviewer #2 (Remarks to the Author):

The authors have made significant improvements to the manuscript and addressed the primary concerns raised during review. However, I still find Figure 9 to be somewhat misleading because the images depict parasites not used in this study. I acknowledge that this is mainly an editorial decision, and if the editor believes it is an effective use of space, I would not oppose publication in its current form. Additionally, I agree with the other reviewer that adding clarification about the use of a rodent malaria model in the title would be appropriate.

Overall, I appreciate the authors' meaningful contribution to research on relapsing malaria and I recommend publication.

We thank the reviewer for the careful evaluation of our revised manuscript and for acknowledging the improvements made.

Regarding Figure 9, we appreciate the concern that the schematic includes representative images not directly derived from this study. As this figure is intended to provide a conceptual summary of hypnozoite-like biology, we have now clarified in the figure legend that the images

are illustrative and not generated from the experimental system used here. As noted by the reviewer, this is largely an editorial matter, and the editor has agreed to retain the figure in its current form as an effective summary.

Similarly, with regard to the title, the editor has decided to retain the current wording without modification following discussion.

Finally, we sincerely thank the reviewers for their time, rigorous evaluation of our manuscript, and highly constructive comments and suggestions, which have substantially strengthened the quality and clarity of the work. We greatly appreciate their thoughtful engagement and their support for publication.
